# Maternal Exposure to Criteria Air Pollutants and Congenital Heart Defects in Offspring: Results from the National Birth Defects Prevention Study

**DOI:** 10.1289/ehp.1307289

**Published:** 2014-04-11

**Authors:** Jeanette A. Stingone, Thomas J. Luben, Julie L. Daniels, Montserrat Fuentes, David B. Richardson, Arthur S. Aylsworth, Amy H. Herring, Marlene Anderka, Lorenzo Botto, Adolfo Correa, Suzanne M. Gilboa, Peter H. Langlois, Bridget Mosley, Gary M. Shaw, Csaba Siffel, Andrew F. Olshan, the National Birth Defects Prevention Study

**Affiliations:** 1 Department of Epidemiology, UNC Gillings School of Global Public Health, Chapel Hill, North Carolina, USA; 2 National Center for Environmental Assessment, Office of Research and Development, U.S. Environmental Protection Agency, Research Triangle Park, North Carolina, USA; 3 Department of Statistics, North Carolina State University, Raleigh, North Carolina, USA; 4 Department of Pediatrics, and; 5 Department of Pediatrics Genetics, University of North Carolina at Chapel Hill, Chapel Hill, North Carolina, USA; 6 Department of Biostatistics, Gillings School of Global Public Health, University of North Carolina at Chapel Hill, Chapel Hill, North Carolina, USA; 7 Massachusetts Center for Birth Defects Research and Prevention, Massachusetts Department of Public Health, Boston, Massachusetts, USA; 8 Department of Genetics and Pediatrics, University of Utah, Salt Lake City, Utah, USA; 9 Department of Pediatrics, University of Mississippi Medical Center, Jackson, Mississippi, USA; 10 National Center on Birth Defects and Developmental Disabilities, Centers for Disease Control and Prevention, Atlanta, Georgia, USA; 11 Texas Center for Birth Defects Research and Prevention, Texas Department of State Health Services, Austin, Texas, USA; 12 Arkansas Center for Birth Defects Research and Prevention, University of Arkansas for Medical Sciences, Little Rock, Arkansas, USA; 13 Department of Pediatrics, Stanford University School of Medicine, Stanford, California, USA

## Abstract

Background: Epidemiologic literature suggests that exposure to air pollutants is associated with fetal development.

Objectives: We investigated maternal exposures to air pollutants during weeks 2–8 of pregnancy and their associations with congenital heart defects.

Methods: Mothers from the National Birth Defects Prevention Study, a nine-state case–control study, were assigned 1-week and 7-week averages of daily maximum concentrations of carbon monoxide, nitrogen dioxide, ozone, and sulfur dioxide and 24-hr measurements of fine and coarse particulate matter using the closest air monitor within 50 km to their residence during early pregnancy. Depending on the pollutant, a maximum of 4,632 live-birth controls and 3,328 live-birth, fetal-death, or electively terminated cases had exposure data. Hierarchical regression models, adjusted for maternal demographics and tobacco and alcohol use, were constructed. Principal component analysis was used to assess these relationships in a multipollutant context.

Results: Positive associations were observed between exposure to nitrogen dioxide and coarctation of the aorta and pulmonary valve stenosis. Exposure to fine particulate matter was positively associated with hypoplastic left heart syndrome but inversely associated with atrial septal defects. Examining individual exposure-weeks suggested associations between pollutants and defects that were not observed using the 7-week average. Associations between left ventricular outflow tract obstructions and nitrogen dioxide and between hypoplastic left heart syndrome and particulate matter were supported by findings from the multipollutant analyses, although estimates were attenuated at the highest exposure levels.

Conclusions: Using daily maximum pollutant levels and exploring individual exposure-weeks revealed some positive associations between certain pollutants and defects and suggested potential windows of susceptibility during pregnancy.

Citation: Stingone JA, Luben TJ, Daniels JL, Fuentes M, Richardson DB, Aylsworth AS, Herring AH, Anderka M, Botto L, Correa A, Gilboa SM, Langlois PH, Mosley B, Shaw GM, Siffel C, Olshan AF, National Birth Defects Prevention Study. 2014. Maternal exposure to criteria air pollutants and congenital heart defects in offspring: results from the National Birth Defects Prevention Study. Environ Health Perspect 122:863–872; http://dx.doi.org/10.1289/ehp.1307289

## Introduction

Epidemiologic studies provide inconsistent evidence of an association between exposure to air pollutants and congenital heart defects (CHDs) (Agay-Shay et al. 2013; Dadvand et al. 2011a, 2011b; Dolk et al. 2010; Gilboa et al. 2005; Hansen et al. 2009; Padula et al. 2013; Rankin et al. 2009; Ritz et al. 2002; Strickland et al. 2009; Vrijheid et al. 2011). A recent meta-analysis identified two associations: nitrogen dioxide (NO_2_) exposure and tetralogy of Fallot (TOF), and sulfur dioxide (SO_2_) exposure and coarctation of the aorta (COA) (Vrijheid et al. 2011). However, only five defects/defect groupings were explored.

Most previous studies used monitoring data and assigned exposure by averaging daily pollutant averages over postconception weeks 3–8. This method does not capture the temporal variability in exposure across windows with greater impact on cardiac development, which could mask or attenuate associations. Using daily maximum concentrations, as opposed to averages, to calculate exposure would better capture daily exposure peaks and more closely parallel regulatory standards issued by the U.S. Environmental Protection Agency (U.S. EPA 2012). Teratogenic models have suggested that environmental insults have a threshold below which there is no observed impact on the fetus (Dolk and Vrijheid 2003). Based on these past models of teratogenicity, the higher exposures represented by daily maxima could be more relevant to disruption of cardiac development. Separating a single overall average into weekly averages would also allow for the exploration of specific windows of susceptibility and reduce potential misclassification of exposure.

In this study we used data from the National Birth Defects Prevention Study (NBDPS), a large population-based case–control study of birth defects, to investigate the association between CHDs in offspring and ambient concentrations of the following criteria air pollutants during early pregnancy: carbon monoxide (CO), NO_2_, ozone (O_3_), particulate matter with aerodynamic diameter ≤ 10 μm (PM_10_), particulate matter with aerodynamic diameter ≤ 2.5 μm (PM_2.5_), and SO_2_.

## Methods

*Study population*. The NBDPS recruits cases from population-based, active surveillance congenital anomaly registries in nine U.S. states and includes live births and stillbirths > 20 weeks gestation or at least 500 g, as well as elective terminations of prenatally diagnosed defects when available (Yoon et al. 2001). Arkansas, Iowa, and Massachusetts ascertain cases statewide, whereas California, Georgia, New York, North Carolina, Texas, and Utah ascertain cases in select counties. Cases are reviewed by clinical geneticists using standardized study protocols to determine study eligibility and classification, and cases with chromosomal/microdeletion disorders and disorders of known single-gene deletion causation are excluded. Controls are unaffected livebirths who are randomly selected from vital records or hospital records, depending upon study center. The NBDPS has been approved by the institutional review boards (IRBs) of all participating centers, and all participants provided written or oral informed consent before participation. These analyses were reviewed and approved by the University of North Carolina IRB.

For this analysis, the study population consisted of all controls and eligible cases with a simple, isolated CHD (i.e., a single CHD with no extra-cardiac birth defects present) and an estimated date of delivery (i.e., due date) from 1 October 1997 through 31 December 2006. During this time period, the participation response was 69% among all cases and 65% for controls. Within the NBDPS, a team of clinicians with expertise in pediatric cardiology reviewed information abstracted from the medical record and centrally assigned a single, detailed cardiac phenotype to each case whose diagnosis was confirmed by echocardiography, cardiac catheterization, surgery, or autopsy and documented in the medical record. Phenotypes were then aggregated into individual CHDs and defect groupings (Botto et al. 2007). The following additional groups were created because of limited sample size of individual defects: *a*) other conotruncal defects, which included common truncus, interrupted aortic arch–type B (IAA-type B), interrupted aortic arch–not otherwise specified (IAA-NOS), double outlet right ventricle associated with transposition of the great arteries (DORV-TGA) and not associated with TGA (DORV-other), and conoventricular septal defects (VSD-conoventricular); and *b*) atresias that included both pulmonary and tricuspid atresia. Simple, isolated CHD cases represented 64% (*n* = 12,383) of the total CHD cases. We restricted the analysis to offspring with a single CHD to create more etiologically homogeneous case groups, although this limits the generalizability of our findings. Women who reported having pregestational diabetes (types 1 and 2) during their pregnancy were excluded owing to the established association with CHD (Correa et al. 2008). Women living > 50 km from a pollutant-specific air monitor were excluded from that analysis.

*Exposure assignment*. Each woman reported the due date that was provided by her clinician during pregnancy to obtain the gestational age of the infant at birth. Using the gestational age to estimate the date of conception, we assigned calendar dates to each week of pregnancy. Women’s residential addresses during pregnancy were centrally geocoded to ensure consistency across study centers. Each geocoded address during weeks 2–8 of pregnancy was matched to the closest air monitor for each pollutant, with > 50% of the data available using ArcGISv10 (ESRI, Redlands, CA) and monitor locations obtained from U.S. EPA’s Air Quality System (U.S. EPA 2013). Participants from 1996–1998 were excluded from the analysis of PM_2.5_ because monitoring began in 1999.

We used the daily maximum hourly measurement for CO, NO_2_, and SO_2_, the daily maximum 8-hr average for O_3_, and 24-hr measurements of PM_10_ and PM_2.5_ to assign exposure. We averaged over the daily maximum or 24-hr measurements for weeks 2–8 of pregnancy to assign a 7-week and also 1-week averages of the daily values. We included week 2 in addition to the standard window of cardiac development, because of the potential for lag effects of air pollution (van den Hooven et al. 2012). If only a single measurement was taken during a given week, it was assigned as the weekly exposure. Ambient levels of each pollutant except O_3_ were categorized into the following categories, using the distribution of pollutant concentration among controls: less than the 10th centile (referent), 10th centile to less than the median, the median to less than the 90th centile, and greater than or equal to the 90th centile. These categories captured the departure from linearity observed in initial, exploratory analyses (data not shown). For similar reasons, O_3_ was categorized into quartiles. Centiles were calculated separately for the 7-week and 1-week measures of exposure.

*Statistical analysis*. The following variables obtained from the maternal interview were identified as potential confounders through directed acyclic graph analysis (Greenland et al. 1999) and included in the final adjustment set: maternal age, race/ethnicity, educational attainment, household income, tobacco smoking in the first month of pregnancy, alcohol consumption during the first trimester, and maternal nativity. Maternal age was represented as a single, continuous term, measured at the time of conception. Race/ethnicity was self-reported and categorized into the following groups: white non-Latino, black non-Latino, Latino, Asian or Pacific Islander, and other. Educational attainment was collapsed into six categories: 0–6 years of education, 7–11 years, high school graduate or equivalency, 1–3 years of college or trade school, 4 years of college or completion of a bachelor’s degree, and an advanced degree. Household income was self-reported as < $10,000 annually, > $50,000 annually, or in-between. We adjusted for any tobacco use in the first month of pregnancy and differentiated between some alcohol consumption (less than four drinks) and binge drinking (four or more drinks) during the first trimester. Maternal nativity was defined as self-report of being born outside the United States.

To account for potential differences in case ascertainment by study center, models were also adjusted for the center-specific ratio of septal defects to total CHDs. Identifying septal defects often depends on method of case ascertainment (Martin et al. 1989). All potential confounders, as well as distance to major roadway, prepregnancy body mass index (BMI), and maternal occupation status during pregnancy were assessed for effect measure modification by constructing logistic regression models with and without interaction terms and conducting likelihood ratio tests using an *a priori* alpha level of 0.1. Distance to the closest major road—defined as an interstate, U.S. highway, state, or larger county highway—was constructed using ArcGISv10 and then dichotomized at 50 m. Prepregnancy BMI was defined using self-reported maternal height and weight and categorized according to National Institutes of Health (1998) guidelines into underweight (BMI < 18.5), normal weight (18.5 ≤ BMI < 25), overweight (25 ≤ BMI < 30), and obese (BMI ≥ 30). Maternal occupation status was defined as ever working outside the home during any time during pregnancy.

For each pollutant, models were constructed to explore individual defects and defect-groupings. If a woman did not have at least one monitoring value for each week of exposure, she was excluded from the weekly analysis. We explored the relationships between all weeks and all defects because of uncertainty in pregnancy dating when using an estimated date of conception and the lack of clearly elucidated mechanisms by which cardiac development could be disrupted by exposure to air pollution. Animal research suggests that exposures outside the typical period of development for an individual heart structure could also be etiologically relevant (Morgan et al. 2008).

Because we simultaneously assessed multiple weeks of exposure and multiple defects/groupings, we constructed two-stage hierarchical regression models to account for the correlation between estimates and partially address multiple inference (Greenland 1992; Witte et al. 1998). The first-stage, represented in Equation 1, was an unconditional, polytomous logistic regression model of individual CHDs on exposure (***x***) defined as either all 1-week averages of maximum or 24-hr pollutant values or the single 7-week average, and the full adjustment set (***w***) detailed above.


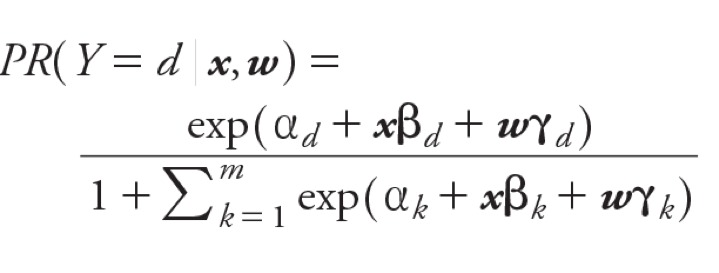
[1]

**b***_d_* is the vector of regression coefficients corresponding to pollutant exposure for an individual CHD (*d*), **c***_d_* is the vector of regression coefficients corresponding to the covariates for a given defect, and *m* is the total number of individual types of CHDs. The second-stage model, which defines how the first-stage betas are associated, is given in Equation 2:

β*_i_* = ***Z_i_*r** + δ*_i_*, [2]

where ***Z_i_*** is a row in the design matrix that includes an intercept term and then indicator variables for type of defect, broader defect grouping, and exposure week/level for the *i*th β, **r** is the vector of coefficients corresponding to the variables included in the design matrix, and δ*_i_* are independent normal random variables with a mean of zero and a variance of τ^2^ that describe the residual variation in β*_i_*. The obtained second-stage coefficients, **r**, are used to estimate values toward which the first-stage coefficients will be shrunk, with the magnitude of the shrinkage depending on the precision of the maximum-likelihood estimate obtained in stage 1 and the value of the second-stage variance, τ^2^ (Greenland 1992; Witte et al. 1998). We fixed τ^2^ at 0.5, corresponding to a prior belief with 95% certainty that the residual odds ratio (OR) will fall within a 16-fold span.

To assess whether our results were robust to changes in model specification, we conducted sensitivity analyses by setting the value of τ^2^ to 0.25, corresponding to a 7-fold OR span, as well as to a value of 1, corresponding to a 50-fold span. We also explored different specifications for the design matrix, in turn defining the prior value as a common mean for all defects, a common mean for each defect, or a common mean for each exposure week/level, across defects. Individual defects with > 10 but < 100 cases were excluded from hierarchical models and explored using Firth’s penalized maximum-likelihood method to address the quasi-complete separation that occurred due to small sample size (Heinze and Schemper 2002). These defects included the individual defects collapsed into the other conotruncals and atresia categories described above; Ebstein’s anomaly, which was part of the right ventricular outflow tract obstruction (RVOTO) defect grouping; and muscular ventricular septal defects (VSD_muscular_), which was part of the septal defect-grouping. IAA-type A and partial anomalous pulmonary venous return had < 10 cases each and were excluded from all individual analyses, but were included in the left ventricular outflow tract obstruction (LVOTO) and anomalous pulmonary venous return (APVR) defect groupings, respectively. To assess whether pollutant–defect relationships conformed to a monotonic dose response, we reanalyzed the data using incremental coding which compares each category of exposure to its immediate predecessor. If the incremental ORs are all above (or below) 1, the relationship conforms to a monotonic dose response (Maclure and Greenland 1992).

To explore associations with CHDs within a multipollutant context, a principal component analysis (PCA) was conducted among participants who lived within 50 km of each type of monitor. PCA is used to reduce the number of correlated variables into a smaller number of artificial variables that capture most of the variance of the original variables while being uncorrelated with each other (Hatcher 1994). This allows the resulting factors to be included within the same model, reducing issues of multicollinearity. Applying PCA, we retained components that accounted for at least the same or more variance than one of the original pollutant variables. We then applied a varimax rotation and calculated factor scores for each participant. These factor scores were categorized using the 10th, 50th, and 90th centiles and used to assign exposure in hierarchical models.

## Results

Demographics of the NBDPS controls and CHD defect groupings providing residential address information and eligible to be matched to the closest air monitor for each pollutant are presented in Table 1. Case distribution varied by study site, particularly for the septal defect grouping. The ratios used to adjust for case-ascertainment differences by site are located in the Supplemental Material, Table S1. The percentage of women who lived 50 km from an air pollution monitor varied from 56% for SO_2_ to 73% for PM_10_. Demographics were similar across the pollutant-specific populations, although women who lived within 50 km of a SO_2_ monitor were slightly older and were more likely to be white or African American, work outside the home, have higher household income, and report alcohol consumption during pregnancy (data not shown). The number of cases/controls by exposure distribution for each pollutant are represented in Table 2, along with the pollutant levels that were used to define exposure categories. Median distance to the monitor was similar across pollutants, although women tended to live farther from SO_2_ monitors and closer to PM_2.5_ monitors.

**Table 1:** Demographic characteristics of geocoded, nonpregestational diabetic population of CHD groupings and controls, National Birth Defects Prevention Study (1997–2006).

Demographics	Controls	APVR	AVSD	Conotruncal	LVOTO	RVOTO	Septal
Site
Arkansas	611 (9.7)	18 (11.6)	10 (12.2)	89 (8.9)	66 (8.0)	110 (15.1)	321 (17.4)
California	871 (13.8)	27 (17.4)	5 (6.1)	159 (15.8)	111 (13.4)	60 (8.3)	110 (6.0)
Iowa	806 (12.7)	10 (6.5)	15 (18.3)	96 (9.6)	122 (14.7)	95 (13.1)	189 (10.2)
Massachusetts	916 (14.5)	28 (18.1)	15 (18.3)	187 (18.7)	117 (14.1)	121 (16.6)	240 (13.0)
Metro Atlanta, Georgia	750 (11.9)	19 (12.3)	11 (13.4)	140 (13.9)	99 (11.9)	92 (12.6)	231 (12.5)
New York	637 (10.1)	13 (8.4)	7 (8.5)	110 (11.0)	86 (10.4)	68 (9.3)	125 (6.8)
North Carolina	452 (7.1)	8 (5.2)	4 (4.9)	56 (5.6)	27 (3.3)	33 (4.5)	74 (4.0)
Texas	815 (12.9)	20 (12.9)	7 (8.5)	115 (11.5)	88 (10.6)	66 (9.1)	439 (23.8)
Utah	470 (7.4)	12 (7.7)	8 (9.8)	52 (5.2)	113 (13.6)	83 (11.4)	119 (6.5)
Race/ethnicity
White, non-Latino	3,797 (60.0)	89 (57.4)	62 (75.6)	612 (61.0)	592 (71.4)	474 (65.1)	1,032 (55.8)
Black, non-Latino	682 (10.8)	9 (5.8)	10 (12.2)	102 (10.2)	50 (6.0)	98 (13.5)	238 (12.9)
Latino	1,460 (23.1)	44 (28.4)	4 (4.9)	221 (22.0)	159 (19.2)	116 (15.9)	467 (25.3)
Asian/Pacific Islander	168 (2.7)	7 (4.5)	3 (3.7)	35 (3.5)	13 (1.6)	13 (1.8)	53 (2.9)
Other	219 (3.5)	6 (3.9)	3 (3.7)	34 (3.4)	15 (1.8)	27 (3.7)	58 (3.1)
Education
0–6 years	210 (3.3)	7 (4.6)	1 (1.2)	40 (4.0)	27 (3.3)	19 (2.6)	58 (3.1)
7–11 years	844 (13.4)	25 (16.3)	6 (7.3)	121 (12.1)	99 (12.0)	81 (11.1)	293 (15.9)
High school diploma or equivalent	1,516 (24.1)	37 (24.2)	20 (24.4)	239 (23.9)	186 (22.5)	196 (26.9)	452 (24.5)
1–3 years college or trade school	1,726 (27.4)	42 (27.5)	29 (35.4)	276 (27.6)	227 (27.4)	196 (26.9)	551 (29.8)
4 years college or Bachelors degree	1,414 (22.5)	30 (19.6)	20 (24.4)	229 (22.9)	216 (26.1)	181 (24.9)	367 (19.9)
Advanced degree	581 (9.2)	12 (7.8)	6 (7.3)	95 (9.5)	73 (8.8)	55 (7.6)	126 (6.8)
Maternal age [years (mean ± SD)]	27.0 ± 6.1	26.7 ± 6.7	26.9 ± 5.3	27.8 ± 6.2	27.8 ± 5.8	27.7 ± 6.1	27.0 ± 6.5
Nativity
Born in United States	5,110 (81.2)	118 (77.1)	70 (85.4)	804 (80.4)	697 (84.2)	633 (87.0)	1,527 (82.7)
Household income
< $10,000	1,066 (18.5)	26 (19.1)	13 (16.5)	167 (17.7)	105 (13.5)	109 (16.1)	351 (20.4)
$10,000–$50,000	2,695 (46.7)	69 (50.7)	41 (51.9)	410 (43.4)	368 (47.2)	321 (47.4)	853 (49.7)
> $50,000	2,012 (34.9)	41 (30.2)	25 (31.7)	367 (38.9)	306 (39.3)	248 (36.6)	514 (29.9)
Occupational status
Worked outside home	4,545 (72.1)	97 (63.4)	70 (85.4)	742 (74.2)	604 (72.9)	544 (74.7)	1,279 (69.3)
Smoking
First month	967 (15.3)	26 (17.0)	26 (31.7)	140 (14.0)	114 (13.8)	122 (16.8)	373 (20.2)
Alcohol consumption
No drinking	3,981 (63.6)	101 (67.3)	50 (61.0)	603 (60.9)	550 (66.9)	473 (66.2)	1,210 (65.9)
< 4 drinks	1,509 (24.1)	31 (20.7)	19 (23.2)	251 (25.3)	165 (20.1)	164 (22.9)	405 (22.1)
≥ 4 drinks	770 (12.3)	18 (12.0)	13 (15.9)	137 (13.8)	107 (13.0)	78 (10.9)	222 (12.1)
BMI
< 18.5 (underweight)	316 (5.2)	8 (5.4)	4 (5.0)	50 (5.2)	34 (4.3)	25 (3.6)	96 (5.4)
18.5 to < 25 (normal)	3,373 (55.4)	79 (53.7)	46 (57.5)	519 (53.5)	426 (53.8)	330 (46.9)	910 (51.0)
25 to < 30 (overweight)	1,404 (23.1)	31 (21.1)	18 (22.5)	221 (22.8)	182 (23.0)	190 (27.0)	425 (23.8)
BMI ≥ 30 (obese)	993 (16.3)	29 (19.7)	12 (15.0)	180 (18.6)	150 (18.9)	159 (22.6)	354 (19.8)
Proximity to roadway
< 50 m	1,168 (18.5)	37 (23.9)	14 (17.1)	192 (19.1)	156 (18.8)	112 (15.4)	331 (17.9)
Values are *n* (%) unless otherwise noted.							

**Table 2:** CHD cases and controls by exposure level of criteria air pollutants, National Birth Defects Prevention Study (1997–2006; except for PM_2.5_ 1999–2006).

Pollutant and outcome	< 10th centile	10th to < 50th centile	50th to < 90th centile	≥ 90th centile	Distance to monitor 25th, 50th, 75th centile (km)
CO (ppm)	< 0.58	0.58 to < 1.16	1.16 to < 2.13	≥ 2.13	7.0, 14.8, 26.5
Controls (*n*)	434	1,740	1,739	436
All cases (*n*)	271	1,202	1,235	308
LVOTO (*n*)^*a*^	53	249	229	49
Aortic stenosis (*n*)	12	50	45	10
COA (*n*)	22	106	80	21
HLHS (*n*)	18	91	102	17
Conotruncal	66	305	312	70
dTGA	22	102	102	21
TOF	33	162	167	37
Other conotruncals	11	41	43	12
APVR^*b*^	17	42	36	10
TAPVR	15	42	29	10
AVSD	5	20	25	3
RVOTO^*c*^	46	202	207	47
Pulmonary/tricuspid atresia	12	41	39	9
PVS	33	142	154	36
Septal^*d*^	84	382	424	128
VSD_pm_	47	185	215	54
ASD	36	172	159	49
NO_2_ (ppb)	< 18.9	18.9 to < 33.3	33.3 to < 45.5	≥ 45.5	6.8, 13.7, 25.1
Controls (*n*)	396	1,584	1,591	397
All cases (*n*)	248	1,088	1,152	309
LVOTO^*a*^ (*n*)	43	211	235	56
Aortic stenosis (*n*)	7	47	42	14
COA (*n*)	12	74	103	26
HLHS (*n*)	23	86	89	16
Conotruncal	58	277	285	71
dTGA	23	92	99	24
TOF	27	150	140	38
Other conotruncals	8	35	46	9
APVR^*b*^	16	36	35	13
TAPVR	15	33	32	13
AVSD	9	18	22	4
RVOTO^*c*^	38	164	194	63
Pulmonary/tricuspid atresia	6	41	34	9
PVS	32	109	143	50
Septal^*d*^	84	380	379	101
VSD_pm_	43	178	189	51
ASD	36	163	161	35
O_3_ (ppb)^*e*^	< 32.2	32.2 to < 42.9	42.9 to < 51.8	≥ 51.8	6.8, 12.8, 21.9
Controls (*n*)	442	1,769	1,768	443
All cases (*n*)	308	1,311	1,204	269
LVOTO^*a*^ (*n*)	60	228	223	55
Aortic stenosis (*n*)	9	47	48	8
COA (*n*)	23	86	87	27
HLHS (*n*)	27	92	85	20
Conotruncal	85	300	283	68
dTGA	31	92	112	19
TOF	42	169	135	40
Other conotruncals	12	39	36	9
APVR^*b*^	8	45	47	12
TAPVR	7	41	45	11
AVSD	6	17	22	4
RVOTO^*c*^	38	196	202	51
Pulmonary/tricuspid atresia	7	41	40	10
PVS	25	142	147	36
Septal^*d*^	109	523	427	79
VSD_pm_	47	203	200	45
ASD	44	279	196	31
PM_10_ (μg/m^3^)	< 14.9	14.9 to < 24.2	24.2 to < 40.6	≥ 40.6	6.0, 13.5, 25.2
Controls (*n*)	462	1,853	1,853	464
All cases (*n*)	298	1,377	1,387	271
LVOTO^*a*^ (*n*)	54	229	276	52
Aortic stenosis (*n*)	12	54	63	8
COA (*n*)	15	97	97	22
HLHS (*n*)	24	76	115	21
Conotruncal	64	295	311	87
dTGA	25	97	98	32
TOF	33	150	175	43
Other conotruncals	6	48	38	12
APVR^*b*^	8	52	45	13
TAPVR	8	45	39	13
AVSD	2	25	24	4
RVOTO^*c*^	55	202	225	40
Pulmonary/tricuspid atresia	16	40	46	6
PVS	33	151	164	29
Septal^*d*^	115	572	503	75
VSD_pm_	44	227	214	37
ASD	56	292	233	36
PM_2.5_ (μg/m^3^)	< 7.77	7.77 to < 12.1	12.1 to < 19.7	≥ 19.7	5.3, 10.4, 20.7
Controls (*n*)	440	1,763	1,763	441
All cases (*n*)	378	1,420	1,212	301
LVOTO^*a*^ (*n*)	66	250	207	73
Aortic stenosis (*n*)	21	61	39	14
COA (n)	28	92	88	25
HLHS (*n*)	15	95	77	33
Conotruncal	71	287	291	87
dTGA	25	90	95	25
TOF	35	150	161	50
Other conotruncals	11	47	35	12
APVR^*b*^	14	51	36	13
TAPVR	12	46	32	11
AVSD	3	26	27	6
RVOTO^*c*^	58	206	229	47
Pulmonary/tricuspid atresia	14	46	34	11
PVS	39	143	178	35
Septal^*d*^	166	600	418	75
VSD_pm_	49	229	222	38
ASD	115	369	189	36
SO_2_ (ppb)	< 3.45	3.45 to < 9.7	9.7 to < 19.9	≥ 19.9	8.9, 18.8, 30.2
Controls (*n*)	350	1,403	1,404	351
All cases (*n*)	231	1,048	1,098	240
LVOTO^*a*^ (*n*)	33	190	200	39
Aortic stenosis (*n*)	9	39	32	7
COA (*n*)	13	69	92	21
HLHS (*n*)	10	81	72	11
Conotruncal	48	221	258	60
dTGA	16	76	87	21
TOF	24	117	133	33
Other conotruncals	8	28	38	6
APVR^*b*^	9	33	35	7
TAPVR	9	27	32	6
AVSD	3	14	21	8
RVOTO^*c*^	26	203	183	31
Pulmonary/tricuspid atresia	8	37	35	5
PVS	15	155	135	22
Septal^*d*^	112	387	398	93
VSD_pm_	33	164	192	49
ASD	76	196	151	29
Abbreviations: APVR, anomalous pulmonary venous return; ASD, atrial septal defect; AVSD, atrioventricular septal defect; COA, coarctation of the aorta; dTGA, transposition of the great arteries; HLHS, hypoplastic left heart syndrome; LVOTO, left ventricular outflow tract obstructions; PVS, pulmonary valve stenosis; RVOTO, right ventricular outflow tract obstructions; TAPVR, total anomalous pulmonary venous return; TOF, tetralogy of Fallot; VSD_pm_, perimembranous ventricular septal defects.^***a***^LVOTO grouping also includes cases of interrupted aortic arch–type A, which was not analyzed individually due to limited sample size. ^***b***^APVR grouping also includes cases of partial anomalous pulmonary venous return, which was not analyzed individually due to limited sample size. ^***c***^RVOTO grouping also includes cases of Ebstein’s anomaly, which was not analyzed individually in the hierarchical analysis due to limited sample size. ^***d***^Septal grouping also includes cases of muscular ventricular septal defects (VSD_muscular_), which was not analyzed individually in the hierarchical analysis due to limited sample size. The exception is PM_2.5_: VSD_muscular_ were collected only in the first year of the study when PM_2.5_ measurements were not available. ^***e***^O_3_ exposure was categorized into quartiles using the distribution among the controls. The referent was < 25th percentile, and the other 3 categories were 25 to < 50, 50 to < 75, and ≥ 75.					

**Figure 1:**
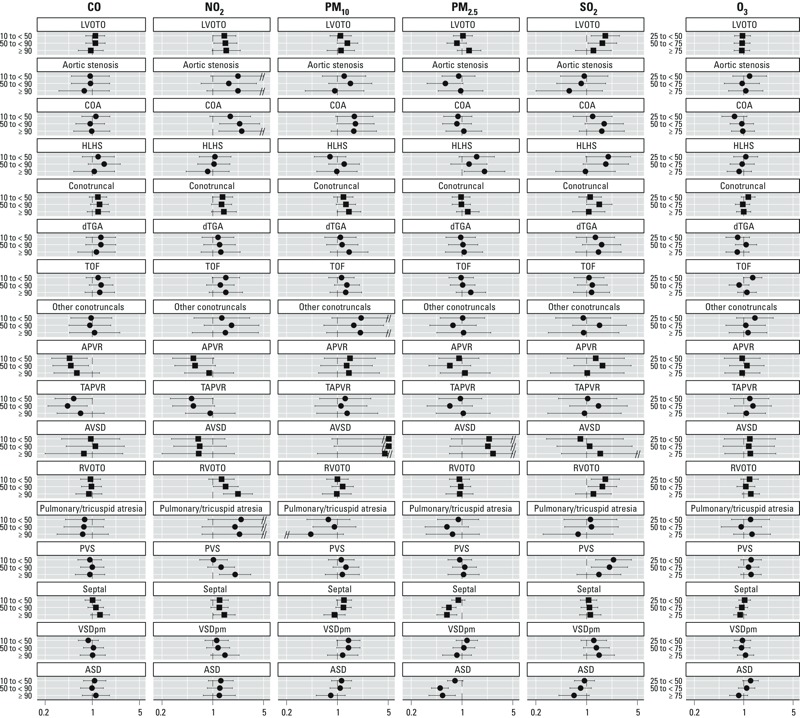
Estimated adjusted ORs and 95% CIs between CHDs and 7-week average of daily maxima/24-hr measures of criteria air pollutants, National Birth Defects Prevention Study 1997–2006 (for PM_2.5_, 1999–2006). Abbreviations: APVR, anomalous pulmonary venous return; ASD, atrial septal defect; AVSD, atrioventricular septal defect; COA, coarctation of the aorta; dTGA, transposition of the great arteries; HLHS, hypoplastic left heart syndrome; LVOTO, left ventricular outflow tract obstructions; PVS, pulmonary valve stenosis; RVOTO, right ventricular outflow tract obstructions; TAPVR, total anomalous pulmonary venous return; TOF, tetralogy of Fallot; VSD_pm_, perimembranous ventricular septal defects. Other conotruncal category includes common truncus, interrupted aortic arch–type B and type not specified, double outlet right ventricle defects, and conoventricular septal defects. A double slash (//) indicates truncation of the results. Squares indicate defect groupings; circles indicate individual defects. Defect groupings include all individual defects listed underneath with the following additions: LVOTO, IAA‑type A; APVR, partial APVR; RVOTO, Ebstein’s anomaly; Septal, muscular venricular septal defects (VSD_muscular_), except for PM_2.5_. VSD_muscular_ were collected only in the first year of study when no PM_2.5_ data were available. Those defects could not be analyzed within the hierarchical regression due to limited sample size. ORs were estimated from hierarchical regression models. First stage was a polytomous logistic model, adjusted for maternal race/ethnicity, age educational attainment, household income, maternal smoking status and alcohol consumption during early pregnancy, nativity, and site-specific heart defect ratio. Second stage was a linear model with indicator variables for defect, defect grouping, and level of exposure. For all pollutants except O_3_, the three categories of exposure are as follows: 10th centile to < 50th centile, 50th centile to < 90th centile, and ≥ 90th centile, with the referent level being < 10th centile among controls. For ozone, the three categories of exposure were 25th to < 50th centile, 50th centile to < 75th centile, and ≥ 75th centile, with the referent grouping being below the 25th centile. Pollutant levels that define the category cut points are provided in Table 2. See Supplemental Material, Table S2, for corresponding numeric data.

*Exposure assigned as a single 7-week average of daily maxima or 24-hr measurements*. Figure 1 shows the estimated adjusted ORs and 95% CIs resulting from the hierarchical regression models of the 7-week average exposure to individual pollutants and CHDs (see Supplemental Material, Table S2, for corresponding numerical data). Crude estimates were similar to estimates adjusted for confounders (data not shown). Larger ORs were observed with greater NO_2_ exposure for individual defects within the LVOTO and RVOTO groupings. Women with the highest average daily maximum exposure to NO_2_ (> 45.5 ppb) had more than two times the odds of both COA (OR = 2.5; 95% CI: 1.21, 5.18) and PVS (OR = 2.03; 95% CI: 1.23, 3.33) as women with the lowest exposure (< 18.9 ppb). We observed a positive association between SO_2_ exposure and PVS, although it was attenuated at the highest exposure level (OR for 10th–50th/10th centile contrast = 2.34; 95% CI: 1.33, 4.14; OR for 50th–90th/10th centile contrast = 2.06; 95% CI: 1.16, 3.67; OR for 90th/10th centile contrast = 1.48; 95% CI: 0.74, 2.97). Hypoplastic left heart syndrome (HLHS) was associated with exposure to PM_2.5_ (90th/10th centile contrast: OR = 2.04; 95% CI: 1.07, 3.89) but not NO_2_. We observed increased odds of perimembranous ventricular septal defects (VSD_pm_) (OR for 90th/10th centile contrast = 1.48; 95% CI: 0.91, 2.42) and reduced odds of atrial septal defects (ASD) (OR for 90th/10th centile contrast = 0.67; 95% CI: 0.41, 1.09) with SO_2_ exposure_._ We also observed reduced odds of ASDs with exposure to PM_2.5_ (OR for 50th–90th/10th contrast = 0.50; 95% CI: 0.38, 0.65; OR for 90th/10th contrast = 0.54; 95% CI: 0.35, 0.81). Although imprecise, the effect estimates for APVR and CO and NO_2_ exposures indicated lower odds with greater exposure, although the negative association was attenuated at the highest exposure level. The associations between NO_2_ and PVS, NO_2_ and COA, SO_2_ and VSD_pm_, and SO_2_ and ASDs increased monotonically with increasing exposure (data not shown). For both PM_10_ and NO_2_, we found evidence of effect measure modification by distance to a major road in first-stage maximum likelihood models, using our *a priori* criterion of a likelihood ratio test *p*-value < 0.1 (PM_10_ likelihood ratio test: χ^2^ = 30.5, *p* = 0.03; NO_2_ likelihood ratio test: χ^2^ = 34.5, *p* = 0.01). In both cases, ORs were generally greater for women who lived within 50 m of a roadway (see Supplemental Material, Table S3).

**Figure 2:**
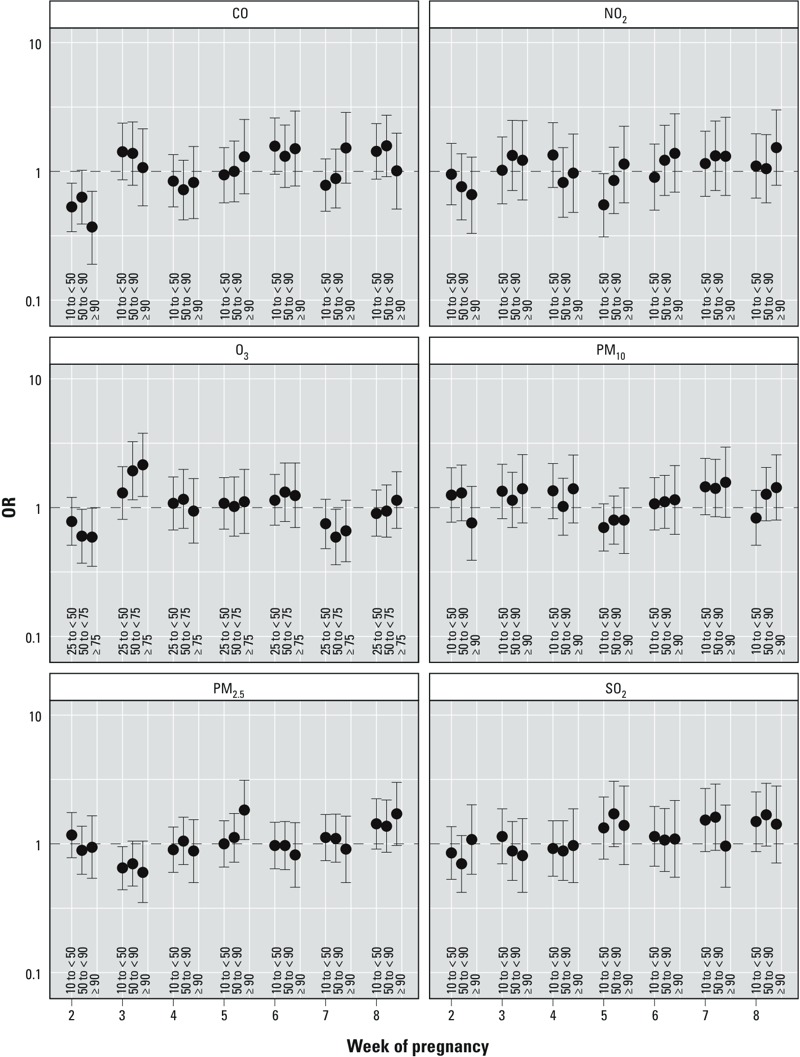
Estimated adjusted ORs and 95% CIs of pulmonary valve stenosis for categorical measures of 1-week averages of daily maxima/24-hr measures of criteria air pollutants, plotted for weeks 2–8 of pregnancy, National Birth Defects Prevention Study 1997–2006 (for PM_2.5_, 1999–2006). ORs were estimated from hierarchical regression models. First stage was a polytomous logistic model, adjusted for maternal race/ethnicity, age, educational attainment, household income, maternal smoking status and alcohol consumption during early pregnancy, nativity, and site-specific heart defect ratio. Second stage was a linear model with indicator variables for defect, defect grouping, and level of exposure. For all pollutants except O_3_, the three categories of exposure are as follows: 10th centile to < 50th centile, 50th centile to < 90th centile, and ≥ 90th centile, with the referent level being < 10th centile among controls. For O_3_, the three categories of exposure were 25th to < 50th centile, 50th centile to < 75th centile, and ≥ 75th centile, with the referent grouping being < 25th centile. Pollutant levels that define the category cut points are provided in Table 2. See Supplemental Material, Table S4, for corresponding numeric data.

*Exposure assigned as 1-week average of daily maxima or 24-hr measurements*. Full results for the weekly exposure analyses are provided in Supplemental Material, Table S4. PVS showed variability within the window of cardiac development for multiple pollutants (Figure 2). Both CO and O_3_ had individual weeks where the estimates were larger in magnitude than estimates obtained using the summary exposure and where the other weeks were closer to null, suggesting a period of greater susceptibility (CO, week 2: 90th/10th centile comparison: OR = 0.37; 95% CI: 0.19, 0.7; O_3_, week 3: 75th/25th centile comparison: OR = 2.15; 95% CI: 1.22, 3.78). PM_2.5_ had no association with PVS when a summary measure of exposure was used, but there was an almost doubling of odds in week 5 when comparing women with exposure greater than the 90th centile to women with exposure less than the 10th centile (OR = 1.83; 95% CI: 1.08, 3.12) that was similarly observed in week 8.

Week 2 of pregnancy was another potential window of susceptibility to PM_2.5_. Women having a child with TOF had almost twice the odds of being above the 90th centile versus below the 10th centile for PM_2.5_ exposure in week 2 of pregnancy as controls (OR = 1.96; 95% CI: 1.11, 3.46), whereas women with a baby with atrioventricular septal defect (AVSD) had more than three times the odds (OR = 3.43; 95% CI: 1.36, 8.66). Women with offspring with defects within the septal grouping were less likely to have higher PM_2.5_ exposure during this time (90th/10th centile comparison OR = 0.6; 95% CI: 0.4, 0.9). Using the summary exposure revealed a slightly elevated OR for VSD_pm_ among women with SO_2_ exposure greater than the 90th centile (OR = 1.48; 95% CI: 0.91, 2.42), but weekly analysis revealed this association was limited to week 3, and the magnitude increased (VSD_pm_ OR = 1.98; 95% CI: 1.1, 3.56). During other weeks, the ORs for VSD_pm_ comparing the 90th centile to the 10th centile ranged from 0.77 to 1.13.

*PCA.* Only 26% of the geocoded population (*n* = 2,914) had exposure data for all pollutants. These women were primarily from the Massachusetts and Atlanta, Georgia, sites, nonsmokers, and living in a higher-income household. African-American women made up a slightly larger percentage of these women when compared with the individual pollutant populations (data not shown). With this subsample, three factors emerged from the PCA. The factor that explained the largest amount of variance was loaded primarily by CO and NO_2_, gaseous pollutants likely related to direct emissions from local sources such as motor vehicle traffic. The second factor, driven by PM_10_, PM_2.5_, and O_3_, represents local particulates and secondary pollutant generation. The third factor was loaded by SO_2_ and most likely represents emissions from regional sources, potentially from coal combustion.

**Figure 3:**
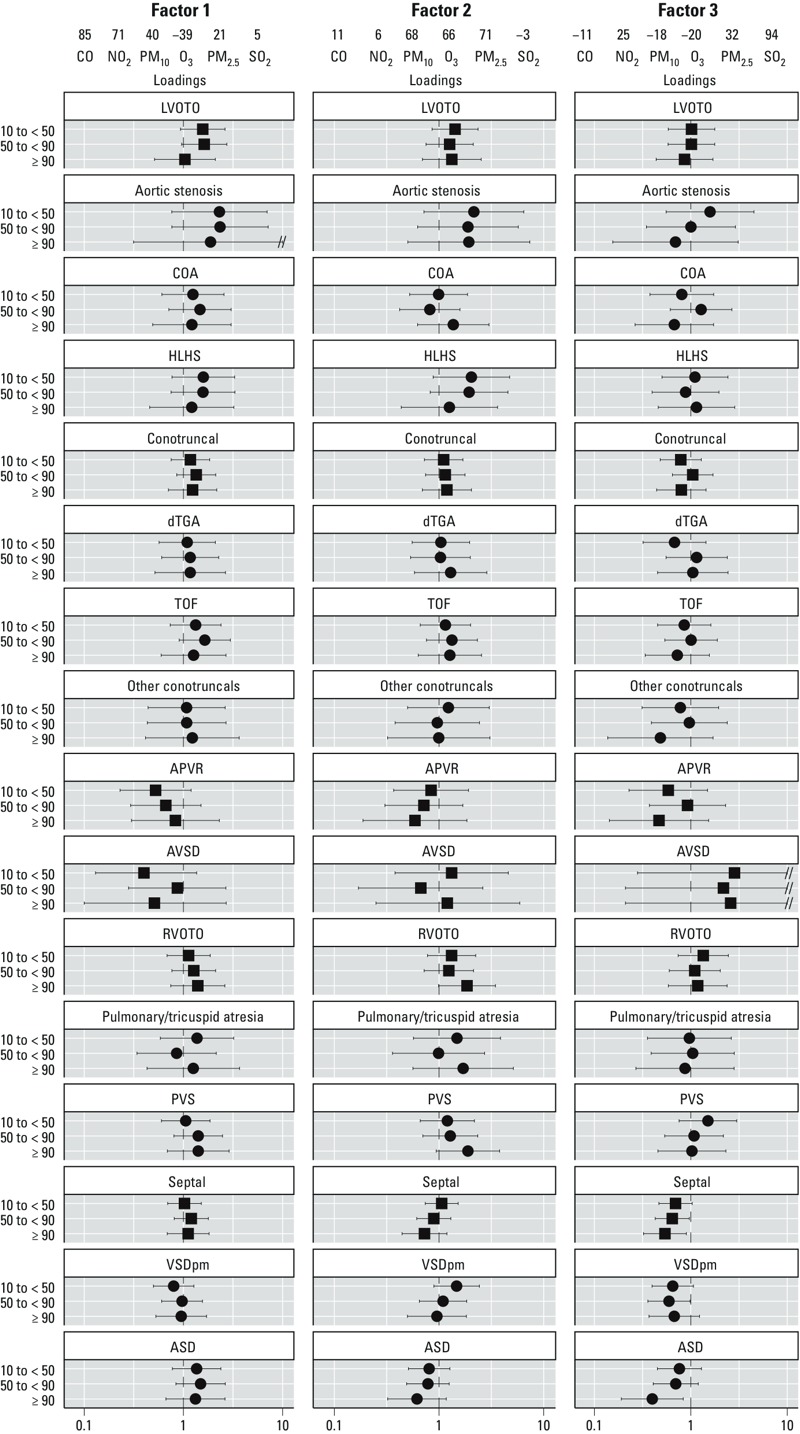
Estimated adjusted ORs and 95% CIs between CHDs and pollutant factors identified through PCA within the National Birth Defects Prevention Study, 1999–2006. Abbreviations: APVR, anomalous pulmonary venous return; ASD, atrial septal defect; AVSD, atrioventricular septal defect; COA, coarctation of the aorta; dTGA, transposition of the great arteries; HLHS, hypoplastic left heart syndrome; LVOTO, left ventricular outflow tract obstructions; PVS, pulmonary valve stenosis; RVOTO, right ventricular outflow tract obstructions; TOF, tetralogy of Fallot; VSD_pm_, perimembranous ventricular septal defects. Other conotruncal category includes common truncus, IAA‑type B and -NOS, double outlet right ventricle defects, and conoventricular septal defects. A double slash (//) indicates truncation of the results. Squares indicate defect groupings; circles indicate individual defects. Defect groupings include all individual defects listed underneath with the following additions: LVOTO, IAA‑type A; APVR, total and partial APVR; RVOTO, Ebstein’s anomaly. Those defects could not be analyzed within the hierarchical regression due to limited sample size. Loadings represent the relative weight of each of the original pollutant variables used to obtain the value of the computed factor. ORs were estimated from hierarchical regression models. First stage was a polytomous logistic model, adjusted for maternal race/ethnicity, age educational attainment, household income, maternal smoking status and alcohol consumption during early pregnancy, nativity, and site-specific heart defect ratio. Second stage was a linear model with indicator variables for defect, defect grouping, and level of exposure. For all factors, the three categories of exposure are as follows: 10th centile to < 50th centile, 50th centile to < 90th centile, and ≥ 90th centile, with the referent level being < 10th centile among controls. Pollutant levels which define the category cutpoints are provided in Table 2. See Supplemental Material, Table S5, for corresponding numeric data.

Findings were less precise than single-pollutant models due to the reduced sample size (Figure 3; see also Supplemental Material, Table S5, for corresponding numeric data). We observed ORs > 1 for the NO_2_ loaded factor (factor 1) and LVOTO defects, particularly aortic stenosis and HLHS and the PM_10_/PM_2.5_/O_3_ factor (factor 2) and HLHS, although these associations were diminished or absent at the highest exposure level. The ORs for the NO_2_ loaded factor (factor 1) and PVS were attenuated when compared with results from the NO_2_ single-pollutant model. We also observed monotonically increasing ORs between PVS and exposure to the PM_10_/PM_2.5_/O_3_ factor (factor 2), which was not observed in any of the single-pollutant models for those individual pollutants. Within the multipollutant context, the SO_2_ loaded factor (factor 3) was inversely associated with the septal defect grouping, as well as both ASD and VSD_pm_. In the single-pollutant models, we observed a slight inverse association with ASD, but a slightly positive association with VSD_pm_. The slightly increased ORs for SO_2_ exposure and PVS and HLHS observed in the single-pollutant model were not observed in the results from the PCA.

The sensitivity analysis to explore the effects of model specification did not show a material difference in results obtained when using different values of second-stage variance or varying factors defining the predicted values (data not shown). To explore our choice of a 50-km buffer size, we restricted our analyses to women who lived within 10 km of a monitor and used the same exposure categories and model construction described previously (see Supplemental Material, Table S6). Sample size was reduced to 27.5–48.1% (*n* = 1,683–3,709) of the original study population depending on pollutant. Despite the greater imprecision, many estimates remained similar: For example, the observed positive associations in the full population between higher exposure to NO_2_ and LVOTO (OR = 1.53; 95% CI: 0.98, 2.39) and RVOTO defects (OR = 2.22; 95% CI: 1.40, 3.52) were only slightly changed when restricting to the population within 10 km of an air monitor (LVOTO OR = 1.44; 95% CI: 0.58, 3.61; RVOTO OR = 2.33; 95% CI: 0.75, 7.22). The inverse association between PM_2.5_ exposure and the septal defect grouping also remained consistent after limiting the population. Although most null estimates remained so, some null estimates increased in magnitude, suggesting a potential for an association in the restricted population. For example, the OR for LVOTO defects comparing the highest and lowest quartiles of O_3_ exposure was 0.94 in the population within 50 km of a monitor (95% CI: 0.73, 1.22) but was 1.62 (95% CI: 0.84, 3.13) in the population within 10 km of a monitor. A similar increase in magnitude was observed for PM_2.5_ and LVOTO defects. The estimates related to SO_2_ exposure changed the most, with multiple ORs > 1 in the population of women living within 50 km of a monitor crossing over the null when the population was restricted to those within 10 km.

## Discussion

We found that the odds of several CHDs were higher among women with greater exposures to criteria air pollutants. We observed monotonically increasing associations between NO_2_ exposure and both COA and PVS. We also observed that women with a child with HLHS were two times as likely to live in an area with the highest level of PM_2.5_ exposure as women whose child did not have a CHD, although a similar association was not seen for women in the middle-high exposure level. Using 1-week averages, we observed temporal variability in odds of certain CHDs within the window of cardiac development. Marked by positive or negative associations in individual weeks with near null relationships in the other weeks, this pattern was observed for AVSD, PVS, TOF, and the septal defect grouping when looking across weeks of PM_2.5_ exposure, PVS when examining weeks of O_3_ exposure, and VSD_pm_ across weeks of SO_2_ exposure, although we did not observe a consistent week of greater susceptibility across different defects and pollutants.

Our findings suggest preliminary evidence that there may be periods of higher or lower susceptibility within the window of cardiac development. Embryological evidence indicates the timing of specific stages of cardiac development, beginning with the migration of cells to form the endocardial tubes and culminating with the septation of the ventricles and outflow tracts in weeks 7 and 8 of development (Gittenberger-de Groot et al. 2005). However, there is experimental research showing that triggering oxidative stress in diabetic mice can result in apoptosis among migrating neural crest cells, which later results in outflow tract defects (Morgan et al. 2008), and that neural crest cells enable the endocardial cushions to form the cardiac valves (Jain et al. 2011). This suggests it is possible that pollutant-induced oxidative stress in earlier weeks of development can trigger similar disruptions in neural crest cells that later affect development of cardiac structures, and that windows of susceptibility to environmental insults may not always directly coincide with the established stages of fetal heart development. Further research is needed to explore how timing of exposure within this narrow window may affect the risk of CHDs or whether the fluctuations in results we observed when examining weekly exposure are attributable to random noise.

Findings from the PCA-based analysis continued to show greater odds of certain CHDs with increasing pollutant exposure. The inverse association between SO_2_ and ASDs observed in the single-pollutant analysis was also observed in the PCA-based analysis. However, the positive associations between exposure to SO_2_ and PVS and VSD_pm_ found in the single-pollutant analysis were not observed when the SO_2_-loaded component was examined simultaneously with other pollutant components. These differences could be attributable to co-pollutants not accounted for in the single-pollutant models or to different demographics of the subsample of women with data on all pollutants. We often observed a decrease in odds at the highest ambient level, compared with the median-high group, in both the PCA-based analyses and single-pollutant models. Ritz (2010) has previously suggested that this nonlinearity could be attributable to differential pregnancy loss at very high exposures. It is also possible that women who live in highly polluted areas spend less time outdoors, causing exposure to be lower than what the ambient level suggests.

Our findings were consistent with the primary associations reported in the previous meta-analysis (Vrijheid et al. 2011): NO_2_ and TOF, and SO_2_ and COA, as well as an association between greater NO_2_ exposure and COA, which was suggested in the meta-analysis, although not robust to the exclusion of the largest study. We observed some of the findings from individual studies that were not identified in the meta-analysis; for example, we observed the association between SO_2_ and VSDs reported by Gilboa et al. (2005) and the inverse association between PM_2.5_ and ASDs reported by Padula et al. (2013), but not other findings such as the inverse associations between SO_2_ and conotruncal defects reported by both Gilboa et al. (2005) and Hansen et al. (2009). Differences in findings between studies could be attributable to spatial variation in source of pollutants and composition of particulates, as well as differences in case ascertainment and exposure assignment (Vrijheid et al. 2011).

This study has a number of strengths, including the large geographic scope and sample size of the NBDPS that allows analysis of systematically classified individual CHDs, while limiting analyses to simple, isolated defects to avoid heterogeneity from etiologies of multiple defects. Including live births, fetal deaths, and elective terminations prevents incomplete case ascertainment, and collecting complete residential history avoids misclassification of exposure due to using residence at delivery (Miller et al. 2010). We explored timing of exposure within the critical window of heart development and used daily maxima so as not to smooth over potentially relevant variability in exposure. Using hierarchical regression allowed us to improve estimation and partially address the issue of multiple testing, and using PCA allowed us to assess the relationship between air pollutants and CHDs in a multipollutant context.

Assigning exposure using ambient concentrations of pollutants at their residential location does not account for time spent indoors and pollutant concentrations at other relevant locations. This exposure misclassification could influence our effect estimates if there are differences in these factors between cases and controls—for example, if mothers of case offspring had more difficult pregnancies, limiting their outdoor movement. There is also the potential for exposure misclassification by assigning exposure using the nearest monitor. Previous research suggests that even when limiting to the closest monitor within 10 km, the 10th–90th percentile exposure contrast is larger for nearest monitor analyses than for other forms of exposure assessment (Marshall et al. 2008). This would have less of an impact on our study where we categorized exposure based on the distribution, rather than performing contrasts on a fixed-unit change in exposure. In simulation analyses of air pollution and incidence of cardiovascular events, Kim et al. (2009) found that hazard ratios derived using nearest-monitor exposures were more biased than those derived using exposures obtained from kriging, particularly as the monitoring network became sparse. These biases tended to be toward the null, suggesting that our estimates may underestimate the true relationship between air pollutants and CHDs.

The NBDPS had a response slightly lower than 70% and is subject to potential selection bias based on who agrees to participate. Additionally, there is the potential for selection bias if the factors that contribute to women living near a pollutant monitor are also associated with pollutant exposure and CHDs. We did not observe strong associations between maternal demographic factors that could influence residential location and the presence of CHDs within our full population. However, our results may not be generalizable to populations who live > 50 km from an air monitor. Because air pollutants vary spatially, study center may confound the relationship between air pollutants and CHDs through pathways such as differences in case ascertainment and resident sociodemographics. We controlled for a marker of case ascertainment in our model, but we may not have completely accounted for differences in case ascertainment across sites, and residual confounding due to unmeasured, spatially varying factors including other environmental exposures could affect our results. Our PCA analysis was based on a highly select population who live near multiple pollutant monitors and may not be generalizable to the larger population.

We conducted many analytic contrasts, and although hierarchical regression partially addresses multiple comparisons, it is possible that some of our findings are attributable to chance. We used hierarchical regression because other methods that deal with multiple comparisons do not account for the association between estimates that occurs when assessing weekly exposures simultaneously. It is possible that certain subgroups in the population may be more vulnerable to the impacts of air pollution due to their diet, genetics, co-exposures, or other factors not addressed in this study. If this is the case, we may have underestimated or missed an association between air pollutants and CHDs that would be seen only in that select population.

In this study, we observed increased odds of several CHDs with greater pollutant exposure. Some of these positive associations were observed only during specific weeks within the window of cardiac development, suggesting that accounting for temporal variability in pollutant concentrations and developmental susceptibility can improve effect estimation. Future research should focus on further exploration of temporal windows of susceptibility and examining the risk of CHDs within a multipollutant context, in order to gain understanding of the contribution of the different air pollutants.

## Supplemental Material

(845 KB) PDF
