# Supplementary material for: Maternal Exposure to Criteria Air Pollutants and Congenital Heart Defects in Offspring: Results from the National Birth Defects Prevention Study
Source: Environ Health Perspect. 2014 Apr 11;122(8):863–72. doi: 10.1289/ehp.1307289 (PMC4123026; doi:10.1289/ehp.1307289)
Supplement: (845 KB) PDF [file ehp.1307289.s001.pdf]

*Environmental Health Perspectives* doi:10.1289/ehp.1307289

# Supplemental Material

# Maternal Exposure to Criteria Air Pollutants and Congenital Heart Defects in Offspring: Results from the National Birth Defects Prevention Study

Jeanette A. Stingone, Thomas J. Luben, Julie L. Daniels, Montserrat Fuentes, David B. Richardson, Arthur S. Aylsworth, Amy H. Herring, Marlene Anderka, Lorenzo Botto, Adolfo Correa, Suzanne M. Gilboa, Peter H. Langlois, Bridget Mosley, Gary M. Shaw, Csaba Siffel, Andrew F. Olshan, and the National Birth Defects Prevention Study

**Table of Contents** **Page**

**Table S1:** Ratios used to control for differences in case ascertainment, National Birth Defects Prevention Study, 1997-2006. 1

**Table S2:** Adjusted odds ratios and 95% confidence intervals between congenital heart defects and 7-week average exposure to criteria air pollutants, National Birth Defects Prevention Study, 1997-2006. 2

**Table S3:** Adjusted odds ratios and 95% confidence intervals between congenital heart defects and 7-week exposure to nitrogen dioxide and particulate matter by distance to major road (≤50 km or >50 km), National Birth Defects Prevention Study, 1997-2006. 7

**Table S4:** Adjusted odds ratios and 95% confidence intervals between congenital heart defects and weekly exposure to criteria air pollutants from hierarchical models, National Birth Defects Prevention Study, 1997-2006.[a] 9

**Table S5:** Adjusted odds ratios and 95% confidence intervals between congenital heart defects and pollutant factors identified through principal components analysis, National Birth Defects Prevention Study, 1999-2006. 30

**Table S6:** Adjusted[a] odds ratios and 95% confidence intervals between congenital heart defects and 7-week average exposure to criteria air pollutants among participants who lived within 10 km of a stationary air monitor, National Birth Defects Prevention Study, 1997-2006. 31

**Table S1:** Ratios used to control for differences in case ascertainment, National Birth Defects Prevention Study, 1997-2006.

| Study Center | Ratio of Septal Cases to Total Congenital Heart Defect Cases[a] |
|---|---|
| Arkansas | 0.495 |
| California | 0.233 |
| Iowa | 0.359 |
| Massachusetts | 0.341 |
| Metro Atlanta | 0.400 |
| New York | 0.306 |
| North Carolina | 0.358 |
| Texas | 0.601 |
| Utah | 0.307 |

[a]Ratios calculated using the population of all simple, isolated congenital heart cases within the National Birth Defects Prevention Study from 1997-2006.

**Table S2:** Adjusted odds ratios and 95% confidence intervals between congenital heart defects and 7-week average exposure to criteria air pollutants, National Birth Defects Prevention Study, 1997-2006.[a]

| Defects | <10th centile | 10th centile-<50th centile | 50th centile-<90th centile | ≥90th centile |
|---|---|---|---|---|
| **Carbon Monoxide** | | | | |
| LVOTO[b] | 1.00 | 1.11 (0.80,1.53) | 1.11 (0.80,1.55) | 0.95 (0.62,1.45) |
| Aortic stenosis[c] | 1.00 | 0.93 (0.49,1.79) | 0.94 (0.49,1.81) | 0.76 (0.32,1.79) |
| COA[c] | 1.00 | 1.13 (0.7,1.82) | 0.93 (0.57,1.52) | 0.98 (0.53,1.82) |
| HLHS[c] | 1.00 | 1.22 (0.71,2.12) | 1.50 (0.87,2.60) | 1.07 (0.53,2.14) |
| Conotruncal[b] | 1.00 | 1.21 (0.90,1.64) | 1.28 (0.94,1.73) | 1.22 (0.84,1.79) |
| d-TGA[c] | 1.00 | 1.34 (0.81,2.22) | 1.34 (0.80,2.23) | 1.15 (0.61,2.19) |
| TOF[c] | 1.00 | 1.22 (0.81,1.83) | 1.35 (0.90,2.03) | 1.29 (0.78,2.14) |
| Other conotruncals[c,d] | 1.00 | 0.96 (0.48,1.95) | 0.92 (0.45,1.88) | 1.08 (0.46,2.56) |
| Common truncus[e] | 1.00 | 1.03 (0.35,3.99) | 0.70 (0.22,2.82) | 0.33 (0.03,2.06) |
| DORV-TGA /other[e] | 1.00 | 1.03 (0.30,5.35) | 0.88 (0.25,4.60) | 1.34 (0.29,7.82) |
| IAA TypeB/NOS[e] | 1.00 | 0.34 (0.07,2.03) | 0.17 (0.02,1.27) | 1.72 (0.32,10.6) |
| VSDconoventricular[e] | 1.00 | 0.80 (0.26,3.18) | 1.33 (0.45,5.15) | 1.32 (0.28,6.33) |
| APVR[b] | 1.00 | 0.46 (0.25,0.84) | 0.48 (0.26,0.88) | 0.59 (0.27,1.28) |
| TAPVR[c] | 1.00 | 0.53 (0.28,1.00) | 0.43 (0.22,0.84) | 0.67 (0.30,1.50) |
| AVSD[b] | 1.00 | 0.95 (0.35,2.56) | 1.11 (0.41,2.98) | 0.75 (0.20,2.83) |
| RVOTO[b] | 1.00 | 0.94 (0.67,1.33) | 0.97 (0.69,1.37) | 0.89 (0.57,1.39) |
| Pulmonary/tricuspid atresia[c] | 1.00 | 0.77 (0.40,1.50) | 0.75 (0.38,1.47) | 0.72 (0.30,1.73) |
| Pulmonary atresia[e] | 1.00 | 0.68 (0.33,1.53) | 0.55 (0.26,1.27) | 0.68 (0.24,1.87) |
| Tricuspid atresia[e] | 1.00 | 0.81 (0.26,3.25) | 1.12 (0.38,4.4) | 0.79 (0.13,4.11) |
| PVS[c] | 1.00 | 0.92 (0.61,1.37) | 1.00 (0.67,1.49) | 0.92 (0.56,1.53) |
| Ebstein's Anomaly[e] | 1.00 | 8.5 (1.15,1081) | 5.25 (0.67,678) | 4.40 (0.35,612) |
| Septal[b] | 1.00 | 1.01 (0.78,1.33) | 1.13 (0.86,1.47) | 1.30 (0.95,1.80) |
| $VSD_{pm}$[c] | 1.00 | 0.87 (0.62,1.23) | 1.04 (0.74,1.47) | 1.01 (0.66,1.56) |
| $VSD_{muscular}$[e] | 1.00 | 2.13 (0.22,272) | 2.37 (0.27,297) | 2.78 (0.30,354) |
| ASD[c] | 1.00 | 1.08 (0.74,1.59) | 0.99 (0.67,1.46) | 1.13 (0.71,1.80) |
| **Nitrogen Dioxide** | | | | |
| LVOTO[b] | 1.00 | 1.44 (1.00,2.08) | 1.49 (1.03,2.15) | 1.53 (0.98,2.39) |
| Aortic stenosis[c] | 1.00 | 2.22 (0.94,5.26) | 1.66 (0.69,3.99) | 2.22 (0.83,5.97) |
| COA[c] | 1.00 | 1.74 (0.91,3.32) | 2.34 (1.24,4.42) | 2.50 (1.21,5.18) |
| HLHS[c] | 1.00 | 1.07 (0.65,1.78) | 1.04 (0.62,1.72) | 0.85 (0.43,1.68) |
| Conotruncal[b] | 1.00 | 1.36 (0.99,1.88) | 1.32 (0.96,1.82) | 1.42 (0.96,2.11) |
| d-TGA[c] | 1.00 | 1.18 (0.71,1.95) | 1.24 (0.75,2.04) | 1.29 (0.69,2.38) |
| TOF[c] | 1.00 | 1.51 (0.98,2.34) | 1.27 (0.82,1.97) | 1.51 (0.89,2.57) |
| Other conotruncals[c,d] | 1.00 | 1.33 (0.55,3.22) | 1.81 (0.76,4.31) | 1.49 (0.52,4.24) |
| Common truncus[e] | 1.00 | 5.60 (0.70,724) | 9.65 (1.29,1233) | 3.46 (0.18,507) |
| DORV-TGA /other[e] | 1.00 | 1.10 (0.24,10.4) | 1.57 (0.37,14.6) | 1.25 (0.16,13.9) |
| IAA TypeB/NOS[e] | 1.00 | 0.68 (0.12,6.89) | 0.21 (0.02,2.61) | 1.36 (0.16,15.8) |

| Defects | <10th centile | 10th centile-<50th centile | 50th centile-<90th centile | ≥90th centile |
|---|---|---|---|---|
| VSDconoventricular[e] | 1.00 | 0.60 (0.21,2.05) | 0.76 (0.28,2.54) | 0.87 (0.19,3.65) |
| APVR[b] | 1.00 | 0.54 (0.28,1.03) | 0.57 (0.30,1.09) | 0.89 (0.41,1.94) |
| TAPVR[c] | 1.00 | 0.51 (0.26,1.01) | 0.54 (0.28,1.06) | 0.92 (0.42,2.03) |
| AVSD[b] | 1.00 | 0.63 (0.27,1.47) | 0.66 (0.29,1.54) | 0.64 (0.20,1.98) |
| RVOTO[b] | 1.00 | 1.32 (0.88,1.97) | 1.50 (1.01,2.24) | 2.22 (1.40,3.52) |
| Pulmonary/tricuspid atresia[c] | 1.00 | 2.45 (0.86,6.95) | 2.02 (0.71,5.79) | 2.33 (0.71,7.68) |
| Pulmonary atresia[e] | 1.00 | 1.76 (0.64,6.64) | 1.58 (0.57,5.96) | 2.10 (0.59,8.97) |
| Tricuspid atresia[e] | 1.00 | 2.81 (0.68,25.9) | 1.88 (0.43,17.7) | 2.07 (0.27,22.9) |
| PVS[c] | 1.00 | 1.02 (0.66,1.59) | 1.30 (0.85,2.00) | 2.03 (1.23,3.33) |
| Ebstein's Anomaly[e] | 1.00 | 6.17 (0.79,795) | 8.39 (1.12,1075) | 11.9 (1.25,1582) |
| Septal[b] | 1.00 | 1.24 (0.94,1.64) | 1.23 (0.94,1.63) | 1.44 (1.02,2.03) |
| $VSD_{pm}$[c] | 1.00 | 1.13 (0.78,1.64) | 1.18 (0.82,1.71) | 1.47 (0.94,2.30) |
| $VSD_{muscular}$[e] | 1.00 | 0.75 (0.17,3.70) | 0.56 (0.13,2.84) | 0.46 (0.08,2.85) |
| ASD[c] | 1.00 | 1.29 (0.87,1.91) | 1.25 (0.84,1.86) | 1.23 (0.74,2.04) |
| **Ozone[f]** | | | | |
| LVOTO[b] | 1.00 | 0.96 (0.74,1.25) | 0.95 (0.73,1.23) | 0.94 (0.73,1.22) |
| Aortic stenosis[c] | 1.00 | 1.21 (0.70,2.11) | 0.95 (0.53,1.67) | 1.07 (0.61,1.87) |
| COA[c] | 1.00 | 0.74 (0.49,1.11) | 0.94 (0.64,1.38) | 0.97 (0.67,1.42) |
| HLHS[c] | 1.00 | 1.07 (0.72,1.58) | 0.95 (0.64,1.41) | 0.86 (0.58,1.29) |
| Conotruncal[b] | 1.00 | 1.16 (0.92,1.45) | 0.97 (0.76,1.22) | 1.00 (0.79,1.26) |
| d-TGA[c] | 1.00 | 0.82 (0.56,1.21) | 1.08 (0.76,1.53) | 0.81 (0.56,1.19) |
| TOF[c] | 1.00 | 1.34 (0.99,1.81) | 0.86 (0.62,1.19) | 1.11 (0.82,1.51) |
| Other conotruncals[c,d] | 1.00 | 1.44 (0.79,2.62) | 1.07 (0.56,2.02) | 1.15 (0.62,2.13) |
| Common truncus[e] | 1.00 | 1.73 (0.48,7.32) | 1.64(0.43,7.12) | 2.40 (0.70, 9.98) |
| DORV-TGA /other[e] | 1.00 | 1.26 (0.41,4.09) | 1.34 (0.44,4.32) | 0.97 (0.31,3.23) |
| IAA TypeB/NOS[e] | 1.00 | 1.10 (0.23,5.25) | 0.15 (0.01,1.58) | 0.85 (0.14,4.40) |
| VSDconoventricular[e] | 1.00 | 1.53 (0.61,3.95) | 0.98 (0.35,2.67) | 0.89 (0.32,2.42) |
| APVR[b] | 1.00 | 0.95 (0.53,1.71) | 1.11 (0.63,1.94) | 0.95 (0.54,1.70) |
| TAPVR[c] | 1.00 | 1.22 (0.65,2.27) | 1.35 (0.74,2.46) | 1.09 (0.59,2.04) |
| AVSD[b] | 1.00 | 1.22 (0.53,2.81) | 1.18 (0.51,2.72) | 1.24 (0.54,2.85) |
| RVOTO[b] | 1.00 | 1.21 (0.91,1.61) | 1.07 (0.80,1.44) | 1.26 (0.95,1.67) |
| Pulmonary/tricuspid atresia[c] | 1.00 | 1.25 (0.67,2.32) | 0.92 (0.48,1.79) | 1.31 (0.72,2.39) |
| Pulmonary atresia[e] | 1.00 | 1.23 (0.55,2.78) | 1.06 (0.46,2.45) | 1.87 (0.91,4.01) |
| Tricuspid atresia[e] | 1.00 | 1.27 (0.49,3.34) | 0.71 (0.22,2.06) | 0.59 (0.19,1.72) |
| PVS[c] | 1.00 | 1.27 (0.91,1.78) | 1.17 (0.84,1.64) | 1.27 (0.91,1.77) |
| Ebstein's Anomaly[e] | 1.00 | 0.74 (0.29,1.85) | 0.68 (0.25,1.74) | 0.88 (0.34,2.22) |
| Septal[b] | 1.00 | 1.03 (0.85,1.25) | 0.94 (0.77,1.14) | 0.89 (0.72,1.09) |
| VSDpm[c] | 1.00 | 0.96 (0.73,1.26) | 0.93 (0.70,1.23) | 1.06 (0.81,1.39) |
| VSDmuscular[e] | 1.00 | 0.91 (0.36,2.19) | 1.28 (0.45,3.41) | 1.08 (0.47,2.42) |
| ASD[c] | 1.00 | 1.25 (0.97,1.61) | 1.10 (0.84,1.44) | 0.85 (0.63,1.14) |

| Defects | <10th centile | 10th centile-<50th centile | 50th centile-<90th centile | ≥90th centile |
|---|---|---|---|---|
| **$PM_{10}$** | | | | |
| LVOTO[b] | 1.00 | 1.10 (0.79,1.55) | 1.37 (0.99,1.91) | 1.12 (0.72,1.72) |
| Aortic stenosis[c] | 1.00 | 1.24 (0.62,2.46) | 1.51 (0.76,2.98) | 0.92 (0.36,2.32) |
| COA[c] | 1.00 | 1.71 (0.95,3.09) | 1.77 (0.98,3.20) | 1.68 (0.82,3.45) |
| HLHS[c] | 1.00 | 0.79 (0.48,1.31) | 1.24 (0.76,2.01) | 0.98 (0.52,1.87) |
| Conotruncal[b] | 1.00 | 1.21 (0.89,1.64) | 1.31 (0.96,1.78) | 1.44 (0.99,2.10) |
| d-TGA[c] | 1.00 | 1.10 (0.67,1.81) | 1.16 (0.71,1.92) | 1.45 (0.80,2.64) |
| TOF[c] | 1.00 | 1.14 (0.76,1.70) | 1.35 (0.91,2.02) | 1.30 (0.79,2.16) |
| Other conotruncals[c,d] | 1.00 | 2.12 (0.84,5.39) | 1.68 (0.65,4.34) | 2.07 (0.71,6.02) |
| Common truncus[e] | 1.00 | 2.79 (0.68,25.7) | 2.34 (0.55,21.8) | 1.75 (0.22,20.0) |
| DORV-TGA /other[e] | 1.00 | 1.21 (0.36,6.20) | 0.94 (0.27,4.93) | 1.41 (0.29,8.49) |
| IAA TypeB/NOS[e] | 1.00 | 2.58 (0.27,342) | 1.01 (0.05,147) | 8.75 (0.60,1250) |
| VSDconoventricular[e] | 1.00 | 1.29 (0.38,6.60) | 1.12 (0.32,5.82) | 1.25 (0.25,7.71) |
| APVR[b] | 1.00 | 1.49 (0.66,3.33) | 1.34 (0.59,3.03) | 1.44 (0.55,3.79) |
| TAPVR[c] | 1.00 | 1.28 (0.57,2.89) | 1.12 (0.49,2.55) | 1.36 (0.52,3.60) |
| AVSD[b] | 1.00 | 6.25 (0.84,46.26) | 6.70 (0.90,49.7) | 4.80 (0.53,43.4) |
| RVOTO[b] | 1.00 | 1.00 (0.71,1.41) | 1.18 (0.84,1.66) | 0.98 (0.62,1.55) |
| Pulmonary/tricuspid atresia[c] | 1.00 | 0.75 (0.38,1.49) | 0.91 (0.46,1.80) | 0.43 (0.16,1.21) |
| Pulmonary atresia[e] | 1.00 | 0.68 (0.32,1.59) | 0.82 (0.39,1.90) | 0.48 (0.13,1.51) |
| Tricuspid atresia[e] | 1.00 | 0.84 (0.28,3.32) | 1.04 (0.35,4.02) | 0.32 (0.03,2.07) |
| PVS[c] | 1.00 | 1.13 (0.75,1.70) | 1.31 (0.87,1.98) | 1.17 (0.68,2.00) |
| Ebstein's Anomaly[e] | 1.00 | 0.60 (0.21,2.01) | 0.73 (0.26,2.43) | 1.26 (0.31,5.07) |
| Septal[b] | 1.00 | 1.23 (0.97,1.57) | 1.21 (0.95,1.55) | 0.91 (0.65,1.28) |
| $VSD_{pm}$[c] | 1.00 | 1.42 (0.98,2.05) | 1.42 (0.98,2.05) | 1.18 (0.73,1.92) |
| $VSD_{muscular}$[e] | 1.00 | 1.18 (0.52,2.88) | 1.68 (0.73,4.13) | 0.69 (0.12,3.06) |
| ASD[c] | 1.00 | 1.14 (0.83,1.57) | 1.09 (0.79,1.51) | 0.81 (0.51,1.28) |
| **$PM_{2.5}$** | | | | |
| LVOTO[b] | 1.00 | 1.03 (0.76,1.39) | 0.85 (0.62,1.15) | 1.25 (0.86,1.82) |
| Aortic stenosis[c] | 1.00 | 0.90 (0.53,1.51) | 0.59 (0.33,1.03) | 0.96 (0.47,1.94) |
| COA[c] | 1.00 | 0.88 (0.56,1.37) | 0.85 (0.54,1.35) | 1.06 (0.60,1.87) |
| HLHS[c] | 1.00 | 1.59 (0.91,2.79) | 1.25 (0.70,2.21) | 2.04 (1.07,3.89) |
| Conotruncal[b] | 1.00 | 0.97 (0.73,1.29) | 0.98 (0.73,1.31) | 1.20 (0.84,1.72) |
| d-TGA[c] | 1.00 | 0.96 (0.60,1.53) | 1.03 (0.65,1.65) | 1.07 (0.59,1.93) |
| TOF[c] | 1.00 | 0.97 (0.66,1.44) | 1.02 (0.69,1.51) | 1.32 (0.83,2.12) |
| Other conotruncals[c,d] | 1.00 | 1.02 (0.50,2.05) | 0.75 (0.36,1.57) | 1.05 (0.45,2.49) |
| Common truncus[e] | 1.00 | 1.20 (0.35,6.16) | 0.97 (0.27,5.13) | 2.54 (0.58,14.6) |
| DORV-TGA /other[e] | 1.00 | 0.54 (0.20,1.64) | 0.40 (0.13,1.31) | 0.22 (0.02,1.22) |
| IAA TypeB/NOS[e] | 1.00 | 0.56 (0.09,5.69) | 0.32 (0.04,3.53) | 2.35 (0.36,25.1) |
| VSDconoventricular[e] | 1.00 | 1.52 (0.47,7.70) | 1.03 (0.30,5.35) | 0.97 (0.18,6.00) |
| APVR[b] | 1.00 | 0.91 (0.48,1.71) | 0.68 (0.35,1.32) | 1.10 (0.50,2.44) |
| TAPVR[c] | 1.00 | 0.95 (0.48,1.87) | 0.68 (0.33,1.39) | 1.04 (0.44,2.44) |
| AVSD[b] | 1.00 | 2.36 (0.71,7.85) | 2.27 (0.68,7.59) | 2.67 (0.67,10.5) |

| Defects | <10th centile | 10th centile-<50th centile | 50th centile-<90th centile | ≥90th centile |
|---|---|---|---|---|
| RVOTO[b] | 1.00 | 0.92 (0.67,1.27) | 0.96 (0.69,1.32) | 0.93 (0.60,1.42) |
| Pulmonary/tricuspid atresia[c] | 1.00 | 0.89 (0.46,1.7) | 0.62 (0.31,1.23) | 0.74 (0.32,1.73) |
| Pulmonary atresia[e] | 1.00 | 0.68 (0.33,1.52) | 0.61 (0.29,1.40) | 0.69 (0.25,1.86) |
| Tricuspid atresia[e] | 1.00 | 1.37 (0.49,5.21) | 0.53 (0.15,2.27) | 0.90 (0.18,4.45) |
| PVS[c] | 1.00 | 0.93 (0.64,1.36) | 1.09 (0.74,1.58) | 1.05 (0.64,1.72) |
| Ebstein's Anomaly[e] | 1.00 | 0.80 (0.32,2.36) | 0.70 (0.27,2.13) | 0.45 (0.04,2.34) |
| Septal[b] | 1.00 | 0.89 (0.72,1.1) | 0.66 (0.53,0.83) | 0.62 (0.45,0.85) |
| $VSD_{pm}$[c] | 1.00 | 1.17 (0.83,1.64) | 1.06 (0.75,1.50) | 0.85 (0.54,1.35) |
| $VSD_{muscular}$[g] | 1.00 | n/a | n/a | n/a |
| ASD[c] | 1.00 | 0.80 (0.63,1.03) | 0.50 (0.38,0.65) | 0.54 (0.35,0.81) |
| **Sulfur Dioxide** | | | | |
| LVOTO[b] | 1.00 | 1.32 (0.88,1.98) | 1.49 (0.99,2.24) | 1.07 (0.64,1.79) |
| Aortic stenosis[c] | 1.00 | 0.93 (0.44,1.97) | 0.84 (0.39,1.83) | 0.58 (0.20,1.65) |
| COA[c] | 1.00 | 1.21 (0.65,2.24) | 1.74 (0.95,3.20) | 1.62 (0.79,3.30) |
| HLHS[c] | 1.00 | 1.98 (0.97,4.04) | 1.84 (0.89,3.78) | 0.96 (0.37,2.47) |
| Conotruncal[b] | 1.00 | 1.12 (0.78,1.60) | 1.35 (0.95,1.92) | 1.22 (0.79,1.88) |
| d-TGA[c] | 1.00 | 1.32 (0.72,2.43) | 1.61 (0.88,2.96) | 1.46 (0.71,2.98) |
| TOF[c] | 1.00 | 1.08 (0.67,1.73) | 1.20 (0.74,1.92) | 1.17 (0.66,2.07) |
| Other conotruncals[c,d] | 1.00 | 0.90 (0.37,2.16) | 1.50 (0.64,3.53) | 0.91 (0.30,2.79) |
| Common truncus[e] | 1.00 | 0.74 (0.21,3.17) | 1.19 (0.35,5.14) | 1.58 (0.31,8.16) |
| DORV-TGA /other[e] | 1.00 | 0.79 (0.18,4.66) | 1.71 (0.42,10.1) | 0.41 (0.00,5.48) |
| IAA TypeB/NOS[h] | 1.00 | n/a | n/a | n/a |
| VSDconoventricular[e] | 1.00 | 0.82 (0.22,4.40) | 1.27 (0.35,6.91) | 0.50 (0.04,3.88) |
| APVR[b] | 1.00 | 1.33 (0.54,3.30) | 1.65 (0.67,4.09) | 1.02 (0.32,3.28) |
| TAPVR[c] | 1.00 | 1.03 (0.41,2.61) | 1.46 (0.58,3.62) | 0.93 (0.29,2.99) |
| AVSD[b] | 1.00 | 0.82 (0.28,3.20) | 1.10 (0.39,4.23) | 1.54 (0.45,6.46) |
| RVOTO[b] | 1.00 | 1.81 (1.15,2.83) | 1.65 (1.04,2.60) | 1.24 (0.70,2.18) |
| Pulmonary/tricuspid atresia[c] | 1.00 | 1.13 (0.49,2.61) | 1.17 (0.50,2.73) | 0.76 (0.25,2.31) |
| Pulmonary atresia[e] | 1.00 | 1.22 (0.47,3.94) | 1.31 (0.50,4.23) | 0.74 (0.16,3.09) |
| Tricuspid atresia[e] | 1.00 | 0.81 (0.25,3.34) | 0.66 (0.19,2.82) | 0.78 (0.13,4.17) |
| PVS[c] | 1.00 | 2.34 (1.33,4.14) | 2.06 (1.16,3.67) | 1.48 (0.74,2.97) |
| Ebstein's Anomaly[e] | 1.00 | 0.75 (0.24,3.01) | 0.76 (0.23,3.15) | 1.45 (0.34,6.72) |
| Septal[b] | 1.00 | 1.06 (0.82,1.38) | 1.09 (0.84,1.43) | 1.12 (0.80,1.58) |
| $VSD_{pm}$[c] | 1.00 | 1.26 (0.84,1.89) | 1.36 (0.90,2.05) | 1.48 (0.91,2.42) |
| $VSD_{muscular}$[h] | 1.00 | n/a | n/a | n/a |
| ASD[c] | 1.00 | 0.93 (0.68,1.28) | 0.83 (0.59,1.16) | 0.67 (0.41,1.09) |

Abbreviations: APVR-anomalous pulmonary venous return; ASD-atrial septal defect; AVSD-atrioventricular septal defect; COA-coarctation of the aorta; DORV-TGA/Other-double outlet right ventricle with transposition of the great arteries or not (other); d-TGA-d-transposition of the great arteries;

HLHS-hypoplastic left heart syndrome; IAA TypeB/NOS-interrupted aortic arch Type B or not otherwise specified; LVOTO-left ventricular outflow tract obstructions; $PM_{10}$-particulate matter less than 10 microns in diameter; $PM_{2.5}$-particulate matter less than 2.5 microns in diameter; PVS-pulmonary valve stenosis; RVOTO-right ventricular outflow tract obstructions; TAPVR-total anomalous pulmonary venous return; TOF-tetralogy of Fallot; VSDconoventricular- conoventricular septal defects; $VSD_{muscular}$-muscular ventricular septal defects; $VSD_{pm}$-perimembranous ventricular septal defects.

[a]All results for the National Birth Defects Prevention Study population from 1997-2006, except for $PM_{2.5}$ which was 1999-2006 due to unavailability of monitoring data for $PM_{2.5}$ prior to 1999. [b]Estimates from a hierarchical regression model. First stage was polytomous logistic model with defect groupings and adjusted for maternal race, age, educational attainment, household income, maternal smoking status and alcohol consumption during early pregnancy, nativity, and site-specific heart defect ratio. Second stage was a linear model with indicator variables for defect grouping and level of exposure. Defect-groupings include all individual defects listed underneath with the following additions: LVOTO also includes IAA-Type A and APVR also includes partial APVR. Those defects could not be analyzed individually due to limited sample size. [c]Estimates result from a hierarchical regression model, same as above but used individual defects as outcomes in first-stage model and included indicator variable for individual defect in second-stage model. [d]Other conotruncals includes common truncus, interrupted aortic-arch, type B and type not specified, double outlet right ventricle defects, and conoventricular septal defects. [e]Estimates result from model utilizing Firth's penalized maximum likelihood regression to deal with quasi-separation of points due to small sample size in certain cells. Model adjusted for same variables as above. [f]For ozone, the three categories of exposure were $25^{th}$ to less than the $50^{th}$ centile, $50^{th}$ centile to less than the $75^{th}$ centile, at or greater than the $75^{th}$ centile, with the referent grouping being below the $25^{th}$ centile. [g]$VSD_{muscular}$ defects were only collected in the first year of data collection, 1997. There was no available monitoring data for $PM_{2.5}$ during this time. [h]Adjusted odds ratios could not be estimated due to very small number of cases.

**Table S3:** Adjusted odds ratios and 95% confidence intervals between congenital heart defects and 7-week exposure to nitrogen dioxide and particulate matter by distance to major road (≤50 km or >50km), National Birth Defects Prevention Study, 1997-2006.

| Defect | ≤50 km: <10th centile | ≤50 km: 10th centile-<50th centile | ≤50 km: 50th centile-<90th centile | ≤50 km: ≥90th centile | >50 km: <10th centile | >50 km: 10th centile-<50th centile | >50 km: 50th centile-<90th centile | >50 km: ≥90th centile |
|---|---|---|---|---|---|---|---|---|
| **Nitrogen Dioxide** | | | | | | | | |
| LVOTO[a] | 1.00 | 1.01 (0.45,2.26) | 1.31 (0.60,2.85) | 2.11 (0.82,5.45) | 1.00 | 1.54 (1.01,2.33) | 1.53 (1.01,2.33) | 1.41 (0.85,2.34) |
| Aortic stenosis[b] | 1.00 | 3.29 (0.40,26.9) | 1.42 (0.16,13.0) | 7.52 (0.81,69.9) | 1.00 | 2.03 (0.79,5.25) | 1.63 (0.63,4.25) | 1.57 (0.50,4.93) |
| COA[b] | 1.00 | 0.23 (0.05,0.98) | 1.22 (0.44,3.40) | 1.69 (0.47,6.16) | 1.00 | 2.79 (1.19,6.52) | 3.28 (1.41,7.61) | 3.32 (1.30,8.43) |
| HLHS[b] | 1.00 | 1.60 (0.44,5.78) | 1.41 (0.39,5.14) | 1.12 (0.18,6.97) | 1.00 | 0.97 (0.56,1.70) | 0.95 (0.55,1.66) | 0.76 (0.36,1.60) |
| Conotruncal[a] | 1.00 | 3.17 (1.21,8.26) | 3.80 (1.47,9.80) | 7.12 (2.53,20.0) | 1.00 | 1.16 (0.82,1.64) | 1.07 (0.76,1.52) | 0.96 (0.62,1.50) |
| d-TGA[b] | 1.00 | 2.68 (0.59,12.0) | 3.54 (0.81,15.6) | 6.23 (1.24,31.2) | 1.00 | 1.00 (0.58,1.71) | 1.00 (0.58,1.71) | 0.88 (0.43,1.77) |
| TOF[b] | 1.00 | 4.66 (1.08,20.1) | 4.53 (1.05,19.5) | 11.1 (2.39,51.0) | 1.00 | 1.26 (0.79,1.99) | 1.01 (0.63,1.61) | 0.92 (0.50,1.69) |
| Other conotruncals[b,c] | 1.00 | 1.34 (0.15,12.2) | 3.06 (0.38,24.6) | 1.49 (0.09,24.5) | 1.00 | 1.28 (0.49,3.38) | 1.60 (0.62,4.17) | 1.40 (0.43,4.50) |
| APVR[a] | 1.00 | 1.01 (0.26,3.92) | 1.18 (0.31,4.42) | 1.10 (0.18,6.85) | 1.00 | 0.45 (0.21,0.95) | 0.45 (0.21,0.95) | 0.88 (0.36,2.14) |
| RVOTO[a] | 1.00 | 1.56 (0.62,3.95) | 1.14 (0.44,2.97) | 3.55 (1.25,10.1) | 1.00 | 1.25 (0.80,1.95) | 1.53 (0.99,2.38) | 1.98 (1.19,3.31) |
| Pulmonary/tricuspid atresia[b] | 1.00 | 1.40 (0.28,6.92) | 0.96 (0.18,5.10) | 1.83 (0.25,13.4) | 1.00 | 3.6 (0.85,15.3) | 3.04 (0.71,13.0) | 3.07 (0.61,15.5) |
| PVS[b] | 1.00 | 1.48 (0.48,4.58) | 1.09 (0.34,3.47) | 3.11 (0.86,11.2) | 1.00 | 0.93 (0.58,1.50) | 1.28 (0.80,2.04) | 1.85 (1.08,3.18) |
| Septal[a] | 1.00 | 1.74 (0.93,3.26) | 1.41 (0.74,2.66) | 1.80 (0.80,4.03) | 1.00 | 1.14 (0.84,1.55) | 1.18 (0.87,1.61) | 1.35 (0.93,1.98) |
| $VSD_{pm}$[b] | 1.00 | 2.68 (1.02,7.05) | 1.98 (0.74,5.27) | 2.87 (0.91,9.06) | 1.00 | 0.93 (0.62,1.40) | 1.05 (0.71,1.57) | 1.28 (0.78,2.09) |
| ASD[b] | 1.00 | 1.07 (0.48,2.38) | 0.96 (0.43,2.17) | 0.97 (0.31,3.05) | 1.00 | 1.36 (0.87,2.14) | 1.34 (0.85,2.11) | 1.30 (0.73,2.30) |
| **$PM_{10}$** | | | | | | | | |
| LVOTO[a] | 1.00 | 1.21 (0.56,2.60) | 1.33 (0.62,2.86) | 2.20 (0.85,5.71) | 1.00 | 1.08 (0.74,1.57) | 1.37 (0.95,1.98) | 0.96 (0.59,1.57) |
| Aortic stenosis[b] | 1.00 | 1.49 (0.32,6.98) | 1.15 (0.23,5.69) | 3.90 (0.68,22.5) | 1.00 | 1.18 (0.54,2.55) | 1.57 (0.73,3.35) | 0.49 (0.14,1.67) |
| COA[b] | 1.00 | 0.56 (0.16,1.90) | 1.34 (0.44,4.09) | 1.76 (0.42,7.42) | 1.00 | 2.18 (1.08,4.39) | 1.97 (0.97,3.98) | 1.78 (0.76,4.12) |
| HLHS[b] | 1.00 | 1.89 (0.55,6.50) | 1.43 (0.40,5.11) | 1.76 (0.34,9.13) | 1.00 | 0.63 (0.36,1.09) | 1.19 (0.71,2.01) | 0.88 (0.44,1.78) |
| Conotruncal[a] | 1.00 | 1.19 (0.59,2.40) | 1.55 (0.78,3.09) | 2.63 (1.14,6.06) | 1.00 | 1.20 (0.85,1.69) | 1.25 (0.89,1.76) | 1.27 (0.84,1.94) |
| d-TGA[b] | 1.00 | 0.97 (0.31,3.06) | 1.46 (0.48,4.44) | 2.04 (0.52,8.00) | 1.00 | 1.12 (0.64,1.94) | 1.10 (0.63,1.92) | 1.38 (0.71,2.68) |
| TOF[b] | 1.00 | 1.14 (0.45,2.89) | 1.61 (0.65,4.00) | 2.86 (0.98,8.42) | 1.00 | 1.13 (0.72,1.77) | 1.30 (0.83,2.04) | 1.08 (0.61,1.92) |
| Other conotruncals[b,c] | 1.00 | 2.34 (0.29,19.0) | 1.54 (0.18,13.4) | 3.13 (0.27,35.9) | 1.00 | 2.07 (0.73,5.88) | 1.67 (0.58,4.81) | 1.96 (0.59,6.50) |

| Defect | ≤50 km: <10th centile | ≤50 km: 10th centile-<50th centile | ≤50 km: 50th centile-<90th centile | ≤50 km: ≥90th centile | >50 km: <10th centile | >50 km: 10th centile-<50th centile | >50 km: 50th centile-<90th centile | >50 km: ≥90th centile |
|---|---|---|---|---|---|---|---|---|
| APVR[d] | | | | | | | | |
| RVOTO[a] | 1.00 | 1.74 (0.66,4.63) | 1.99 (0.75,5.28) | 3.59 (1.14,11.4) | 1.00 | 0.90 (0.62,1.3) | 1.07 (0.74,1.54) | 0.75 (0.45,1.25) |
| Pulmonary/tricuspid atresia[b] | 1.00 | 2.72 (0.34,21.8) | 2.49 (0.31,20.3) | 1.49 (0.09,24.6) | 1.00 | 0.58 (0.27,1.22) | 0.77 (0.37,1.58) | 0.29 (0.09,0.96) |
| PVS[b] | 1.00 | 1.29 (0.43,3.93) | 1.71 (0.57,5.12) | 3.25 (0.87,12.2) | 1.00 | 1.09 (0.70,1.69) | 1.24 (0.80,1.93) | 0.95 (0.52,1.72) |
| Septal[a] | 1.00 | 1.51 (0.86,2.64) | 1.43 (0.81,2.52) | 1.36 (0.60,3.10) | 1.00 | 1.17 (0.89,1.53) | 1.16 (0.88,1.52) | 0.83 (0.57,1.21) |
| $VSD_{pm}$[b] | 1.00 | 1.68 (0.73,3.89) | 1.59 (0.68,3.70) | 1.77 (0.56,5.57) | 1.00 | 1.36 (0.90,2.04) | 1.38 (0.91,2.08) | 1.08 (0.63,1.86) |
| ASD[b] | 1.00 | 1.37 (0.66,2.83) | 1.24 (0.59,2.61) | 0.96 (0.30,3.06) | 1.00 | 1.10 (0.77,1.56) | 1.06 (0.74,1.51) | 0.77 (0.47,1.28) |

Abbreviations: APVR-anomalous pulmonary venous return; ASD-atrial septal defect; AVSD- atrioventricular septal defect; COA-coarctation of the aorta; d-TGA-d-transposition of the great arteries; HLHS-hypoplastic left heart syndrome; LVOTO-left ventricular outflow tract obstructions; $PM_{10}$-particulate matter less than 10 microns in diameter; PVS-pulmonary valve stenosis; RVOTO-right ventricular outflow tract obstructions; TOF-tetralogy of Fallot; $VSD_{pm}$-perimembranous ventricular septal defects.

[a]Estimates result from maximum-likelihood, polytomous logistic model between defect grouping and exposure which includes an interaction term between exposure and distance to roadway and adjusted for maternal race, maternal age, maternal educational attainment, maternal household income, maternal smoking status and alcohol consumption during early pregnancy, nativity, and site-specific heart defect ratio. All defect-groupings include the individual defects underneath with the following additions: LVOTO includes interrupted aortic arch-type A; APVR includes total and partial APVR; RVOTO includes Ebstein's anomaly; Septals includes muscular ventricular septal defects ($VSD_{muscular}$). [b]Estimates result from maximum-likelihood, polytomous logistic model between individual defect and exposure which includes an interaction term between exposure and distance to roadway and adjusted for maternal race, maternal age, maternal educational attainment, maternal household income, maternal smoking status and alcohol consumption during early pregnancy, nativity, and site-specific heart defect ratio. [c]Other conotruncals include common truncus, interrupted aortic-arch, type B and type not specified, double outlet right ventricle defects, and conoventricular septal defects. [d]Could not be estimated due to small sample size.

**Table S4:** Adjusted odds ratios and 95% confidence intervals between congenital heart defects and weekly exposure to criteria air pollutants from hierarchical models, National Birth Defects Prevention Study, 1997-2006.[a]

| Exposure and Defect | <10th centile | 10-<50th centile | 50-<90th centile | ≥90th centile |
|---|---|---|---|---|
| **CO (ppm)** | | | | |
| Week 2 | <0.52 | 0.52-<1.14 | 1.14-<2.21 | ≥2.21 |
| Week 3 | <0.52 | 0.52-<1.14 | 1.14-<2.21 | ≥2.21 |
| Week 4 | <0.52 | 0.52-<1.13 | 1.13-<2.24 | ≥2.24 |
| Week 5 | <0.51 | 0.52-<1.13 | 1.13-<2.21 | ≥2.21 |
| Week 6 | <0.52 | 0.52-<1.13 | 1.13-<2.23 | ≥2.23 |
| Week 7 | <0.51 | 0.51-<1.13 | 1.13-<2.19 | ≥2.19 |
| Week 8 | <0.52 | 0.52-<1.13 | 1.13-<2.23 | ≥2.23 |
| LVOTO[b] [OR (95% CI)] | | | | |
| Week 2 | 1.00 | 0.99 (0.67,1.47) | 0.99 (0.63,1.55) | 0.98 (0.56,1.70) |
| Week 3 | 1.00 | 1.06 (0.69,1.62) | 1.21 (0.75,1.95) | 1.15 (0.64,2.08) |
| Week 4 | 1.00 | 0.76 (0.51,1.13) | 0.60 (0.38,0.95) | 0.76 (0.43,1.34) |
| Week 5 | 1.00 | 1.13 (0.75,1.73) | 1.07 (0.67,1.72) | 0.79 (0.43,1.44) |
| Week 6 | 1.00 | 1.16 (0.76,1.76) | 1.28 (0.80,2.05) | 0.88 (0.48,1.60) |
| Week 7 | 1.00 | 1.03 (0.69,1.56) | 1.25 (0.79,1.99) | 1.45 (0.82,2.59) |
| Week 8 | 1.00 | 1.13 (0.76,1.69) | 1.09 (0.70,1.71) | 0.91 (0.52,1.62) |
| Aortic stenosis[c] | | | | |
| Week 2 | 1.00 | 0.88 (0.46,1.68) | 0.95 (0.46,1.93) | 0.90 (0.37,2.18) |
| Week 3 | 1.00 | 0.93 (0.47,1.82) | 1.06 (0.51,2.20) | 0.66 (0.25,1.74) |
| Week 4 | 1.00 | 0.65 (0.34,1.22) | 0.59 (0.29,1.20) | 0.68 (0.27,1.70) |
| Week 5 | 1.00 | 1.48 (0.73,2.99) | 1.38 (0.64,2.96) | 0.95 (0.36,2.50) |
| Week 6 | 1.00 | 1.14 (0.58,2.24) | 1.18 (0.57,2.47) | 0.74 (0.28,1.94) |
| Week 7 | 1.00 | 0.77 (0.40,1.50) | 1.11 (0.54,2.28) | 1.35 (0.55,3.29) |
| Week 8 | 1.00 | 0.93 (0.48,1.79) | 0.94 (0.46,1.92) | 0.90 (0.37,2.18) |
| COA[c] | | | | |
| Week 2 | 1.00 | 0.90 (0.54,1.52) | 0.93 (0.52,1.65) | 0.83 (0.40,1.74) |
| Week 3 | 1.00 | 1.2 (0.68,2.12) | 1.21 (0.65,2.27) | 1.11 (0.50,2.42) |
| Week 4 | 1.00 | 0.81 (0.48,1.37) | 0.62 (0.34,1.12) | 0.91 (0.43,1.90) |
| Week 5 | 1.00 | 0.94 (0.55,1.62) | 0.88 (0.48,1.62) | 0.78 (0.35,1.71) |
| Week 6 | 1.00 | 1.48 (0.83,2.64) | 1.39 (0.74,2.61) | 1.19 (0.54,2.62) |
| Week 7 | 1.00 | 1.44 (0.81,2.58) | 1.60 (0.85,3.01) | 1.70 (0.78,3.71) |
| Week 8 | 1.00 | 1.13 (0.65,1.94) | 1.12 (0.62,2.03) | 1.06 (0.50,2.25) |
| HLHS[c] | | | | |
| Week 2 | 1.00 | 1.13 (0.64,1.99) | 1.01 (0.54,1.89) | 1.01 (0.48,2.15) |
| Week 3 | 1.00 | 0.94 (0.52,1.70) | 1.13 (0.59,2.14) | 1.27 (0.58,2.76) |
| Week 4 | 1.00 | 1.12 (0.63,2.00) | 0.91 (0.48,1.72) | 1.05 (0.49,2.27) |
| Week 5 | 1.00 | 1.09 (0.60,1.97) | 1.13 (0.59,2.15) | 0.79 (0.35,1.78) |
| Week 6 | 1.00 | 0.97 (0.55,1.71) | 1.30 (0.70,2.43) | 0.82 (0.36,1.83) |

| Exposure and Defect | <10th centile | 10-<50th centile | 50-<90th centile | ≥90th centile |
|---|---|---|---|---|
| Week 7 | 1.00 | 0.79 (0.45,1.37) | 0.96 (0.52,1.77) | 1.26 (0.59,2.67) |
| Week 8 | 1.00 | 1.11 (0.63,1.93) | 0.98 (0.53,1.81) | 0.60 (0.27,1.33) |
| Conotruncal[b] | | | | |
| Week 2 | 1.00 | 1.17 (0.81,1.69) | 1.04 (0.69,1.57) | 0.93 (0.56,1.56) |
| Week 3 | 1.00 | 0.93 (0.63,1.36) | 0.95 (0.62,1.47) | 0.75 (0.43,1.29) |
| Week 4 | 1.00 | 1.12 (0.76,1.65) | 1.27 (0.82,1.95) | 0.96 (0.56,1.65) |
| Week 5 | 1.00 | 0.83 (0.57,1.22) | 0.85 (0.55,1.31) | 0.91 (0.53,1.55) |
| Week 6 | 1.00 | 1.26 (0.85,1.87) | 1.32 (0.85,2.05) | 1.53 (0.90,2.61) |
| Week 7 | 1.00 | 1.13 (0.77,1.67) | 1.16 (0.75,1.78) | 1.59 (0.94,2.69) |
| Week 8 | 1.00 | 0.96 (0.67,1.38) | 0.94 (0.63,1.41) | 0.84 (0.51,1.40) |
| d-TGA[c] | | | | |
| Week 2 | 1.00 | 0.98 (0.59,1.64) | 0.73 (0.41,1.29) | 0.64 (0.31,1.33) |
| Week 3 | 1.00 | 1.07 (0.61,1.86) | 1.00 (0.54,1.84) | 0.94 (0.44,2.01) |
| Week 4 | 1.00 | 1.15 (0.65,2.03) | 1.65 (0.89,3.07) | 1.01 (0.46,2.20) |
| Week 5 | 1.00 | 0.83 (0.48,1.43) | 0.99 (0.54,1.82) | 0.87 (0.40,1.85) |
| Week 6 | 1.00 | 1.12 (0.64,1.94) | 1.16 (0.63,2.14) | 1.34 (0.63,2.81) |
| Week 7 | 1.00 | 1.12 (0.64,1.94) | 1.08 (0.59,1.99) | 1.42 (0.68,2.97) |
| Week 8 | 1.00 | 1.00 (0.59,1.71) | 0.94 (0.52,1.69) | 1.08 (0.53,2.21) |
| TOF[c] | | | | |
| Week 2 | 1.00 | 1.30 (0.80,2.09) | 1.36 (0.81,2.31) | 1.29 (0.68,2.44) |
| Week 3 | 1.00 | 0.84 (0.53,1.35) | 0.82 (0.48,1.38) | 0.56 (0.29,1.09) |
| Week 4 | 1.00 | 0.94 (0.59,1.51) | 0.95 (0.57,1.60) | 0.73 (0.38,1.40) |
| Week 5 | 1.00 | 1.09 (0.67,1.77) | 0.94 (0.55,1.63) | 1.24 (0.64,2.39) |
| Week 6 | 1.00 | 1.24 (0.76,2.03) | 1.39 (0.81,2.39) | 1.60 (0.83,3.10) |
| Week 7 | 1.00 | 1.24 (0.76,2.01) | 1.32 (0.77,2.25) | 1.73 (0.91,3.32) |
| Week 8 | 1.00 | 0.91 (0.58,1.43) | 0.92 (0.56,1.52) | 0.71 (0.38,1.35) |
| Other conotruncals[c,d] | | | | |
| Week 2 | 1.00 | 1.15 (0.58,2.29) | 0.84 (0.40,1.76) | 0.70 (0.28,1.76) |
| Week 3 | 1.00 | 0.94 (0.45,1.96) | 1.49 (0.69,3.21) | 1.31 (0.51,3.34) |
| Week 4 | 1.00 | 1.31 (0.63,2.74) | 1.15 (0.53,2.54) | 1.32 (0.52,3.32) |
| Week 5 | 1.00 | 0.57 (0.29,1.13) | 0.81 (0.39,1.69) | 0.68 (0.27,1.74) |
| Week 6 | 1.00 | 1.39 (0.68,2.85) | 1.07 (0.49,2.33) | 1.18 (0.47,2.99) |
| Week 7 | 1.00 | 0.88 (0.44,1.75) | 0.88 (0.42,1.87) | 1.61 (0.66,3.93) |
| Week 8 | 1.00 | 0.96 (0.49,1.91) | 0.91 (0.43,1.90) | 0.74 (0.29,1.85) |
| APVR[b] | | | | |
| Week 2 | 1.00 | 0.67 (0.35,1.30) | 0.84 (0.40,1.77) | 1.16 (0.47,2.87) |
| Week 3 | 1.00 | 1.03 (0.52,2.04) | 1.23 (0.56,2.67) | 1.12 (0.43,2.92) |
| Week 4 | 1.00 | 0.79 (0.40,1.55) | 0.88 (0.41,1.90) | 0.96 (0.37,2.48) |
| Week 5 | 1.00 | 1.27 (0.64,2.51) | 0.81 (0.37,1.81) | 1.17 (0.45,3.05) |
| Week 6 | 1.00 | 1.09 (0.56,2.13) | 0.93 (0.43,2.03) | 0.49 (0.18,1.34) |
| Week 7 | 1.00 | 0.51 (0.27,0.98) | 0.47 (0.22,0.99) | 0.93 (0.37,2.35) |
| Week 8 | 1.00 | 0.81 (0.42,1.56) | 0.83 (0.39,1.77) | 0.97 (0.39,2.45) |
| TAPVR[c] | | | | |
| Week 2 | 1.00 | 0.71 (0.37,1.38) | 0.91 (0.43,1.92) | 1.02 (0.40,2.58) |

| Exposure and Defect | <10th centile | 10-<50th centile | 50-<90th centile | ≥90th centile |
|---|---|---|---|---|
| Week 3 | 1.00 | 1.05 (0.52,2.11) | 1.06 (0.48,2.33) | 1.16 (0.44,3.05) |
| Week 4 | 1.00 | 0.84 (0.42,1.65) | 0.79 (0.36,1.73) | 1.04 (0.4,2.7) |
| Week 5 | 1.00 | 1.36 (0.68,2.72) | 0.80 (0.36,1.80) | 1.15 (0.44,3.04) |
| Week 6 | 1.00 | 1.15 (0.59,2.27) | 0.94 (0.43,2.07) | 0.53 (0.19,1.45) |
| Week 7 | 1.00 | 0.57 (0.30,1.10) | 0.53 (0.25,1.15) | 1.25 (0.49,3.16) |
| Week 8 | 1.00 | 0.82 (0.43,1.59) | 0.76 (0.36,1.63) | 1.04 (0.41,2.63) |
| AVSD[b] | | | | |
| Week 2 | 1.00 | 0.73 (0.31,1.70) | 0.76 (0.31,1.86) | 2.00 (0.74,5.45) |
| Week 3 | 1.00 | 1.14 (0.48,2.74) | 1.43 (0.57,3.56) | 0.77 (0.23,2.58) |
| Week 4 | 1.00 | 1.24 (0.51,3.04) | 1.13 (0.44,2.87) | 0.94 (0.30,2.98) |
| Week 5 | 1.00 | 1.05 (0.45,2.46) | 1.07 (0.43,2.69) | 0.57 (0.15,2.13) |
| Week 6 | 1.00 | 1.05 (0.45,2.40) | 0.97 (0.39,2.36) | 0.87 (0.28,2.72) |
| Week 7 | 1.00 | 0.88 (0.38,2.05) | 1.02 (0.42,2.49) | 1.00 (0.32,3.12) |
| Week 8 | 1.00 | 0.97 (0.43,2.2) | 0.77 (0.32,1.86) | 0.75 (0.24,2.30) |
| RVOTO[b] | | | | |
| Week 2 | 1.00 | 0.61 (0.42,0.91) | 0.64 (0.41,1.00) | 0.45 (0.25,0.80) |
| Week 3 | 1.00 | 1.33 (0.85,2.10) | 1.15 (0.69,1.92) | 1.03 (0.55,1.93) |
| Week 4 | 1.00 | 0.92 (0.60,1.42) | 0.81 (0.49,1.31) | 0.94 (0.52,1.71) |
| Week 5 | 1.00 | 1.11 (0.71,1.74) | 1.26 (0.76,2.09) | 1.37 (0.73,2.55) |
| Week 6 | 1.00 | 1.45 (0.93,2.28) | 1.18 (0.71,1.95) | 1.46 (0.79,2.70) |
| Week 7 | 1.00 | 0.83 (0.54,1.28) | 0.95 (0.59,1.55) | 1.48 (0.83,2.67) |
| Week 8 | 1.00 | 1.15 (0.75,1.77) | 1.31 (0.81,2.12) | 0.81 (0.44,1.50) |
| Pulmonary/tricuspid atresia[c] | | | | |
| Week 2 | 1.00 | 0.92 (0.48,1.78) | 0.78 (0.37,1.62) | 0.92 (0.37,2.31) |
| Week 3 | 1.00 | 0.91 (0.47,1.78) | 0.58 (0.27,1.24) | 0.81 (0.32,2.09) |
| Week 4 | 1.00 | 1.47 (0.71,3.02) | 0.99 (0.45,2.17) | 0.99 (0.38,2.60) |
| Week 5 | 1.00 | 1.39 (0.66,2.93) | 1.56 (0.70,3.46) | 1.10 (0.40,2.97) |
| Week 6 | 1.00 | 0.97 (0.48,1.93) | 0.97 (0.45,2.08) | 1.61 (0.64,4.06) |
| Week 7 | 1.00 | 0.99 (0.49,1.97) | 1.14 (0.54,2.44) | 1.45 (0.57,3.73) |
| Week 8 | 1.00 | 0.72 (0.38,1.38) | 0.89 (0.44,1.82) | 0.51 (0.19,1.36) |
| PVS[c] | | | | |
| Week 2 | 1.00 | 0.53 (0.34,0.81) | 0.63 (0.39,1.02) | 0.37 (0.19,0.70) |
| Week 3 | 1.00 | 1.42 (0.86,2.37) | 1.38 (0.78,2.42) | 1.07 (0.54,2.14) |
| Week 4 | 1.00 | 0.84 (0.53,1.35) | 0.72 (0.42,1.22) | 0.82 (0.43,1.56) |
| Week 5 | 1.00 | 0.94 (0.57,1.53) | 1.00 (0.58,1.72) | 1.30 (0.67,2.53) |
| Week 6 | 1.00 | 1.57 (0.95,2.60) | 1.31 (0.75,2.29) | 1.50 (0.77,2.94) |
| Week 7 | 1.00 | 0.78 (0.49,1.25) | 0.88 (0.52,1.49) | 1.52 (0.81,2.87) |
| Week 8 | 1.00 | 1.43 (0.87,2.35) | 1.58 (0.91,2.73) | 1.01 (0.51,1.98) |
| Septal[b] | | | | |
| Week 2 | 1.00 | 1.07 (0.76,1.5) | 1.03 (0.70,1.5) | 0.90 (0.57,1.42) |
| Week 3 | 1.00 | 0.93 (0.65,1.33) | 1.07 (0.72,1.61) | 1.20 (0.74,1.95) |
| Week 4 | 1.00 | 1.16 (0.81,1.65) | 1.16 (0.78,1.74) | 1.10 (0.68,1.78) |
| Week 5 | 1.00 | 0.99 (0.69,1.42) | 1.04 (0.69,1.57) | 1.23 (0.75,2.00) |

| Exposure and Defect | <10th centile | 10-<50th centile | 50-<90th centile | ≥90th centile |
|---|---|---|---|---|
| Week 6 | 1.00 | 1.00 (0.71,1.42) | 0.84 (0.56,1.24) | 0.92 (0.57,1.48) |
| Week 7 | 1.00 | 1.24 (0.87,1.77) | 1.25 (0.84,1.87) | 1.31 (0.81,2.13) |
| Week 8 | 1.00 | 0.75 (0.54,1.03) | 0.85 (0.59,1.22) | 0.79 (0.50,1.25) |
| $VSD_{pm}$[c] | | | | |
| Week 2 | 1.00 | 1.08 (0.71,1.65) | 1.05 (0.65,1.68) | 0.95 (0.54,1.69) |
| Week 3 | 1.00 | 0.93 (0.60,1.46) | 1.09 (0.67,1.80) | 1.07 (0.59,1.96) |
| Week 4 | 1.00 | 0.91 (0.59,1.40) | 0.98 (0.60,1.59) | 0.90 (0.50,1.62) |
| Week 5 | 1.00 | 0.87 (0.56,1.36) | 0.99 (0.60,1.64) | 0.97 (0.53,1.77) |
| Week 6 | 1.00 | 1.04 (0.68,1.61) | 0.86 (0.53,1.40) | 0.76 (0.42,1.38) |
| Week 7 | 1.00 | 1.20 (0.77,1.88) | 1.35 (0.82,2.21) | 1.79 (0.99,3.23) |
| Week 8 | 1.00 | 0.84 (0.55,1.27) | 0.83 (0.53,1.32) | 0.87 (0.49,1.53) |
| ASD[c] | | | | |
| Week 2 | 1.00 | 1.07 (0.69,1.67) | 0.94 (0.57,1.54) | 0.67 (0.36,1.25) |
| Week 3 | 1.00 | 0.88 (0.55,1.41) | 1.00 (0.59,1.69) | 1.04 (0.55,1.98) |
| Week 4 | 1.00 | 1.42 (0.88,2.30) | 1.30 (0.76,2.21) | 1.19 (0.62,2.29) |
| Week 5 | 1.00 | 1.14 (0.71,1.85) | 1.03 (0.60,1.77) | 1.58 (0.83,3.02) |
| Week 6 | 1.00 | 0.97 (0.61,1.52) | 0.90 (0.54,1.49) | 1.13 (0.60,2.13) |
| Week 7 | 1.00 | 1.26 (0.79,2.01) | 1.13 (0.67,1.91) | 0.99 (0.52,1.90) |
| Week 8 | 1.00 | 0.68 (0.44,1.04) | 0.83 (0.52,1.33) | 0.60 (0.33,1.12) |
| **$NO_2$ (ppb)** | | | | |
| Week 2 | <17.1 | 17.1-<33.0 | 33.0-<48.0 | ≥48.0 |
| Week 3 | <17.1 | 17.1-<33.0 | 33.0-<48.0 | ≥48.0 |
| Week 4 | <17 | 17.0-<33.2 | 33.2-<47.9 | ≥47.9 |
| Week 5 | <17.1 | 17.1-<32.9 | 32.9-<48.1 | ≥48.1 |
| Week 6 | <17.3 | 17.3-<32.9 | 32.9-<48.4 | ≥48.4 |
| Week 7 | <17.1 | 17.1-<32.9 | 32.9-<48.0 | ≥48.0 |
| Week 8 | <17.3 | 17.3-<32.7 | 32.7-<47.2 | ≥47.2 |
| LVOTO[b] [OR (95% CI)] | | | | |
| Week 2 | 1.00 | 0.92 (0.57,1.47) | 0.94 (0.56,1.56) | 1.10 (0.62,1.97) |
| Week 3 | 1.00 | 1.22 (0.74,2.03) | 1.37 (0.80,2.36) | 0.79 (0.41,1.52) |
| Week 4 | 1.00 | 1.34 (0.82,2.21) | 1.10 (0.64,1.89) | 1.20 (0.64,2.23) |
| Week 5 | 1.00 | 1.03 (0.62,1.70) | 1.08 (0.63,1.86) | 0.98 (0.52,1.85) |
| Week 6 | 1.00 | 1.03 (0.63,1.69) | 1.08 (0.63,1.85) | 1.00 (0.53,1.87) |
| Week 7 | 1.00 | 1.24 (0.75,2.02) | 1.34 (0.78,2.29) | 1.58 (0.86,2.92) |
| Week 8 | 1.00 | 0.68 (0.44,1.06) | 0.68 (0.42,1.09) | 0.76 (0.43,1.33) |
| Aortic stenosis[c] | | | | |
| Week 2 | 1.00 | 0.89 (0.44,1.80) | 0.72 (0.34,1.53) | 1.14 (0.48,2.72) |
| Week 3 | 1.00 | 1.73 (0.78,3.88) | 2.38 (1.03,5.46) | 0.63 (0.21,1.89) |
| Week 4 | 1.00 | 1.27 (0.59,2.71) | 1.11 (0.50,2.48) | 1.94 (0.78,4.81) |
| Week 5 | 1.00 | 0.96 (0.46,1.98) | 0.79 (0.36,1.72) | 0.67 (0.26,1.75) |
| Week 6 | 1.00 | 0.95 (0.47,1.92) | 0.68 (0.32,1.48) | 0.57 (0.22,1.47) |
| Week 7 | 1.00 | 0.96 (0.47,1.95) | 0.90 (0.42,1.94) | 0.98 (0.40,2.43) |
| Week 8 | 1.00 | 0.72 (0.35,1.48) | 0.96 (0.45,2.03) | 1.72 (0.73,4.04) |

| Exposure and Defect | <10th centile | 10-<50th centile | 50-<90th centile | ≥90th centile |
|---|---|---|---|---|
| COA[c] | | | | |
| Week 2 | 1.00 | 1.10 (0.57,2.13) | 0.92 (0.46,1.84) | 1.35 (0.63,2.89) |
| Week 3 | 1.00 | 1.16 (0.60,2.24) | 1.36 (0.68,2.70) | 0.74 (0.33,1.68) |
| Week 4 | 1.00 | 1.65 (0.82,3.32) | 1.53 (0.74,3.19) | 1.48 (0.65,3.37) |
| Week 5 | 1.00 | 1.22 (0.58,2.54) | 1.28 (0.60,2.73) | 1.55 (0.68,3.58) |
| Week 6 | 1.00 | 0.92 (0.47,1.80) | 1.23 (0.61,2.48) | 1.32 (0.60,2.91) |
| Week 7 | 1.00 | 1.31 (0.66,2.60) | 1.40 (0.68,2.86) | 1.79 (0.81,3.96) |
| Week 8 | 1.00 | 0.74 (0.41,1.34) | 0.67 (0.36,1.25) | 0.62 (0.30,1.31) |
| HLHS[c] | | | | |
| Week 2 | 1.00 | 0.87 (0.47,1.60) | 1.17 (0.61,2.23) | 0.85 (0.38,1.86) |
| Week 3 | 1.00 | 1.09 (0.58,2.05) | 1.09 (0.55,2.15) | 1.07 (0.48,2.38) |
| Week 4 | 1.00 | 1.27 (0.69,2.36) | 0.90 (0.46,1.77) | 0.83 (0.37,1.88) |
| Week 5 | 1.00 | 0.86 (0.46,1.61) | 0.99 (0.51,1.94) | 0.63 (0.27,1.47) |
| Week 6 | 1.00 | 1.10 (0.58,2.09) | 1.11 (0.56,2.20) | 0.89 (0.39,2.04) |
| Week 7 | 1.00 | 1.21 (0.64,2.28) | 1.37 (0.69,2.70) | 1.34 (0.60,2.98) |
| Week 8 | 1.00 | 0.67 (0.38,1.18) | 0.67 (0.37,1.24) | 0.76 (0.36,1.61) |
| Conotruncal[b] | | | | |
| Week 2 | 1.00 | 0.95 (0.61,1.46) | 1.11 (0.70,1.77) | 1.15 (0.67,1.96) |
| Week 3 | 1.00 | 1.87 (1.14,3.08) | 1.63 (0.96,2.79) | 1.60 (0.88,2.93) |
| Week 4 | 1.00 | 1.57 (0.98,2.50) | 1.39 (0.84,2.30) | 1.45 (0.81,2.59) |
| Week 5 | 1.00 | 0.51 (0.34,0.76) | 0.49 (0.31,0.77) | 0.48 (0.28,0.83) |
| Week 6 | 1.00 | 1.14 (0.72,1.80) | 1.40 (0.85,2.29) | 1.21 (0.68,2.17) |
| Week 7 | 1.00 | 1.06 (0.69,1.63) | 0.83 (0.51,1.33) | 0.91 (0.52,1.58) |
| Week 8 | 1.00 | 0.90 (0.59,1.37) | 1.13 (0.71,1.77) | 1.19 (0.70,2.03) |
| d-TGA[c] | | | | |
| Week 2 | 1.00 | 0.88 (0.49,1.56) | 0.97 (0.52,1.80) | 0.87 (0.41,1.82) |
| Week 3 | 1.00 | 1.73 (0.91,3.31) | 1.23 (0.62,2.47) | 1.24 (0.56,2.73) |
| Week 4 | 1.00 | 1.56 (0.82,2.94) | 1.37 (0.69,2.72) | 1.67 (0.76,3.64) |
| Week 5 | 1.00 | 0.60 (0.34,1.04) | 0.54 (0.29,0.99) | 0.64 (0.30,1.34) |
| Week 6 | 1.00 | 0.97 (0.52,1.82) | 1.21 (0.62,2.36) | 1.55 (0.72,3.34) |
| Week 7 | 1.00 | 1.15 (0.64,2.06) | 0.94 (0.49,1.77) | 0.74 (0.34,1.61) |
| Week 8 | 1.00 | 0.90 (0.50,1.62) | 1.21 (0.65,2.26) | 1.23 (0.59,2.59) |
| TOF[c] | | | | |
| Week 2 | 1.00 | 0.97 (0.57,1.65) | 1.11 (0.63,1.94) | 1.37 (0.72,2.60) |
| Week 3 | 1.00 | 1.65 (0.91,2.99) | 1.60 (0.85,2.99) | 1.40 (0.68,2.86) |
| Week 4 | 1.00 | 1.77 (0.99,3.14) | 1.51 (0.81,2.80) | 1.46 (0.72,2.96) |
| Week 5 | 1.00 | 0.53 (0.33,0.86) | 0.55 (0.32,0.93) | 0.47 (0.24,0.92) |
| Week 6 | 1.00 | 1.22 (0.70,2.14) | 1.42 (0.78,2.58) | 0.97 (0.47,1.98) |
| Week 7 | 1.00 | 1.00 (0.60,1.68) | 0.79 (0.45,1.39) | 1.19 (0.62,2.27) |
| Week 8 | 1.00 | 0.91 (0.55,1.52) | 1.04 (0.60,1.80) | 1.10 (0.58,2.11) |
| Other conotruncals[c,d] | | | | |
| Week 2 | 1.00 | 0.99 (0.43,2.24) | 1.43 (0.63,3.27) | 1.00 (0.38,2.64) |
| Week 3 | 1.00 | 1.45 (0.61,3.45) | 1.33 (0.55,3.19) | 1.58 (0.60,4.16) |
| Week 4 | 1.00 | 1.01 (0.47,2.16) | 1.02 (0.46,2.28) | 1.01 (0.39,2.61) |

| Exposure and Defect | <10th centile | 10-<50th centile | 50-<90th centile | ≥90th centile |
|---|---|---|---|---|
| Week 5 | 1.00 | 0.72 (0.33,1.56) | 0.78 (0.35,1.76) | 0.86 (0.34,2.21) |
| Week 6 | 1.00 | 0.79 (0.36,1.71) | 1.02 (0.46,2.28) | 0.69 (0.26,1.86) |
| Week 7 | 1.00 | 1.67 (0.73,3.81) | 1.21 (0.52,2.83) | 0.88 (0.32,2.41) |
| Week 8 | 1.00 | 0.71 (0.33,1.51) | 0.99 (0.45,2.17) | 1.17 (0.47,2.92) |
| APVR[b] | | | | |
| Week 2 | 1.00 | 0.77 (0.36,1.66) | 0.65 (0.29,1.47) | 1.50 (0.61,3.71) |
| Week 3 | 1.00 | 1.33 (0.60,2.95) | 0.98 (0.42,2.31) | 1.02 (0.38,2.72) |
| Week 4 | 1.00 | 1.01 (0.47,2.16) | 0.90 (0.39,2.06) | 1.15 (0.44,3.00) |
| Week 5 | 1.00 | 0.53 (0.24,1.16) | 0.94 (0.42,2.15) | 1.53 (0.59,3.97) |
| Week 6 | 1.00 | 0.94 (0.44,2.03) | 0.85 (0.37,1.96) | 0.64 (0.23,1.75) |
| Week 7 | 1.00 | 1.37 (0.63,3.02) | 1.26 (0.54,2.93) | 0.63 (0.22,1.81) |
| Week 8 | 1.00 | 0.93 (0.44,1.96) | 0.53 (0.23,1.22) | 1.51 (0.61,3.71) |
| TAPVR[c] | | | | |
| Week 2 | 1.00 | 0.71 (0.34,1.52) | 0.60 (0.26,1.36) | 1.43 (0.58,3.53) |
| Week 3 | 1.00 | 1.30 (0.59,2.87) | 0.79 (0.34,1.86) | 1.01 (0.38,2.64) |
| Week 4 | 1.00 | 0.98 (0.46,2.10) | 0.85 (0.37,1.95) | 1.10 (0.42,2.88) |
| Week 5 | 1.00 | 0.54 (0.24,1.20) | 1.09 (0.48,2.49) | 1.38 (0.52,3.62) |
| Week 6 | 1.00 | 1.09 (0.50,2.39) | 0.79 (0.34,1.82) | 0.57 (0.21,1.59) |
| Week 7 | 1.00 | 1.37 (0.61,3.04) | 1.44 (0.62,3.34) | 0.72 (0.25,2.05) |
| Week 8 | 1.00 | 0.77 (0.37,1.62) | 0.50 (0.22,1.14) | 1.65 (0.68,4.01) |
| AVSD[b] | | | | |
| Week 2 | 1.00 | 0.64 (0.26,1.52) | 0.80 (0.33,1.95) | 1.35 (0.49,3.71) |
| Week 3 | 1.00 | 0.98 (0.39,2.43) | 1.33 (0.53,3.37) | 1.22 (0.41,3.57) |
| Week 4 | 1.00 | 0.94 (0.39,2.24) | 1.00 (0.40,2.51) | 0.95 (0.32,2.84) |
| Week 5 | 1.00 | 0.63 (0.27,1.48) | 0.67 (0.27,1.66) | 0.64 (0.20,1.98) |
| Week 6 | 1.00 | 1.43 (0.58,3.54) | 1.11 (0.43,2.90) | 0.59 (0.17,1.99) |
| Week 7 | 1.00 | 0.96 (0.41,2.26) | 0.76 (0.30,1.89) | 1.05 (0.37,3.02) |
| Week 8 | 1.00 | 0.8 (0.34,1.91) | 1.00 (0.41,2.45) | 0.68 (0.21,2.24) |
| RVOTO[b] | | | | |
| Week 2 | 1.00 | 1.02 (0.62,1.70) | 0.85 (0.49,1.48) | 0.71 (0.38,1.34) |
| Week 3 | 1.00 | 0.94 (0.55,1.60) | 1.29 (0.73,2.29) | 1.23 (0.64,2.36) |
| Week 4 | 1.00 | 1.29 (0.76,2.20) | 0.90 (0.50,1.60) | 1.02 (0.53,1.95) |
| Week 5 | 1.00 | 0.72 (0.43,1.21) | 1.04 (0.59,1.82) | 1.13 (0.59,2.16) |
| Week 6 | 1.00 | 0.74 (0.44,1.23) | 0.96 (0.55,1.68) | 1.02 (0.54,1.93) |
| Week 7 | 1.00 | 1.38 (0.81,2.37) | 1.33 (0.74,2.39) | 1.27 (0.66,2.45) |
| Week 8 | 1.00 | 1.04 (0.62,1.75) | 0.97 (0.55,1.68) | 1.53 (0.83,2.83) |
| Pulmonary/tricuspid atresia[c] | | | | |
| Week 2 | 1.00 | 0.93 (0.43,1.99) | 0.98 (0.44,2.18) | 1.03 (0.40,2.63) |
| Week 3 | 1.00 | 0.72 (0.34,1.52) | 1.08 (0.49,2.36) | 0.96 (0.36,2.53) |
| Week 4 | 1.00 | 1.43 (0.65,3.15) | 1.52 (0.66,3.51) | 0.94 (0.33,2.69) |
| Week 5 | 1.00 | 1.16 (0.51,2.64) | 1.32 (0.56,3.10) | 0.76 (0.26,2.20) |
| Week 6 | 1.00 | 0.62 (0.30,1.27) | 0.70 (0.32,1.52) | 0.59 (0.22,1.59) |
| Week 7 | 1.00 | 1.77 (0.81,3.87) | 0.93 (0.40,2.16) | 0.93 (0.35,2.52) |

| Exposure and Defect | <10th centile | 10-<50th centile | 50-<90th centile | ≥90th centile |
|---|---|---|---|---|
| Week 8 | 1.00 | 0.68 (0.33,1.40) | 0.68 (0.31,1.47) | 1.19 (0.48,2.92) |
| PVS[c] | | | | |
| Week 2 | 1.00 | 0.95 (0.55,1.65) | 0.76 (0.42,1.37) | 0.66 (0.33,1.29) |
| Week 3 | 1.00 | 1.02 (0.56,1.85) | 1.33 (0.71,2.49) | 1.22 (0.60,2.48) |
| Week 4 | 1.00 | 1.34 (0.75,2.39) | 0.82 (0.44,1.53) | 0.97 (0.48,1.95) |
| Week 5 | 1.00 | 0.55 (0.31,0.96) | 0.85 (0.47,1.54) | 1.14 (0.57,2.24) |
| Week 6 | 1.00 | 0.90 (0.50,1.63) | 1.22 (0.65,2.28) | 1.38 (0.69,2.80) |
| Week 7 | 1.00 | 1.15 (0.64,2.05) | 1.32 (0.71,2.46) | 1.31 (0.65,2.63) |
| Week 8 | 1.00 | 1.10 (0.62,1.96) | 1.05 (0.57,1.93) | 1.53 (0.78,3.00) |
| Septal[b] | | | | |
| Week 2 | 1.00 | 0.99 (0.67,1.44) | 0.92 (0.61,1.39) | 0.80 (0.49,1.30) |
| Week 3 | 1.00 | 1.11 (0.74,1.67) | 1.05 (0.67,1.63) | 0.84 (0.50,1.41) |
| Week 4 | 1.00 | 1.52 (1.00,2.32) | 1.51 (0.96,2.39) | 1.48 (0.88,2.51) |
| Week 5 | 1.00 | 0.99 (0.66,1.49) | 1.15 (0.74,1.80) | 1.56 (0.93,2.60) |
| Week 6 | 1.00 | 0.68 (0.46,0.99) | 0.74 (0.49,1.13) | 0.63 (0.38,1.05) |
| Week 7 | 1.00 | 1.15 (0.78,1.69) | 0.88 (0.57,1.35) | 0.92 (0.56,1.52) |
| Week 8 | 1.00 | 1.06 (0.72,1.56) | 1.18 (0.77,1.80) | 1.82 (1.13,2.94) |
| $VSD_{pm}$[c] | | | | |
| Week 2 | 1.00 | 1.09 (0.67,1.78) | 0.95 (0.56,1.61) | 0.94 (0.51,1.73) |
| Week 3 | 1.00 | 1.14 (0.68,1.91) | 1.18 (0.68,2.06) | 0.89 (0.47,1.71) |
| Week 4 | 1.00 | 1.24 (0.74,2.06) | 1.31 (0.75,2.28) | 1.09 (0.57,2.09) |
| Week 5 | 1.00 | 1.03 (0.61,1.75) | 1.23 (0.70,2.16) | 1.75 (0.92,3.33) |
| Week 6 | 1.00 | 0.70 (0.43,1.12) | 0.79 (0.47,1.34) | 0.63 (0.34,1.18) |
| Week 7 | 1.00 | 1.23 (0.75,2.01) | 0.77 (0.45,1.32) | 0.95 (0.51,1.77) |
| Week 8 | 1.00 | 0.94 (0.58,1.53) | 1.05 (0.62,1.78) | 1.88 (1.04,3.40) |
| ASD[c] | | | | |
| Week 2 | 1.00 | 0.94 (0.58,1.52) | 0.90 (0.54,1.52) | 0.65 (0.34,1.22) |
| Week 3 | 1.00 | 1.06 (0.63,1.77) | 0.99 (0.57,1.73) | 0.80 (0.41,1.55) |
| Week 4 | 1.00 | 1.60 (0.93,2.77) | 1.52 (0.85,2.75) | 1.60 (0.81,3.14) |
| Week 5 | 1.00 | 0.74 (0.45,1.22) | 0.87 (0.50,1.50) | 1.05 (0.55,2.01) |
| Week 6 | 1.00 | 0.77 (0.47,1.26) | 0.81 (0.47,1.39) | 0.76 (0.40,1.45) |
| Week 7 | 1.00 | 1.26 (0.75,2.11) | 1.18 (0.67,2.07) | 1.12 (0.58,2.16) |
| Week 8 | 1.00 | 1.14 (0.68,1.90) | 1.24 (0.71,2.14) | 1.49 (0.79,2.81) |
| **Ozone (ppb)[e]** | | | | |
| Week 2 | <30.6 | 30.6-<42.1 | 42.1-<53.1 | ≥53.1 |
| Week 3 | <30.6 | 30.6-<42.0 | 42.0-<52.9 | ≥52.9 |
| Week 4 | <30.4 | 30.4-<42.3 | 42.3-<53.1 | ≥53.1 |
| Week 5 | <30.4 | 30.4-<42.1 | 42.1-<53.3 | ≥53.3 |
| Week 6 | <30.3 | 30.3-<42.1 | 42.1-<53.4 | ≥53.4 |
| Week 7 | <31 | 31.0-<42.4 | 42.4-<52.7 | ≥52.7 |
| Week 8 | <30.6 | 30.6-<41.9 | 41.9-<53.0 | ≥53.0 |

| Exposure and Defect | <10th centile | 10-<50th centile | 50-<90th centile | ≥90th centile |
|---|---|---|---|---|
| LVOTO[b] [OR (95% CI)] | | | | |
| Week 2 | 1.00 | 0.83 (0.59,1.18) | 0.96 (0.65,1.44) | 0.88 (0.57,1.37) |
| Week 3 | 1.00 | 1.13 (0.79,1.63) | 0.99 (0.64,1.52) | 1.12 (0.69,1.80) |
| Week 4 | 1.00 | 0.98 (0.68,1.42) | 0.95 (0.61,1.47) | 0.85 (0.52,1.38) |
| Week 5 | 1.00 | 0.65 (0.46,0.94) | 0.69 (0.45,1.06) | 0.73 (0.45,1.17) |
| Week 6 | 1.00 | 1.52 (1.05,2.20) | 1.34 (0.86,2.10) | 1.22 (0.74,2.01) |
| Week 7 | 1.00 | 1.18 (0.82,1.69) | 1.03 (0.67,1.58) | 1.24 (0.78,1.99) |
| Week 8 | 1.00 | 0.99 (0.70,1.38) | 1.10 (0.75,1.62) | 1.08 (0.70,1.67) |
| Aortic stenosis[c] | | | | |
| Week 2 | 1.00 | 0.96 (0.52,1.78) | 1.30 (0.68,2.52) | 0.98 (0.46,2.06) |
| Week 3 | 1.00 | 1.58 (0.85,2.96) | 1.13 (0.55,2.32) | 1.45 (0.66,3.16) |
| Week 4 | 1.00 | 1.16 (0.62,2.15) | 0.91 (0.44,1.85) | 1.16 (0.53,2.54) |
| Week 5 | 1.00 | 0.97 (0.53,1.78) | 0.72 (0.35,1.46) | 0.77 (0.35,1.71) |
| Week 6 | 1.00 | 1.38 (0.75,2.54) | 0.90 (0.44,1.84) | 0.74 (0.33,1.66) |
| Week 7 | 1.00 | 0.89 (0.49,1.63) | 0.71 (0.36,1.44) | 1.27 (0.60,2.69) |
| Week 8 | 1.00 | 1.29 (0.72,2.34) | 1.48 (0.77,2.84) | 1.00 (0.47,2.13) |
| COA[c] | | | | |
| Week 2 | 1.00 | 0.64 (0.38,1.07) | 0.79 (0.45,1.40) | 0.81 (0.44,1.49) |
| Week 3 | 1.00 | 1.10 (0.64,1.87) | 1.16 (0.63,2.11) | 1.22 (0.63,2.36) |
| Week 4 | 1.00 | 1.03 (0.60,1.77) | 1.07 (0.58,1.97) | 0.85 (0.43,1.66) |
| Week 5 | 1.00 | 0.55 (0.32,0.94) | 0.72 (0.39,1.31) | 0.94 (0.49,1.80) |
| Week 6 | 1.00 | 1.38 (0.82,2.33) | 1.34 (0.72,2.49) | 1.37 (0.70,2.70) |
| Week 7 | 1.00 | 1.11 (0.66,1.87) | 0.97 (0.53,1.77) | 1.27 (0.66,2.44) |
| Week 8 | 1.00 | 0.81 (0.50,1.31) | 0.83 (0.48,1.42) | 0.75 (0.41,1.36) |
| HLHS[c] | | | | |
| Week 2 | 1.00 | 0.89 (0.55,1.43) | 0.85 (0.49,1.47) | 0.78 (0.43,1.44) |
| Week 3 | 1.00 | 1.07 (0.65,1.77) | 0.85 (0.47,1.52) | 0.97 (0.51,1.84) |
| Week 4 | 1.00 | 0.86 (0.52,1.42) | 0.89 (0.50,1.59) | 0.77 (0.40,1.47) |
| Week 5 | 1.00 | 0.76 (0.46,1.26) | 0.93 (0.52,1.66) | 0.79 (0.41,1.52) |
| Week 6 | 1.00 | 1.57 (0.94,2.62) | 1.47 (0.81,2.68) | 1.30 (0.66,2.54) |
| Week 7 | 1.00 | 1.20 (0.73,1.99) | 1.03 (0.58,1.83) | 0.90 (0.47,1.72) |
| Week 8 | 1.00 | 1.10 (0.68,1.79) | 1.25 (0.73,2.14) | 1.65 (0.91,2.99) |
| Conotruncal[b] | | | | |
| Week 2 | 1.00 | 1.03 (0.76,1.39) | 0.73 (0.51,1.05) | 0.99 (0.67,1.46) |
| Week 3 | 1.00 | 1.26 (0.91,1.75) | 1.27 (0.87,1.86) | 1.19 (0.77,1.82) |
| Week 4 | 1.00 | 1.13 (0.81,1.57) | 0.85 (0.58,1.26) | 0.86 (0.56,1.33) |
| Week 5 | 1.00 | 1.14 (0.82,1.58) | 1.36 (0.92,2.01) | 1.38 (0.89,2.15) |
| Week 6 | 1.00 | 1.00 (0.73,1.38) | 1.04 (0.71,1.52) | 0.83 (0.53,1.29) |
| Week 7 | 1.00 | 0.84 (0.61,1.15) | 0.75 (0.52,1.09) | 0.75 (0.49,1.14) |
| Week 8 | 1.00 | 1.05 (0.78,1.41) | 1.01 (0.71,1.42) | 1.11 (0.75,1.63) |
| d-TGA[c] | | | | |
| Week 2 | 1.00 | 0.92 (0.58,1.47) | 1.01 (0.60,1.71) | 0.89 (0.50,1.59) |
| Week 3 | 1.00 | 1.33 (0.82,2.16) | 1.25 (0.72,2.18) | 1.18 (0.64,2.20) |

| Exposure and Defect | <10th centile | 10-<50th centile | 50-<90th centile | ≥90th centile |
|---|---|---|---|---|
| Week 4 | 1.00 | 0.88 (0.54,1.43) | 0.76 (0.44,1.34) | 0.83 (0.45,1.54) |
| Week 5 | 1.00 | 1.29 (0.80,2.09) | 1.53 (0.86,2.69) | 1.27 (0.67,2.41) |
| Week 6 | 1.00 | 0.86 (0.53,1.39) | 1.13 (0.65,1.95) | 0.91 (0.48,1.70) |
| Week 7 | 1.00 | 0.59 (0.37,0.95) | 0.66 (0.39,1.12) | 0.64 (0.35,1.17) |
| Week 8 | 1.00 | 1.48 (0.96,2.29) | 0.91 (0.53,1.54) | 1.18 (0.67,2.09) |
| TOF[c] | | | | |
| Week 2 | 1.00 | 1.11 (0.76,1.63) | 0.60 (0.37,0.96) | 1.13 (0.70,1.84) |
| Week 3 | 1.00 | 1.25 (0.83,1.89) | 1.26 (0.78,2.02) | 1.13 (0.66,1.94) |
| Week 4 | 1.00 | 1.21 (0.80,1.83) | 0.88 (0.54,1.45) | 0.81 (0.47,1.40) |
| Week 5 | 1.00 | 1.03 (0.68,1.56) | 1.16 (0.71,1.90) | 1.31 (0.76,2.26) |
| Week 6 | 1.00 | 1.09 (0.72,1.65) | 1.09 (0.67,1.77) | 0.88 (0.50,1.52) |
| Week 7 | 1.00 | 1.02 (0.68,1.53) | 0.84 (0.52,1.35) | 0.89 (0.53,1.51) |
| Week 8 | 1.00 | 0.90 (0.61,1.32) | 1.01 (0.66,1.56) | 1.03 (0.63,1.68) |
| Other conotruncals[c,d] | | | | |
| Week 2 | 1.00 | 1.03 (0.55,1.93) | 0.62 (0.30,1.29) | 0.89 (0.42,1.91) |
| Week 3 | 1.00 | 1.12 (0.57,2.18) | 1.27 (0.61,2.64) | 1.32 (0.59,2.99) |
| Week 4 | 1.00 | 1.63 (0.83,3.21) | 1.01 (0.46,2.21) | 1.20 (0.52,2.78) |
| Week 5 | 1.00 | 0.89 (0.45,1.74) | 1.16 (0.55,2.44) | 1.36 (0.59,3.11) |
| Week 6 | 1.00 | 1.28 (0.67,2.47) | 0.90 (0.43,1.91) | 0.72 (0.31,1.70) |
| Week 7 | 1.00 | 0.98 (0.52,1.88) | 0.74 (0.36,1.53) | 0.64 (0.28,1.46) |
| Week 8 | 1.00 | 0.71 (0.37,1.39) | 1.23 (0.63,2.40) | 1.19 (0.56,2.57) |
| APVR[b] | | | | |
| Week 2 | 1.00 | 0.77 (0.38,1.56) | 1.01 (0.48,2.10) | 1.19 (0.54,2.59) |
| Week 3 | 1.00 | 0.97 (0.48,1.96) | 1.44 (0.69,3.02) | 0.94 (0.41,2.17) |
| Week 4 | 1.00 | 1.04 (0.52,2.10) | 0.90 (0.41,1.95) | 0.99 (0.43,2.31) |
| Week 5 | 1.00 | 1.18 (0.59,2.36) | 1.19 (0.55,2.58) | 0.96 (0.41,2.25) |
| Week 6 | 1.00 | 0.90 (0.45,1.78) | 0.91 (0.42,1.96) | 1.07 (0.46,2.48) |
| Week 7 | 1.00 | 0.98 (0.50,1.91) | 0.71 (0.33,1.53) | 0.87 (0.38,1.99) |
| Week 8 | 1.00 | 1.05 (0.55,2.00) | 0.95 (0.46,1.95) | 1.04 (0.47,2.28) |
| TAPVR[c] | | | | |
| Week 2 | 1.00 | 0.71 (0.34,1.50) | 1.18 (0.56,2.48) | 1.36 (0.62,2.97) |
| Week 3 | 1.00 | 0.95 (0.46,1.97) | 1.42 (0.68,2.97) | 0.85 (0.37,1.96) |
| Week 4 | 1.00 | 0.98 (0.48,2.00) | 0.80 (0.37,1.75) | 0.98 (0.43,2.24) |
| Week 5 | 1.00 | 1.55 (0.77,3.14) | 1.38 (0.63,3.02) | 1.00 (0.42,2.38) |
| Week 6 | 1.00 | 0.80 (0.39,1.62) | 0.95 (0.44,2.04) | 1.05 (0.45,2.42) |
| Week 7 | 1.00 | 1.07 (0.54,2.11) | 0.72 (0.33,1.57) | 0.85 (0.37,1.93) |
| Week 8 | 1.00 | 1.09 (0.56,2.11) | 1.00 (0.48,2.09) | 1.14 (0.52,2.53) |
| AVSD[b] | | | | |
| Week 2 | 1.00 | 1.17 (0.51,2.68) | 1.03 (0.42,2.51) | 1.36 (0.53,3.49) |
| Week 3 | 1.00 | 1.03 (0.45,2.37) | 0.92 (0.37,2.28) | 1.34 (0.51,3.50) |
| Week 4 | 1.00 | 1.38 (0.60,3.17) | 0.91 (0.35,2.37) | 1.60 (0.60,4.23) |
| Week 5 | 1.00 | 0.61 (0.27,1.39) | 0.62 (0.25,1.55) | 0.81 (0.31,2.16) |
| Week 6 | 1.00 | 1.69 (0.77,3.71) | 0.88 (0.36,2.18) | 0.41 (0.14,1.17) |
| Week 7 | 1.00 | 0.92 (0.42,2.03) | 0.53 (0.21,1.34) | 0.85 (0.33,2.20) |

| Exposure and Defect | <10th centile | 10-<50th centile | 50-<90th centile | ≥90th centile |
|---|---|---|---|---|
| Week 8 | 1.00 | 0.85 (0.36,2.00) | 1.61 (0.69,3.72) | 1.39 (0.54,3.57) |
| RVOTO[b] | | | | |
| Week 2 | 1.00 | 0.87 (0.59,1.28) | 0.61 (0.39,0.95) | 0.72 (0.45,1.14) |
| Week 3 | 1.00 | 1.18 (0.78,1.80) | 1.86 (1.16,2.98) | 2.01 (1.21,3.36) |
| Week 4 | 1.00 | 0.91 (0.59,1.38) | 1.06 (0.66,1.71) | 0.84 (0.50,1.42) |
| Week 5 | 1.00 | 1.01 (0.67,1.52) | 1.02 (0.63,1.64) | 1.07 (0.64,1.80) |
| Week 6 | 1.00 | 1.07 (0.71,1.60) | 1.22 (0.76,1.95) | 1.13 (0.67,1.89) |
| Week 7 | 1.00 | 0.74 (0.50,1.10) | 0.64 (0.41,1.00) | 0.67 (0.41,1.09) |
| Week 8 | 1.00 | 1.17 (0.81,1.69) | 1.08 (0.71,1.65) | 1.24 (0.78,1.96) |
| Pulmonary/tricuspid atresia[c] | | | | |
| Week 2 | 1.00 | 1.05 (0.52,2.08) | 0.63 (0.29,1.36) | 1.15 (0.53,2.49) |
| Week 3 | 1.00 | 1.06 (0.51,2.19) | 1.66 (0.78,3.52) | 1.27 (0.55,2.91) |
| Week 4 | 1.00 | 0.88 (0.42,1.81) | 1.02 (0.48,2.18) | 0.83 (0.36,1.92) |
| Week 5 | 1.00 | 0.78 (0.38,1.62) | 1.24 (0.58,2.62) | 1.02 (0.44,2.33) |
| Week 6 | 1.00 | 1.06 (0.53,2.15) | 1.17 (0.55,2.51) | 1.17 (0.51,2.69) |
| Week 7 | 1.00 | 0.69 (0.35,1.37) | 0.71 (0.34,1.49) | 0.76 (0.34,1.70) |
| Week 8 | 1.00 | 1.98 (1.03,3.82) | 1.29 (0.61,2.72) | 1.22 (0.55,2.70) |
| PVS[c] | | | | |
| Week 2 | 1.00 | 0.78 (0.51,1.20) | 0.60 (0.37,0.97) | 0.59 (0.35,0.99) |
| Week 3 | 1.00 | 1.30 (0.81,2.08) | 1.93 (1.15,3.25) | 2.15 (1.22,3.78) |
| Week 4 | 1.00 | 1.08 (0.67,1.73) | 1.16 (0.69,1.98) | 0.94 (0.53,1.68) |
| Week 5 | 1.00 | 1.08 (0.68,1.71) | 1.02 (0.60,1.73) | 1.11 (0.63,1.98) |
| Week 6 | 1.00 | 1.14 (0.73,1.81) | 1.32 (0.78,2.22) | 1.24 (0.70,2.22) |
| Week 7 | 1.00 | 0.75 (0.48,1.16) | 0.59 (0.36,0.97) | 0.66 (0.38,1.14) |
| Week 8 | 1.00 | 0.90 (0.60,1.37) | 0.94 (0.59,1.50) | 1.14 (0.69,1.90) |
| Septal[b] | | | | |
| Week 2 | 1.00 | 1.02 (0.79,1.31) | 0.72 (0.53,0.97) | 0.83 (0.60,1.16) |
| Week 3 | 1.00 | 1.00 (0.77,1.30) | 0.90 (0.66,1.24) | 0.95 (0.67,1.36) |
| Week 4 | 1.00 | 1.10 (0.84,1.45) | 1.07 (0.77,1.49) | 1.00 (0.69,1.45) |
| Week 5 | 1.00 | 1.16 (0.88,1.53) | 1.40 (1.01,1.95) | 1.58 (1.09,2.29) |
| Week 6 | 1.00 | 1.09 (0.83,1.42) | 1.09 (0.79,1.51) | 0.87 (0.60,1.26) |
| Week 7 | 1.00 | 0.73 (0.56,0.95) | 0.57 (0.42,0.77) | 0.68 (0.48,0.96) |
| Week 8 | 1.00 | 1.17 (0.91,1.51) | 1.13 (0.84,1.52) | 1.33 (0.95,1.86) |
| $VSD_{pm}$[c] | | | | |
| Week 2 | 1.00 | 0.92 (0.64,1.32) | 0.86 (0.57,1.31) | 0.97 (0.61,1.52) |
| Week 3 | 1.00 | 1.11 (0.76,1.62) | 1.07 (0.69,1.66) | 1.02 (0.63,1.66) |
| Week 4 | 1.00 | 1.15 (0.78,1.71) | 1.05 (0.66,1.66) | 1.15 (0.70,1.90) |
| Week 5 | 1.00 | 1.21 (0.82,1.78) | 1.22 (0.77,1.93) | 1.33 (0.80,2.20) |
| Week 6 | 1.00 | 1.09 (0.75,1.60) | 1.16 (0.74,1.81) | 1.08 (0.65,1.79) |
| Week 7 | 1.00 | 0.74 (0.51,1.06) | 0.49 (0.32,0.75) | 0.56 (0.35,0.89) |
| Week 8 | 1.00 | 1.13 (0.79,1.60) | 0.98 (0.65,1.48) | 1.43 (0.91,2.24) |
| ASD[c] | | | | |
| Week 2 | 1.00 | 1.18 (0.87,1.61) | 0.64 (0.44,0.94) | 0.77 (0.50,1.18) |

| **Exposure and Defect** | **<10th centile** | **10-<50th centile** | **50-<90th centile** | **≥90th centile** |
|---|---|---|---|---|
| Week 3 | 1.00 | 0.94 (0.68,1.29) | 0.82 (0.56,1.21) | 0.92 (0.59,1.43) |
| Week 4 | 1.00 | 1.17 (0.83,1.63) | 1.16 (0.78,1.72) | 0.90 (0.56,1.44) |
| Week 5 | 1.00 | 1.09 (0.78,1.54) | 1.40 (0.93,2.10) | 1.76 (1.11,2.80) |
| Week 6 | 1.00 | 1.04 (0.75,1.45) | 1.02 (0.69,1.52) | 0.72 (0.45,1.16) |
| Week 7 | 1.00 | 0.79 (0.57,1.09) | 0.71 (0.48,1.05) | 0.94 (0.60,1.45) |
| Week 8 | 1.00 | 1.27 (0.92,1.74) | 1.24 (0.86,1.80) | 1.17 (0.76,1.81) |
| **$PM_{10}$ (μg/$m^3$)** | | | | |
| Week 2 | <11 | 11.0-<23.0 | 23.0-<46.2 | ≥46.2 |
| Week 3 | <11 | 11.0-<23.0 | 23.0-<46.0 | ≥46.0 |
| Week 4 | <11 | 11.0-<23.0 | 23.0-<45.0 | ≥45.0 |
| Week 5 | <11 | 11.0-<23.0 | 23.0-<46.0 | ≥46.0 |
| Week 6 | <11 | 11.0-<23.0 | 23.0-<47.0 | ≥47.0 |
| Week 7 | <11 | 11.0-<23.0 | 23.0-<46.0 | ≥46.0 |
| Week 8 | <11 | 11.0-<22.0 | 22.0-<45.0 | ≥45.0 |
| LVOTO[b] [OR (95% CI)] | | | | |
| Week 2 | 1.00 | 0.98 (0.66,1.43) | 1.10 (0.74,1.63) | 0.91 (0.55,1.51) |
| Week 3 | 1.00 | 0.89 (0.61,1.28) | 0.89 (0.61,1.30) | 0.85 (0.52,1.39) |
| Week 4 | 1.00 | 0.95 (0.65,1.39) | 1.00 (0.68,1.48) | 1.09 (0.66,1.77) |
| Week 5 | 1.00 | 1.10 (0.75,1.63) | 1.25 (0.84,1.86) | 0.88 (0.52,1.49) |
| Week 6 | 1.00 | 0.98 (0.67,1.43) | 0.98 (0.66,1.44) | 0.86 (0.51,1.45) |
| Week 7 | 1.00 | 1.43 (0.94,2.18) | 1.38 (0.90,2.12) | 1.53 (0.90,2.60) |
| Week 8 | 1.00 | 0.63 (0.44,0.91) | 0.81 (0.56,1.16) | 1.05 (0.66,1.65) |
| Aortic stenosis[c] | | | | |
| Week 2 | 1.00 | 1.02 (0.51,2.05) | 1.54 (0.77,3.07) | 1.00 (0.41,2.41) |
| Week 3 | 1.00 | 1.35 (0.71,2.57) | 1.03 (0.53,2.00) | 0.51 (0.18,1.42) |
| Week 4 | 1.00 | 0.94 (0.49,1.81) | 1.12 (0.58,2.14) | 0.96 (0.41,2.23) |
| Week 5 | 1.00 | 0.90 (0.49,1.64) | 0.88 (0.47,1.63) | 0.72 (0.30,1.73) |
| Week 6 | 1.00 | 0.73 (0.41,1.31) | 0.64 (0.35,1.17) | 0.77 (0.34,1.76) |
| Week 7 | 1.00 | 0.89 (0.48,1.66) | 1.06 (0.57,1.98) | 0.82 (0.35,1.96) |
| Week 8 | 1.00 | 0.46 (0.25,0.85) | 0.82 (0.46,1.46) | 1.02 (0.48,2.17) |
| COA[c] | | | | |
| Week 2 | 1.00 | 1.24 (0.70,2.19) | 1.19 (0.67,2.12) | 0.81 (0.38,1.73) |
| Week 3 | 1.00 | 0.91 (0.54,1.53) | 0.87 (0.51,1.49) | 1.12 (0.57,2.22) |
| Week 4 | 1.00 | 1.02 (0.58,1.78) | 1.07 (0.61,1.87) | 1.10 (0.54,2.22) |
| Week 5 | 1.00 | 1.78 (0.94,3.38) | 1.71 (0.90,3.26) | 1.13 (0.51,2.54) |
| Week 6 | 1.00 | 1.03 (0.59,1.77) | 0.93 (0.53,1.63) | 0.99 (0.48,2.04) |
| Week 7 | 1.00 | 1.40 (0.75,2.60) | 1.54 (0.83,2.87) | 1.82 (0.86,3.82) |
| Week 8 | 1.00 | 1.05 (0.58,1.89) | 1.22 (0.68,2.18) | 1.44 (0.71,2.90) |
| HLHS[c] | | | | |
| Week 2 | 1.00 | 0.76 (0.45,1.29) | 0.84 (0.50,1.43) | 0.91 (0.47,1.77) |
| Week 3 | 1.00 | 0.66 (0.39,1.10) | 0.91 (0.54,1.51) | 0.90 (0.47,1.75) |
| Week 4 | 1.00 | 0.92 (0.54,1.58) | 0.89 (0.52,1.54) | 1.01 (0.52,1.98) |
| Week 5 | 1.00 | 0.89 (0.51,1.55) | 1.32 (0.77,2.29) | 0.97 (0.47,1.98) |

| Exposure and Defect | <10th centile | 10-<50th centile | 50-<90th centile | ≥90th centile |
|---|---|---|---|---|
| Week 6 | 1.00 | 1.12 (0.63,1.98) | 1.31 (0.74,2.34) | 0.89 (0.42,1.88) |
| Week 7 | 1.00 | 1.97 (1.02,3.82) | 1.46 (0.75,2.86) | 1.69 (0.78,3.64) |
| Week 8 | 1.00 | 0.62 (0.37,1.03) | 0.72 (0.43,1.19) | 0.98 (0.52,1.85) |
| Conotruncal[b] | | | | |
| Week 2 | 1.00 | 1.01 (0.70,1.46) | 1.12 (0.77,1.62) | 1.00 (0.63,1.59) |
| Week 3 | 1.00 | 1.02 (0.71,1.46) | 1.00 (0.69,1.44) | 1.11 (0.7,1.75) |
| Week 4 | 1.00 | 0.76 (0.54,1.08) | 0.83 (0.58,1.18) | 0.98 (0.63,1.53) |
| Week 5 | 1.00 | 1.12 (0.78,1.62) | 1.19 (0.82,1.74) | 1.22 (0.76,1.95) |
| Week 6 | 1.00 | 0.89 (0.63,1.26) | 0.72 (0.50,1.03) | 0.90 (0.57,1.42) |
| Week 7 | 1.00 | 1.04 (0.73,1.50) | 1.11 (0.77,1.62) | 1.14 (0.72,1.82) |
| Week 8 | 1.00 | 0.71 (0.50,1.01) | 1.01 (0.71,1.43) | 1.06 (0.69,1.65) |
| d-TGA[c] | | | | |
| Week 2 | 1.00 | 1.04 (0.61,1.80) | 0.93 (0.53,1.64) | 1.00 (0.50,1.97) |
| Week 3 | 1.00 | 0.79 (0.47,1.32) | 0.76 (0.45,1.29) | 1.05 (0.55,2.01) |
| Week 4 | 1.00 | 0.78 (0.46,1.31) | 0.84 (0.49,1.43) | 1.07 (0.56,2.06) |
| Week 5 | 1.00 | 0.81 (0.48,1.35) | 0.88 (0.52,1.49) | 0.92 (0.47,1.80) |
| Week 6 | 1.00 | 1.25 (0.71,2.21) | 0.89 (0.50,1.61) | 1.10 (0.54,2.24) |
| Week 7 | 1.00 | 1.16 (0.66,2.04) | 1.30 (0.74,2.30) | 1.07 (0.53,2.19) |
| Week 8 | 1.00 | 0.92 (0.50,1.66) | 1.44 (0.80,2.58) | 1.52 (0.76,3.03) |
| TOF[c] | | | | |
| Week 2 | 1.00 | 1.09 (0.68,1.74) | 1.26 (0.78,2.04) | 1.14 (0.63,2.05) |
| Week 3 | 1.00 | 1.18 (0.74,1.88) | 1.10 (0.68,1.76) | 1.10 (0.61,1.98) |
| Week 4 | 1.00 | 0.69 (0.45,1.05) | 0.82 (0.53,1.26) | 0.87 (0.50,1.51) |
| Week 5 | 1.00 | 1.62 (0.96,2.74) | 1.66 (0.97,2.83) | 1.62 (0.86,3.05) |
| Week 6 | 1.00 | 0.84 (0.55,1.28) | 0.71 (0.46,1.10) | 0.81 (0.46,1.43) |
| Week 7 | 1.00 | 0.92 (0.59,1.43) | 0.93 (0.59,1.46) | 1.09 (0.62,1.92) |
| Week 8 | 1.00 | 0.58 (0.38,0.89) | 0.87 (0.57,1.31) | 0.95 (0.56,1.62) |
| Other conotruncals[c,d] | | | | |
| Week 2 | 1.00 | 0.70 (0.35,1.40) | 1.03 (0.53,2.02) | 0.63 (0.25,1.57) |
| Week 3 | 1.00 | 0.99 (0.47,2.10) | 1.19 (0.57,2.48) | 1.12 (0.46,2.74) |
| Week 4 | 1.00 | 1.10 (0.53,2.28) | 0.82 (0.39,1.73) | 1.12 (0.47,2.68) |
| Week 5 | 1.00 | 0.76 (0.38,1.49) | 0.86 (0.43,1.69) | 1.02 (0.43,2.39) |
| Week 6 | 1.00 | 0.65 (0.34,1.27) | 0.61 (0.31,1.20) | 1.00 (0.44,2.29) |
| Week 7 | 1.00 | 1.17 (0.54,2.54) | 1.31 (0.61,2.83) | 1.41 (0.57,3.49) |
| Week 8 | 1.00 | 0.86 (0.43,1.72) | 0.83 (0.42,1.66) | 0.77 (0.32,1.84) |
| APVR[b] | | | | |
| Week 2 | 1.00 | 1.19 (0.51,2.77) | 1.77 (0.78,4.05) | 0.93 (0.34,2.54) |
| Week 3 | 1.00 | 0.97 (0.45,2.07) | 1.02 (0.48,2.18) | 1.56 (0.64,3.77) |
| Week 4 | 1.00 | 1.01 (0.49,2.11) | 1.09 (0.53,2.24) | 0.65 (0.25,1.68) |
| Week 5 | 1.00 | 0.73 (0.34,1.55) | 1.23 (0.60,2.52) | 1.58 (0.66,3.78) |
| Week 6 | 1.00 | 1.19 (0.56,2.54) | 0.96 (0.45,2.04) | 0.89 (0.35,2.31) |
| Week 7 | 1.00 | 1.78 (0.78,4.08) | 1.22 (0.53,2.82) | 1.28 (0.49,3.38) |
| Week 8 | 1.00 | 0.45 (0.23,0.85) | 0.46 (0.25,0.88) | 0.93 (0.43,2.02) |

| Exposure and Defect | <10th centile | 10-<50th centile | 50-<90th centile | ≥90th centile |
|---|---|---|---|---|
| TAPVR[c] | | | | |
| Week 2 | 1.00 | 1.05 (0.46,2.42) | 1.57 (0.70,3.53) | 0.88 (0.33,2.36) |
| Week 3 | 1.00 | 0.82 (0.38,1.76) | 0.92 (0.43,1.95) | 1.52 (0.64,3.63) |
| Week 4 | 1.00 | 0.85 (0.41,1.78) | 1.06 (0.52,2.17) | 0.65 (0.25,1.67) |
| Week 5 | 1.00 | 0.69 (0.33,1.47) | 1.07 (0.52,2.20) | 1.58 (0.67,3.74) |
| Week 6 | 1.00 | 1.19 (0.57,2.52) | 0.84 (0.39,1.80) | 0.76 (0.29,1.99) |
| Week 7 | 1.00 | 1.64 (0.72,3.70) | 1.17 (0.51,2.67) | 1.12 (0.42,2.93) |
| Week 8 | 1.00 | 0.48 (0.25,0.92) | 0.47 (0.24,0.91) | 0.99 (0.45,2.17) |
| AVSD[b] | | | | |
| Week 2 | 1.00 | 0.98 (0.38,2.53) | 0.97 (0.38,2.51) | 1.76 (0.61,5.08) |
| Week 3 | 1.00 | 0.74 (0.28,1.95) | 1.83 (0.73,4.55) | 1.19 (0.23,6.12) |
| Week 4 | 1.00 | 1.52 (0.52,4.39) | 1.30 (0.46,3.69) | 0.75 (0.19,2.96) |
| Week 5 | 1.00 | 0.75 (0.29,1.95) | 1.63 (0.65,4.05) | 1.18 (0.23,6.11) |
| Week 6 | 1.00 | 1.46 (0.56,3.79) | 0.95 (0.36,2.50) | 0.85 (0.25,2.91) |
| Week 7 | 1.00 | 1.25 (0.54,2.90) | 0.58 (0.23,1.46) | 1.92 (0.71,5.24) |
| Week 8 | 1.00 | 0.52 (0.23,1.20) | 0.79 (0.36,1.72) | 0.60 (0.18,1.94) |
| RVOTO[b] | | | | |
| Week 2 | 1.00 | 1.10 (0.72,1.67) | 1.16 (0.76,1.79) | 0.83 (0.47,1.45) |
| Week 3 | 1.00 | 1.48 (0.95,2.30) | 1.28 (0.81,2.02) | 1.46 (0.83,2.55) |
| Week 4 | 1.00 | 1.12 (0.75,1.69) | 0.80 (0.52,1.22) | 1.04 (0.61,1.76) |
| Week 5 | 1.00 | 0.81 (0.55,1.18) | 0.84 (0.56,1.24) | 0.86 (0.51,1.45) |
| Week 6 | 1.00 | 0.97 (0.65,1.45) | 0.98 (0.65,1.48) | 0.94 (0.54,1.63) |
| Week 7 | 1.00 | 1.34 (0.86,2.10) | 1.49 (0.94,2.34) | 1.60 (0.92,2.81) |
| Week 8 | 1.00 | 0.86 (0.56,1.31) | 1.19 (0.78,1.82) | 1.27 (0.75,2.15) |
| Pulmonary/tricuspid atresia[c] | | | | |
| Week 2 | 1.00 | 0.88 (0.44,1.75) | 0.9 (0.45,1.81) | 1.08 (0.46,2.57) |
| Week 3 | 1.00 | 1.50 (0.69,3.23) | 1.35 (0.62,2.93) | 0.79 (0.28,2.20) |
| Week 4 | 1.00 | 0.98 (0.53,1.83) | 0.50 (0.25,0.99) | 0.45 (0.17,1.21) |
| Week 5 | 1.00 | 1.27 (0.61,2.64) | 1.07 (0.50,2.25) | 1.23 (0.49,3.10) |
| Week 6 | 1.00 | 1.09 (0.52,2.29) | 1.11 (0.53,2.33) | 0.88 (0.34,2.27) |
| Week 7 | 1.00 | 0.82 (0.40,1.65) | 1.24 (0.62,2.46) | 1.25 (0.52,3.01) |
| Week 8 | 1.00 | 0.90 (0.44,1.84) | 0.83 (0.41,1.70) | 1.16 (0.49,2.74) |
| PVS[c] | | | | |
| Week 2 | 1.00 | 1.25 (0.77,2.04) | 1.30 (0.79,2.14) | 0.76 (0.39,1.46) |
| Week 3 | 1.00 | 1.34 (0.82,2.17) | 1.14 (0.70,1.88) | 1.40 (0.76,2.58) |
| Week 4 | 1.00 | 1.35 (0.82,2.20) | 1.02 (0.61,1.69) | 1.40 (0.76,2.56) |
| Week 5 | 1.00 | 0.70 (0.46,1.07) | 0.80 (0.52,1.23) | 0.80 (0.44,1.42) |
| Week 6 | 1.00 | 1.07 (0.67,1.71) | 1.11 (0.69,1.78) | 1.15 (0.62,2.12) |
| Week 7 | 1.00 | 1.45 (0.88,2.41) | 1.41 (0.85,2.37) | 1.57 (0.84,2.95) |
| Week 8 | 1.00 | 0.83 (0.51,1.36) | 1.27 (0.79,2.05) | 1.43 (0.80,2.57) |
| Septal[b] | | | | |
| Week 2 | 1.00 | 0.87 (0.66,1.16) | 0.90 (0.67,1.21) | 0.75 (0.50,1.12) |
| Week 3 | 1.00 | 1.18 (0.88,1.57) | 1.03 (0.76,1.39) | 1.00 (0.66,1.49) |

| Exposure and Defect | <10th centile | 10-<50th centile | 50-<90th centile | ≥90th centile |
|---|---|---|---|---|
| Week 4 | 1.00 | 1.02 (0.76,1.35) | 0.98 (0.73,1.33) | 0.86 (0.57,1.29) |
| Week 5 | 1.00 | 1.20 (0.88,1.61) | 1.31 (0.96,1.78) | 1.46 (0.98,2.19) |
| Week 6 | 1.00 | 0.98 (0.74,1.29) | 0.90 (0.67,1.20) | 0.92 (0.62,1.38) |
| Week 7 | 1.00 | 0.94 (0.71,1.24) | 0.95 (0.71,1.27) | 0.88 (0.59,1.31) |
| Week 8 | 1.00 | 0.96 (0.72,1.28) | 1.05 (0.78,1.41) | 1.05 (0.71,1.55) |
| $VSD_{pm}$[c] | | | | |
| Week 2 | 1.00 | 0.84 (0.57,1.24) | 0.92 (0.62,1.36) | 0.87 (0.52,1.47) |
| Week 3 | 1.00 | 1.30 (0.86,1.96) | 1.16 (0.76,1.77) | 0.86 (0.48,1.52) |
| Week 4 | 1.00 | 1.13 (0.75,1.71) | 1.06 (0.70,1.62) | 0.90 (0.52,1.57) |
| Week 5 | 1.00 | 1.30 (0.84,2.00) | 1.30 (0.84,2.03) | 1.76 (1.02,3.03) |
| Week 6 | 1.00 | 0.87 (0.59,1.27) | 0.81 (0.55,1.20) | 0.75 (0.44,1.29) |
| Week 7 | 1.00 | 0.96 (0.65,1.42) | 0.91 (0.61,1.35) | 0.98 (0.58,1.66) |
| Week 8 | 1.00 | 1.01 (0.66,1.56) | 1.48 (0.96,2.28) | 1.24 (0.72,2.16) |
| ASD[c] | | | | |
| Week 2 | 1.00 | 0.98 (0.67,1.41) | 0.98 (0.67,1.44) | 0.66 (0.38,1.15) |
| Week 3 | 1.00 | 1.16 (0.80,1.68) | 1.02 (0.69,1.50) | 1.13 (0.67,1.91) |
| Week 4 | 1.00 | 0.99 (0.68,1.42) | 0.95 (0.65,1.39) | 0.80 (0.48,1.36) |
| Week 5 | 1.00 | 1.04 (0.71,1.51) | 1.18 (0.80,1.73) | 1.13 (0.67,1.91) |
| Week 6 | 1.00 | 1.21 (0.83,1.76) | 1.03 (0.70,1.53) | 1.23 (0.73,2.08) |
| Week 7 | 1.00 | 1.00 (0.70,1.44) | 1.02 (0.70,1.49) | 0.91 (0.53,1.54) |
| Week 8 | 1.00 | 0.99 (0.69,1.43) | 0.90 (0.62,1.32) | 0.99 (0.60,1.64) |
| **$PM_{2.5}$ (μg/$m^3$)** | | | | |
| Week 2 | <6.0 | 6.0-<11.6 | 11.6-<22.6 | ≥22.6 |
| Week 3 | <6.2 | 6.2-<11.7 | 11.7-<22.5 | ≥22.5 |
| Week 4 | <5.9 | 5.9-<11.6 | 11.6-<22.1 | ≥22.1 |
| Week 5 | <5.9 | 5.9-<11.6 | 11.6-<22.1 | ≥22.1 |
| Week 6 | <6.0 | 6.0-<11.6 | 11.6-<22.6 | ≥22.6 |
| Week 7 | <6.0 | 6.0-<11.6 | 11.6-<22.5 | ≥22.5 |
| Week 8 | <5.9 | 5.9-<11.4 | 11.4-<22.2 | ≥22.2 |
| LVOTO[b] [OR (95% CI)] | | | | |
| Week 2 | 1.00 | 1.11 (0.79,1.55) | 1.10 (0.77,1.57) | 1.48 (0.94,2.33) |
| Week 3 | 1.00 | 0.69 (0.51,0.94) | 0.54 (0.38,0.75) | 0.52 (0.33,0.83) |
| Week 4 | 1.00 | 1.37 (0.96,1.96) | 1.28 (0.87,1.87) | 1.56 (0.97,2.51) |
| Week 5 | 1.00 | 1.01 (0.73,1.41) | 0.95 (0.67,1.36) | 1.12 (0.70,1.79) |
| Week 6 | 1.00 | 1.04 (0.74,1.47) | 1.09 (0.75,1.57) | 0.95 (0.58,1.55) |
| Week 7 | 1.00 | 1.16 (0.82,1.64) | 1.20 (0.84,1.73) | 1.23 (0.75,2.01) |
| Week 8 | 1.00 | 1.18 (0.85,1.64) | 1.01 (0.71,1.44) | 0.95 (0.59,1.53) |
| Aortic stenosis[c] | | | | |
| Week 2 | 1.00 | 1.09 (0.61,1.94) | 1.18 (0.65,2.16) | 1.70 (0.78,3.69) |
| Week 3 | 1.00 | 0.55 (0.33,0.89) | 0.42 (0.24,0.73) | 0.39 (0.17,0.88) |
| Week 4 | 1.00 | 1.42 (0.77,2.62) | 1.30 (0.68,2.48) | 1.67 (0.75,3.72) |
| Week 5 | 1.00 | 0.83 (0.48,1.45) | 1.28 (0.72,2.26) | 1.00 (0.43,2.31) |
| Week 6 | 1.00 | 1.59 (0.86,2.93) | 1.06 (0.55,2.05) | 0.81 (0.33,2.00) |

| Exposure and Defect | <10th centile | 10-<50th centile | 50-<90th centile | ≥90th centile |
|---|---|---|---|---|
| Week 7 | 1.00 | 1.03 (0.60,1.76) | 0.87 (0.48,1.56) | 0.95 (0.41,2.21) |
| Week 8 | 1.00 | 1.18 (0.67,2.06) | 0.95 (0.52,1.73) | 1.04 (0.46,2.35) |
| COA[c] | | | | |
| Week 2 | 1.00 | 1.18 (0.73,1.92) | 1.19 (0.71,1.98) | 1.40 (0.73,2.69) |
| Week 3 | 1.00 | 0.82 (0.53,1.27) | 0.59 (0.36,0.95) | 0.52 (0.27,1.01) |
| Week 4 | 1.00 | 1.37 (0.82,2.29) | 1.45 (0.85,2.48) | 1.61 (0.82,3.14) |
| Week 5 | 1.00 | 0.99 (0.63,1.57) | 0.90 (0.55,1.46) | 0.98 (0.51,1.89) |
| Week 6 | 1.00 | 0.71 (0.45,1.13) | 0.92 (0.57,1.49) | 0.96 (0.50,1.82) |
| Week 7 | 1.00 | 1.23 (0.76,2.01) | 1.20 (0.72,2.00) | 1.15 (0.58,2.28) |
| Week 8 | 1.00 | 1.47 (0.90,2.40) | 1.09 (0.64,1.83) | 1.15 (0.59,2.24) |
| HLHS[c] | | | | |
| Week 2 | 1.00 | 1.01 (0.62,1.64) | 0.87 (0.52,1.46) | 1.18 (0.62,2.25) |
| Week 3 | 1.00 | 0.79 (0.49,1.27) | 0.71 (0.43,1.18) | 0.79 (0.41,1.51) |
| Week 4 | 1.00 | 1.11 (0.67,1.84) | 0.92 (0.54,1.56) | 1.13 (0.58,2.21) |
| Week 5 | 1.00 | 1.24 (0.75,2.05) | 0.87 (0.50,1.51) | 1.52 (0.79,2.94) |
| Week 6 | 1.00 | 1.11 (0.65,1.88) | 1.22 (0.71,2.12) | 0.91 (0.44,1.85) |
| Week 7 | 1.00 | 1.25 (0.73,2.15) | 1.51 (0.86,2.63) | 1.62 (0.81,3.27) |
| Week 8 | 1.00 | 0.85 (0.53,1.37) | 0.93 (0.57,1.54) | 0.72 (0.36,1.42) |
| Conotruncal[b] | | | | |
| Week 2 | 1.00 | 1.39 (0.98,1.97) | 1.29 (0.90,1.86) | 1.50 (0.96,2.35) |
| Week 3 | 1.00 | 0.80 (0.59,1.09) | 0.72 (0.52,0.99) | 0.58 (0.38,0.9) |
| Week 4 | 1.00 | 0.95 (0.69,1.32) | 1.10 (0.78,1.55) | 1.11 (0.71,1.71) |
| Week 5 | 1.00 | 1.01 (0.73,1.39) | 0.91 (0.65,1.28) | 0.91 (0.58,1.42) |
| Week 6 | 1.00 | 1.02 (0.73,1.42) | 1.00 (0.70,1.42) | 0.89 (0.57,1.41) |
| Week 7 | 1.00 | 1.28 (0.91,1.82) | 1.40 (0.97,2.01) | 1.52 (0.97,2.40) |
| Week 8 | 1.00 | 0.90 (0.65,1.25) | 1.19 (0.85,1.66) | 1.23 (0.80,1.87) |
| d-TGA[c] | | | | |
| Week 2 | 1.00 | 1.20 (0.73,1.97) | 1.09 (0.65,1.83) | 0.96 (0.49,1.89) |
| Week 3 | 1.00 | 0.79 (0.50,1.26) | 0.77 (0.47,1.26) | 0.47 (0.23,0.95) |
| Week 4 | 1.00 | 1.04 (0.62,1.73) | 1.43 (0.85,2.43) | 1.31 (0.67,2.57) |
| Week 5 | 1.00 | 0.92 (0.57,1.47) | 0.92 (0.56,1.51) | 0.97 (0.50,1.87) |
| Week 6 | 1.00 | 0.82 (0.50,1.34) | 0.88 (0.53,1.46) | 1.15 (0.61,2.17) |
| Week 7 | 1.00 | 1.18 (0.71,1.95) | 1.19 (0.70,2.01) | 1.32 (0.68,2.58) |
| Week 8 | 1.00 | 1.28 (0.76,2.16) | 1.33 (0.77,2.28) | 1.17 (0.59,2.30) |
| TOF[c] | | | | |
| Week 2 | 1.00 | 1.64 (1.02,2.63) | 1.41 (0.87,2.31) | 1.96 (1.11,3.46) |
| Week 3 | 1.00 | 0.78 (0.53,1.16) | 0.70 (0.46,1.06) | 0.73 (0.43,1.24) |
| Week 4 | 1.00 | 0.87 (0.58,1.31) | 0.96 (0.63,1.46) | 1.06 (0.63,1.81) |
| Week 5 | 1.00 | 1.11 (0.73,1.68) | 1.05 (0.68,1.63) | 0.90 (0.51,1.58) |
| Week 6 | 1.00 | 1.19 (0.77,1.84) | 1.09 (0.69,1.72) | 0.79 (0.44,1.41) |
| Week 7 | 1.00 | 1.16 (0.75,1.80) | 1.41 (0.90,2.20) | 1.37 (0.79,2.40) |
| Week 8 | 1.00 | 0.82 (0.54,1.23) | 1.14 (0.75,1.74) | 1.42 (0.85,2.37) |
| Other conotruncals[c,d] | | | | |
| Week 2 | 1.00 | 0.99 (0.50,1.95) | 1.23 (0.62,2.45) | 1.13 (0.46,2.80) |

| Exposure and Defect | <10th centile | 10-<50th centile | 50-<90th centile | ≥90th centile |
|---|---|---|---|---|
| Week 3 | 1.00 | 0.95 (0.50,1.79) | 0.73 (0.37,1.43) | 0.36 (0.13,1.01) |
| Week 4 | 1.00 | 1.17 (0.59,2.29) | 1.09 (0.54,2.19) | 0.83 (0.32,2.14) |
| Week 5 | 1.00 | 1.13 (0.59,2.16) | 0.65 (0.32,1.31) | 1.29 (0.55,3.02) |
| Week 6 | 1.00 | 0.93 (0.47,1.83) | 0.97 (0.48,1.94) | 0.82 (0.33,2.03) |
| Week 7 | 1.00 | 1.54 (0.70,3.41) | 1.27 (0.57,2.84) | 1.96 (0.78,4.90) |
| Week 8 | 1.00 | 0.60 (0.32,1.14) | 1.07 (0.57,1.99) | 0.63 (0.25,1.59) |
| APVR[b] | | | | |
| Week 2 | 1.00 | 1.57 (0.79,3.16) | 1.14 (0.55,2.35) | 1.79 (0.76,4.22) |
| Week 3 | 1.00 | 0.61 (0.34,1.09) | 0.65 (0.35,1.22) | 0.77 (0.34,1.74) |
| Week 4 | 1.00 | 1.32 (0.67,2.59) | 1.28 (0.63,2.58) | 1.28 (0.53,3.08) |
| Week 5 | 1.00 | 1.46 (0.70,3.04) | 1.78 (0.85,3.75) | 1.60 (0.64,3.98) |
| Week 6 | 1.00 | 0.79 (0.44,1.42) | 0.48 (0.25,0.94) | 1.00 (0.45,2.24) |
| Week 7 | 1.00 | 1.27 (0.68,2.39) | 0.97 (0.49,1.90) | 1.02 (0.42,2.49) |
| Week 8 | 1.00 | 1.36 (0.70,2.66) | 1.03 (0.51,2.10) | 1.33 (0.56,3.16) |
| TAPVR[c] | | | | |
| Week 2 | 1.00 | 1.43 (0.72,2.86) | 0.98 (0.47,2.04) | 1.67 (0.71,3.95) |
| Week 3 | 1.00 | 0.50 (0.27,0.91) | 0.62 (0.33,1.16) | 0.72 (0.31,1.64) |
| Week 4 | 1.00 | 1.11 (0.56,2.18) | 1.17 (0.58,2.35) | 1.39 (0.59,3.30) |
| Week 5 | 1.00 | 1.53 (0.68,3.40) | 1.70 (0.76,3.79) | 1.91 (0.75,4.84) |
| Week 6 | 1.00 | 0.88 (0.47,1.62) | 0.54 (0.27,1.07) | 0.93 (0.40,2.15) |
| Week 7 | 1.00 | 1.47 (0.75,2.87) | 1.14 (0.56,2.31) | 0.87 (0.33,2.27) |
| Week 8 | 1.00 | 1.54 (0.75,3.15) | 1.05 (0.50,2.23) | 1.27 (0.51,3.14) |
| AVSD[b] | | | | |
| Week 2 | 1.00 | 1.27 (0.54,2.98) | 0.82 (0.34,1.99) | 3.43 (1.36,8.66) |
| Week 3 | 1.00 | 0.71 (0.31,1.65) | 1.44 (0.65,3.22) | 0.69 (0.24,2.00) |
| Week 4 | 1.00 | 1.56 (0.67,3.64) | 1.23 (0.52,2.92) | 1.19 (0.42,3.36) |
| Week 5 | 1.00 | 1.37 (0.61,3.07) | 1.19 (0.52,2.72) | 1.29 (0.47,3.57) |
| Week 6 | 1.00 | 1.23 (0.57,2.67) | 0.89 (0.40,1.99) | 0.48 (0.15,1.57) |
| Week 7 | 1.00 | 1.14 (0.48,2.71) | 1.32 (0.56,3.10) | 2.45 (0.92,6.50) |
| Week 8 | 1.00 | 0.96 (0.47,1.98) | 0.67 (0.31,1.44) | 1.07 (0.41,2.75) |
| RVOTO[b] | | | | |
| Week 2 | 1.00 | 1.20 (0.84,1.71) | 0.92 (0.63,1.35) | 0.95 (0.57,1.57) |
| Week 3 | 1.00 | 0.74 (0.53,1.04) | 0.76 (0.53,1.09) | 0.71 (0.43,1.16) |
| Week 4 | 1.00 | 0.96 (0.68,1.37) | 0.98 (0.67,1.43) | 0.94 (0.57,1.54) |
| Week 5 | 1.00 | 1.09 (0.75,1.56) | 1.17 (0.79,1.72) | 1.74 (1.07,2.83) |
| Week 6 | 1.00 | 0.82 (0.58,1.17) | 0.84 (0.58,1.22) | 0.76 (0.46,1.26) |
| Week 7 | 1.00 | 1.05 (0.74,1.50) | 1.00 (0.69,1.45) | 0.81 (0.48,1.37) |
| Week 8 | 1.00 | 1.15 (0.80,1.65) | 1.06 (0.72,1.56) | 1.38 (0.85,2.24) |
| Pulmonary/tricuspid atresia[c] | | | | |
| Week 2 | 1.00 | 1.60 (0.83,3.09) | 0.99 (0.48,2.00) | 1.12 (0.46,2.70) |
| Week 3 | 1.00 | 0.97 (0.52,1.83) | 0.73 (0.37,1.44) | 0.83 (0.35,1.95) |
| Week 4 | 1.00 | 1.09 (0.59,2.02) | 0.80 (0.41,1.57) | 1.06 (0.45,2.49) |
| Week 5 | 1.00 | 1.23 (0.64,2.35) | 1.20 (0.60,2.39) | 1.53 (0.65,3.60) |

| Exposure and Defect | <10th centile | 10-<50th centile | 50-<90th centile | ≥90th centile |
|---|---|---|---|---|
| Week 6 | 1.00 | 0.73 (0.40,1.31) | 0.66 (0.35,1.25) | 0.95 (0.41,2.18) |
| Week 7 | 1.00 | 0.95 (0.52,1.73) | 1.02 (0.54,1.92) | 0.83 (0.34,2.03) |
| Week 8 | 1.00 | 0.59 (0.34,1.01) | 0.45 (0.24,0.83) | 0.70 (0.31,1.57) |
| PVS[c] | | | | |
| Week 2 | 1.00 | 1.17 (0.78,1.75) | 0.89 (0.58,1.37) | 0.94 (0.54,1.65) |
| Week 3 | 1.00 | 0.65 (0.44,0.95) | 0.70 (0.47,1.05) | 0.60 (0.35,1.05) |
| Week 4 | 1.00 | 0.90 (0.60,1.35) | 1.05 (0.69,1.61) | 0.88 (0.50,1.54) |
| Week 5 | 1.00 | 1.00 (0.66,1.51) | 1.12 (0.72,1.72) | 1.83 (1.08,3.12) |
| Week 6 | 1.00 | 0.97 (0.64,1.47) | 0.97 (0.63,1.49) | 0.82 (0.46,1.46) |
| Week 7 | 1.00 | 1.12 (0.74,1.69) | 1.10 (0.72,1.70) | 0.91 (0.50,1.64) |
| Week 8 | 1.00 | 1.43 (0.91,2.24) | 1.37 (0.86,2.19) | 1.71 (0.97,3.00) |
| Septal[b] | | | | |
| Week 2 | 1.00 | 1.02 (0.80,1.31) | 0.92 (0.71,1.2) | 0.60 (0.40,0.90) |
| Week 3 | 1.00 | 0.86 (0.68,1.10) | 0.78 (0.60,1.02) | 0.84 (0.58,1.23) |
| Week 4 | 1.00 | 1.00 (0.78,1.30) | 0.92 (0.70,1.22) | 0.95 (0.65,1.39) |
| Week 5 | 1.00 | 1.12 (0.87,1.45) | 0.95 (0.72,1.26) | 1.20 (0.82,1.75) |
| Week 6 | 1.00 | 0.97 (0.76,1.26) | 0.89 (0.68,1.18) | 0.81 (0.55,1.20) |
| Week 7 | 1.00 | 1.17 (0.90,1.51) | 1.14 (0.86,1.51) | 0.98 (0.65,1.47) |
| Week 8 | 1.00 | 0.88 (0.69,1.13) | 0.99 (0.76,1.29) | 1.02 (0.71,1.48) |
| $VSD_{pm}$[c] | | | | |
| Week 2 | 1.00 | 0.97 (0.68,1.38) | 1.05 (0.72,1.51) | 0.60 (0.34,1.04) |
| Week 3 | 1.00 | 1.06 (0.74,1.51) | 0.88 (0.60,1.30) | 0.73 (0.43,1.24) |
| Week 4 | 1.00 | 1.02 (0.70,1.47) | 1.06 (0.72,1.56) | 1.14 (0.68,1.89) |
| Week 5 | 1.00 | 1.34 (0.92,1.95) | 1.00 (0.67,1.50) | 1.34 (0.80,2.23) |
| Week 6 | 1.00 | 0.88 (0.61,1.26) | 0.95 (0.65,1.38) | 0.72 (0.42,1.23) |
| Week 7 | 1.00 | 1.21 (0.83,1.75) | 1.13 (0.77,1.68) | 0.90 (0.52,1.56) |
| Week 8 | 1.00 | 0.93 (0.65,1.32) | 1.08 (0.74,1.56) | 1.25 (0.77,2.04) |
| ASD[c] | | | | |
| Week 2 | 1.00 | 1.07 (0.80,1.45) | 0.84 (0.60,1.17) | 0.66 (0.40,1.11) |
| Week 3 | 1.00 | 0.74 (0.55,0.98) | 0.72 (0.52,0.99) | 0.95 (0.60,1.49) |
| Week 4 | 1.00 | 1.01 (0.75,1.37) | 0.83 (0.59,1.16) | 0.83 (0.51,1.35) |
| Week 5 | 1.00 | 0.97 (0.71,1.31) | 0.93 (0.67,1.31) | 1.13 (0.71,1.82) |
| Week 6 | 1.00 | 1.05 (0.78,1.43) | 0.84 (0.60,1.18) | 0.87 (0.53,1.42) |
| Week 7 | 1.00 | 1.19 (0.87,1.64) | 1.19 (0.84,1.68) | 1.12 (0.67,1.86) |
| Week 8 | 1.00 | 0.87 (0.65,1.18) | 0.96 (0.69,1.33) | 0.87 (0.54,1.40) |
| **$SO_2$ (ppb)[f]** | | | | |
| Week 2 | <2.73 | 2.73-<8.89 | 8.89-<21.7 | ≥21.7 |
| Week 3 | <2.86 | 2.86-<8.57 | 8.57-<21.6 | ≥21.6 |
| Week 4 | <2.71 | 2.71-<8.71 | 8.57-<22.1 | ≥22.1 |
| Week 5 | <2.71 | 2.71-<8.71 | 8.57-<22.1 | ≥22.1 |
| Week 6 | <2.71 | 2.71-<8.71 | 8.71-<21.6 | ≥21.6 |
| Week 7 | <2.71 | 2.71-<8.57 | 8.57-<22.0 | ≥22.0 |
| Week 8 | <2.71 | 2.71-<8.71 | 8.71-<21.9 | ≥21.9 |

| Exposure and Defect | <10th centile | 10-<50th centile | 50-<90th centile | ≥90th centile |
|---|---|---|---|---|
| LVOTO[b] [OR (95% CI)] | | | | |
| Week 2 | 1.00 | 0.79 (0.52,1.20) | 0.65 (0.41,1.02) | 0.70 (0.40,1.24) |
| Week 3 | 1.00 | 1.11 (0.69,1.78) | 1.42 (0.86,2.36) | 1.66 (0.91,3.05) |
| Week 4 | 1.00 | 1.01 (0.63,1.61) | 1.36 (0.82,2.24) | 0.71 (0.37,1.35) |
| Week 5 | 1.00 | 1.02 (0.64,1.63) | 1.04 (0.63,1.72) | 1.16 (0.64,2.12) |
| Week 6 | 1.00 | 1.30 (0.79,2.13) | 1.03 (0.61,1.74) | 1.02 (0.54,1.91) |
| Week 7 | 1.00 | 1.28 (0.78,2.09) | 1.38 (0.82,2.33) | 0.94 (0.50,1.80) |
| Week 8 | 1.00 | 1.57 (0.98,2.53) | 1.39 (0.84,2.32) | 1.70 (0.93,3.10) |
| Aortic stenosis[c] | | | | |
| Week 2 | 1.00 | 0.37 (0.19,0.74) | 0.74 (0.37,1.50) | 0.68 (0.27,1.73) |
| Week 3 | 1.00 | 1.49 (0.70,3.18) | 1.23 (0.55,2.76) | 1.18 (0.45,3.15) |
| Week 4 | 1.00 | 0.84 (0.40,1.77) | 1.43 (0.66,3.10) | 0.73 (0.27,2.02) |
| Week 5 | 1.00 | 0.69 (0.34,1.39) | 0.63 (0.29,1.34) | 1.11 (0.45,2.72) |
| Week 6 | 1.00 | 1.43 (0.67,3.07) | 0.78 (0.35,1.76) | 1.30 (0.51,3.33) |
| Week 7 | 1.00 | 1.23 (0.59,2.53) | 0.98 (0.45,2.14) | 0.52 (0.18,1.46) |
| Week 8 | 1.00 | 1.45 (0.67,3.11) | 1.26 (0.56,2.82) | 1.83 (0.71,4.71) |
| COA[c] | | | | |
| Week 2 | 1.00 | 1.03 (0.57,1.85) | 0.68 (0.37,1.27) | 0.72 (0.34,1.52) |
| Week 3 | 1.00 | 0.97 (0.51,1.86) | 1.74 (0.90,3.39) | 1.96 (0.90,4.25) |
| Week 4 | 1.00 | 1.38 (0.71,2.67) | 1.63 (0.82,3.23) | 0.93 (0.41,2.14) |
| Week 5 | 1.00 | 1.06 (0.57,1.97) | 1.01 (0.53,1.94) | 1.08 (0.50,2.32) |
| Week 6 | 1.00 | 1.39 (0.72,2.69) | 1.05 (0.53,2.08) | 1.32 (0.60,2.90) |
| Week 7 | 1.00 | 1.37 (0.69,2.72) | 1.49 (0.74,3.02) | 1.30 (0.57,2.93) |
| Week 8 | 1.00 | 1.08 (0.59,1.97) | 1.35 (0.72,2.51) | 1.31 (0.62,2.78) |
| HLHS[c] | | | | |
| Week 2 | 1.00 | 0.96 (0.53,1.73) | 0.71 (0.38,1.33) | 0.95 (0.44,2.07) |
| Week 3 | 1.00 | 1.05 (0.57,1.95) | 1.09 (0.57,2.08) | 1.35 (0.60,3.02) |
| Week 4 | 1.00 | 0.83 (0.46,1.51) | 1.14 (0.61,2.15) | 0.60 (0.25,1.44) |
| Week 5 | 1.00 | 1.35 (0.68,2.66) | 1.53 (0.75,3.10) | 1.29 (0.55,3.04) |
| Week 6 | 1.00 | 1.24 (0.65,2.37) | 1.27 (0.65,2.50) | 0.68 (0.27,1.71) |
| Week 7 | 1.00 | 1.31 (0.67,2.53) | 1.53 (0.77,3.04) | 0.87 (0.36,2.13) |
| Week 8 | 1.00 | 1.99 (1.01,3.95) | 1.18 (0.57,2.42) | 1.74 (0.76,3.98) |
| Conotruncal[b] | | | | |
| Week 2 | 1.00 | 0.83 (0.55,1.25) | 1.12 (0.72,1.73) | 1.00 (0.58,1.72) |
| Week 3 | 1.00 | 0.89 (0.58,1.35) | 0.99 (0.63,1.55) | 1.10 (0.64,1.89) |
| Week 4 | 1.00 | 1.33 (0.86,2.05) | 1.06 (0.66,1.69) | 0.97 (0.55,1.72) |
| Week 5 | 1.00 | 0.80 (0.54,1.20) | 0.77 (0.49,1.19) | 0.77 (0.45,1.31) |
| Week 6 | 1.00 | 1.03 (0.66,1.59) | 1.17 (0.73,1.88) | 1.15 (0.65,2.02) |
| Week 7 | 1.00 | 1.09 (0.71,1.67) | 1.18 (0.74,1.88) | 0.94 (0.53,1.67) |
| Week 8 | 1.00 | 1.23 (0.81,1.86) | 1.24 (0.79,1.94) | 1.14 (0.66,1.96) |
| d-TGA[c] | | | | |
| Week 2 | 1.00 | 0.81 (0.44,1.47) | 1.25 (0.67,2.32) | 0.86 (0.40,1.86) |
| Week 3 | 1.00 | 1.21 (0.67,2.22) | 0.94 (0.50,1.79) | 0.96 (0.45,2.06) |

| Exposure and Defect | <10th centile | 10-<50th centile | 50-<90th centile | ≥90th centile |
|---|---|---|---|---|
| Week 4 | 1.00 | 0.90 (0.49,1.66) | 1.09 (0.57,2.07) | 1.16 (0.54,2.50) |
| Week 5 | 1.00 | 0.86 (0.48,1.54) | 0.74 (0.40,1.39) | 0.95 (0.45,1.98) |
| Week 6 | 1.00 | 1.00 (0.54,1.87) | 1.08 (0.56,2.07) | 1.31 (0.61,2.81) |
| Week 7 | 1.00 | 0.93 (0.50,1.71) | 1.19 (0.63,2.27) | 0.98 (0.45,2.12) |
| Week 8 | 1.00 | 1.13 (0.63,2.03) | 1.15 (0.62,2.13) | 0.86 (0.40,1.86) |
| TOF[c] | | | | |
| Week 2 | 1.00 | 0.84 (0.51,1.39) | 0.95 (0.56,1.62) | 0.99 (0.51,1.91) |
| Week 3 | 1.00 | 0.76 (0.46,1.26) | 1.06 (0.63,1.81) | 1.14 (0.59,2.20) |
| Week 4 | 1.00 | 1.43 (0.84,2.44) | 0.91 (0.51,1.61) | 0.94 (0.47,1.87) |
| Week 5 | 1.00 | 0.93 (0.56,1.53) | 0.86 (0.50,1.48) | 0.76 (0.39,1.49) |
| Week 6 | 1.00 | 1.00 (0.59,1.71) | 1.15 (0.66,2.03) | 0.91 (0.45,1.84) |
| Week 7 | 1.00 | 1.35 (0.78,2.33) | 1.40 (0.78,2.51) | 1.13 (0.56,2.31) |
| Week 8 | 1.00 | 1.17 (0.71,1.95) | 1.15 (0.67,1.98) | 1.29 (0.67,2.50) |
| Other conotruncals[c,d] | | | | |
| Week 2 | 1.00 | 0.56 (0.26,1.21) | 1.00 (0.46,2.14) | 0.96 (0.38,2.44) |
| Week 3 | 1.00 | 0.99 (0.45,2.19) | 1.16 (0.52,2.61) | 1.74 (0.68,4.42) |
| Week 4 | 1.00 | 1.82 (0.80,4.14) | 1.40 (0.60,3.29) | 0.53 (0.16,1.69) |
| Week 5 | 1.00 | 0.62 (0.29,1.29) | 0.91 (0.42,1.94) | 0.87 (0.34,2.27) |
| Week 6 | 1.00 | 1.05 (0.47,2.32) | 1.19 (0.53,2.68) | 1.30 (0.50,3.42) |
| Week 7 | 1.00 | 0.89 (0.43,1.86) | 0.78 (0.36,1.68) | 0.55 (0.20,1.54) |
| Week 8 | 1.00 | 1.49 (0.66,3.35) | 1.54 (0.67,3.51) | 1.15 (0.42,3.15) |
| APVR[b] | | | | |
| Week 2 | 1.00 | 0.87 (0.42,1.83) | 0.81 (0.38,1.77) | 0.66 (0.24,1.82) |
| Week 3 | 1.00 | 1.35 (0.60,3.02) | 1.34 (0.58,3.10) | 1.08 (0.38,3.04) |
| Week 4 | 1.00 | 1.40 (0.59,3.29) | 1.52 (0.64,3.63) | 1.05 (0.36,3.04) |
| Week 5 | 1.00 | 1.25 (0.55,2.83) | 1.66 (0.72,3.83) | 0.76 (0.25,2.36) |
| Week 6 | 1.00 | 1.28 (0.55,3.01) | 1.06 (0.45,2.54) | 2.34 (0.89,6.17) |
| Week 7 | 1.00 | 1.29 (0.58,2.89) | 0.90 (0.39,2.10) | 1.38 (0.52,3.64) |
| Week 8 | 1.00 | 1.96 (0.79,4.85) | 2.16 (0.87,5.37) | 1.39 (0.47,4.10) |
| TAPVR[c] | | | | |
| Week 2 | 1.00 | 0.89 (0.42,1.90) | 0.87 (0.40,1.91) | 0.76 (0.28,2.07) |
| Week 3 | 1.00 | 1.34 (0.60,2.99) | 1.34 (0.59,3.05) | 0.99 (0.35,2.84) |
| Week 4 | 1.00 | 1.32 (0.57,3.06) | 1.39 (0.59,3.28) | 1.06 (0.38,3.00) |
| Week 5 | 1.00 | 1.25 (0.54,2.93) | 1.74 (0.74,4.11) | 0.86 (0.28,2.61) |
| Week 6 | 1.00 | 1.19 (0.51,2.75) | 1.10 (0.47,2.58) | 1.95 (0.74,5.11) |
| Week 7 | 1.00 | 1.39 (0.60,3.21) | 0.99 (0.42,2.34) | 1.37 (0.51,3.66) |
| Week 8 | 1.00 | 1.72 (0.71,4.20) | 1.88 (0.77,4.57) | 1.38 (0.48,3.97) |
| RVOTO[b] | | | | |
| Week 2 | 1.00 | 0.81 (0.53,1.25) | 0.78 (0.49,1.24) | 1.05 (0.59,1.87) |
| Week 3 | 1.00 | 1.31 (0.82,2.10) | 1.07 (0.65,1.77) | 0.96 (0.51,1.79) |
| Week 4 | 1.00 | 0.98 (0.62,1.55) | 0.87 (0.53,1.43) | 0.95 (0.52,1.76) |
| Week 5 | 1.00 | 1.19 (0.73,1.94) | 1.45 (0.86,2.44) | 1.34 (0.71,2.51) |
| Week 6 | 1.00 | 1.16 (0.71,1.89) | 1.06 (0.63,1.78) | 1.09 (0.58,2.06) |
| Week 7 | 1.00 | 1.27 (0.78,2.07) | 1.31 (0.78,2.21) | 0.81 (0.42,1.56) |

| Exposure and Defect | <10th centile | 10-<50th centile | 50-<90th centile | ≥90th centile |
|---|---|---|---|---|
| Week 8 | 1.00 | 1.29 (0.81,2.04) | 1.40 (0.86,2.30) | 1.31 (0.71,2.41) |
| Pulmonary/tricuspid atresia[c] | | | | |
| Week 2 | 1.00 | 0.79 (0.37,1.67) | 1.32 (0.61,2.87) | 0.59 (0.20,1.74) |
| Week 3 | 1.00 | 1.61 (0.66,3.91) | 1.60 (0.65,3.91) | 1.48 (0.53,4.17) |
| Week 4 | 1.00 | 1.59 (0.72,3.52) | 0.87 (0.38,2.02) | 0.96 (0.35,2.62) |
| Week 5 | 1.00 | 0.77 (0.37,1.59) | 0.80 (0.37,1.73) | 1.23 (0.49,3.12) |
| Week 6 | 1.00 | 1.21 (0.55,2.67) | 1.00 (0.44,2.27) | 1.12 (0.42,3.02) |
| Week 7 | 1.00 | 0.83 (0.40,1.71) | 1.02 (0.47,2.20) | 0.52 (0.18,1.55) |
| Week 8 | 1.00 | 0.84 (0.42,1.66) | 0.74 (0.35,1.56) | 0.93 (0.36,2.38) |
| PVS[c] | | | | |
| Week 2 | 1.00 | 0.85 (0.53,1.36) | 0.70 (0.42,1.16) | 1.08 (0.58,2.01) |
| Week 3 | 1.00 | 1.14 (0.7,1.87) | 0.88 (0.52,1.49) | 0.81 (0.42,1.57) |
| Week 4 | 1.00 | 0.92 (0.56,1.51) | 0.88 (0.52,1.51) | 0.97 (0.50,1.87) |
| Week 5 | 1.00 | 1.33 (0.76,2.31) | 1.71 (0.95,3.06) | 1.39 (0.69,2.81) |
| Week 6 | 1.00 | 1.14 (0.67,1.95) | 1.07 (0.61,1.88) | 1.09 (0.55,2.17) |
| Week 7 | 1.00 | 1.53 (0.87,2.69) | 1.61 (0.89,2.91) | 0.96 (0.46,2.00) |
| Week 8 | 1.00 | 1.49 (0.87,2.53) | 1.68 (0.96,2.95) | 1.42 (0.71,2.81) |
| Septal[b] | | | | |
| Week 2 | 1.00 | 0.75 (0.55,1.01) | 0.63 (0.45,0.88) | 0.79 (0.52,1.20) |
| Week 3 | 1.00 | 0.90 (0.66,1.23) | 1.05 (0.74,1.49) | 1.24 (0.80,1.91) |
| Week 4 | 1.00 | 1.19 (0.86,1.64) | 1.03 (0.71,1.48) | 0.87 (0.55,1.38) |
| Week 5 | 1.00 | 1.20 (0.86,1.68) | 1.30 (0.90,1.88) | 1.34 (0.86,2.10) |
| Week 6 | 1.00 | 0.97 (0.70,1.34) | 1.11 (0.77,1.59) | 1.12 (0.71,1.75) |
| Week 7 | 1.00 | 1.10 (0.8,1.52) | 1.01 (0.70,1.46) | 0.89 (0.56,1.40) |
| Week 8 | 1.00 | 0.99 (0.72,1.34) | 1.07 (0.76,1.51) | 1.09 (0.71,1.68) |
| $VSD_{pm}$[c] | | | | |
| Week 2 | 1.00 | 0.76 (0.50,1.15) | 0.65 (0.41,1.02) | 1.08 (0.63,1.85) |
| Week 3 | 1.00 | 1.32 (0.82,2.11) | 1.50 (0.91,2.48) | 1.98 (1.10,3.56) |
| Week 4 | 1.00 | 1.36 (0.86,2.15) | 0.94 (0.57,1.55) | 0.77 (0.42,1.42) |
| Week 5 | 1.00 | 0.97 (0.61,1.53) | 1.23 (0.75,2.02) | 1.13 (0.62,2.04) |
| Week 6 | 1.00 | 0.95 (0.60,1.51) | 1.13 (0.69,1.85) | 1.02 (0.56,1.86) |
| Week 7 | 1.00 | 1.22 (0.77,1.93) | 1.03 (0.62,1.69) | 0.98 (0.54,1.78) |
| Week 8 | 1.00 | 0.82 (0.54,1.25) | 1.00 (0.63,1.57) | 0.93 (0.53,1.63) |
| ASD[c] | | | | |
| Week 2 | 1.00 | 0.70 (0.48,1.00) | 0.63 (0.41,0.95) | 0.64 (0.37,1.12) |
| Week 3 | 1.00 | 0.75 (0.52,1.10) | 0.84 (0.55,1.28) | 0.79 (0.44,1.39) |
| Week 4 | 1.00 | 1.01 (0.68,1.49) | 1.11 (0.71,1.73) | 0.90 (0.49,1.63) |
| Week 5 | 1.00 | 1.37 (0.92,2.05) | 1.23 (0.78,1.95) | 1.37 (0.77,2.44) |
| Week 6 | 1.00 | 0.95 (0.64,1.40) | 1.10 (0.71,1.71) | 1.07 (0.60,1.92) |
| Week 7 | 1.00 | 1.04 (0.70,1.53) | 1.05 (0.67,1.64) | 0.79 (0.43,1.43) |
| Week 8 | 1.00 | 1.13 (0.78,1.66) | 1.03 (0.67,1.59) | 1.10 (0.62,1.93) |

Abbreviations: APVR-anomalous pulmonary venous return; ASD-atrial septal defect; AVSD-atrioventricular septal defect; CO-carbon monoxide; COA-coarctation of the aorta; d-TGA-d-transposition of the great arteries; HLHS-hypoplastic left heart syndrome; LVOTO-left ventricular outflow tract obstructions; $NO_2$-nitrogen dioxide; $O_3$-ozone; $PM_{10}$-particulate matter less than 10 microns in diameter; $PM_{2.5}$-particulate matter less than 2.5 microns in diameter; PVS-pulmonary valve stenosis; RVOTO-right ventricular outflow tract obstructions; $SO_2$-sulfur dioxide; TAPVR-total anomalous pulmonary venous return; TOF-tetralogy of Fallot; $VSD_{pm}$-perimembranous ventricular septal defects.

[a]All results for the National Birth Defects Prevention Study population from 1997-2006, except for $PM_{2.5}$ which was 1999-2006 due to unavailability of monitoring data for $PM_{2.5}$ prior to 1999. [b]Estimates from a hierarchical regression model. First stage was polytomous logistic model with defect groupings and adjusted for maternal race, age, educational attainment, household income, maternal smoking status and alcohol consumption during early pregnancy, nativity, and site-specific heart defect ratio. Second stage was a linear model with indicator variables for defect grouping and level of exposure. Defect-groupings include all individual defects listed underneath with the following additions: LVOTO also includes IAA-Type A, APVR also includes partial APVR and RVOTO includes Ebstein's Anomaly. Those defects could not be analyzed individually due to limited sample size. [c]Estimates result from a hierarchical regression model, same as above but used individual defects as outcomes in first-stage model and included indicator variable for individual defect in second-stage model. [d]Other conotruncal includes common truncus, interrupted aortic-arch, type B and type not specified, double outlet right ventricle defects, and conoventricular septal defects. [e]For ozone, the three categories of exposure were $25^{th}$ to less than the $50^{th}$ centile, $50^{th}$ centile to less than the $75^{th}$ centile, at or greater than the $75^{th}$ centile, with the referent grouping being below the $25^{th}$ centile. [f]Due to small sample size and instable estimation, AVSD was removed from hierarchical analysis of $SO_2$ exposure.

**Table S5:** Adjusted odds ratios and 95% confidence intervals between congenital heart defects and pollutant factors[a] identified through principal components analysis, National Birth Defects Prevention Study, 1999-2006.

| Defect | Factor 1: <10th centile | Factor 1: 10th -<50th centile | Factor 1: 50th-<90th centile | Factor 1: ≥90th centile | Factor 2: <10th centile | Factor 2: 10th -<50th centile | Factor 2: 50th-<90th centile | Factor 2: ≥90th centile | Factor 3: <10th centile | Factor 3: 10th -<50th centile | Factor 3: 50th-<90th centile | Factor 3: ≥90th centile |
|---|---|---|---|---|---|---|---|---|---|---|---|---|
| **LVOTO[b]** | 1.00 | 1.57 (0.93,2.63) | 1.62 (0.96,2.74) | 1.04 (0.51,2.10) | 1.00 | 1.42 (0.86,2.36) | 1.26 (0.75,2.12) | 1.33 (0.69,2.53) | 1.00 | 1.02 (0.58,1.77) | 1.01 (0.58,1.77) | 0.86 (0.44,1.70) |
| Aortic stenosis[c] | 1.00 | 2.31 (0.76,6.98) | 2.34 (0.76,7.15) | 1.88 (0.31,11.2) | 1.00 | 2.15 (0.72,6.45) | 1.89 (0.62,5.72) | 1.93 (0.50,7.36) | 1.00 | 1.58 (0.56,4.52) | 1.00 (0.35,2.90) | 0.69 (0.16,3.10) |
| COA[c] | 1.00 | 1.25 (0.61,2.57) | 1.47 (0.71,3.03) | 1.22 (0.49,3.02) | 1.00 | 0.99 (0.52,1.87) | 0.82 (0.42,1.59) | 1.37 (0.62,3.00) | 1.00 | 0.81 (0.38,1.73) | 1.28 (0.61,2.67) | 0.67 (0.26,1.72) |
| HLHS[c] | 1.00 | 1.59 (0.77,3.28) | 1.58 (0.75,3.30) | 1.21 (0.46,3.22) | 1.00 | 2.04 (0.88,4.73) | 1.94 (0.82,4.56) | 1.26 (0.44,3.63) | 1.00 | 1.10 (0.50,2.43) | 0.88 (0.40,1.96) | 1.15 (0.46,2.86) |
| **Conotruncal[b]** | 1.00 | 1.18 (0.75,1.84) | 1.35 (0.86,2.12) | 1.24 (0.71,2.17) | 1.00 | 1.11 (0.72,1.69) | 1.15 (0.74,1.77) | 1.19 (0.69,2.04) | 1.00 | 0.79 (0.48,1.28) | 1.05 (0.64,1.70) | 0.80 (0.44,1.44) |
| d-TGA[c] | 1.00 | 1.09 (0.57,2.10) | 1.17 (0.60,2.27) | 1.17 (0.52,2.66) | 1.00 | 1.04 (0.55,1.96) | 1.03 (0.54,1.99) | 1.29 (0.58,2.86) | 1.00 | 0.68 (0.32,1.44) | 1.15 (0.55,2.39) | 1.05 (0.45,2.43) |
| TOF[c] | 1.00 | 1.33 (0.74,2.40) | 1.64 (0.91,2.97) | 1.27 (0.60,2.69) | 1.00 | 1.15 (0.66,2.00) | 1.33 (0.76,2.33) | 1.27 (0.63,2.55) | 1.00 | 0.85 (0.45,1.61) | 1.01 (0.54,1.89) | 0.72 (0.34,1.56) |
| Other conotruncals[c,d] | 1.00 | 1.08 (0.44,2.63) | 1.08 (0.43,2.69) | 1.23 (0.41,3.65) | 1.00 | 1.23 (0.50,3.02) | 0.96 (0.38,2.44) | 0.99 (0.32,3.06) | 1.00 | 0.78 (0.31,1.94) | 0.97 (0.39,2.39) | 0.48 (0.14,1.70) |
| **APVR[b]** | 1.00 | 0.52 (0.23,1.20) | 0.66 (0.29,1.50) | 0.83 (0.30,2.30) | 1.00 | 0.84 (0.37,1.91) | 0.72 (0.30,1.69) | 0.59 (0.19,1.85) | 1.00 | 0.58 (0.23,1.49) | 0.92 (0.37,2.30) | 0.47 (0.14,1.53) |
| **AVSD[e]** | 1.00 | 0.40 (0.13,1.36) | 0.87 (0.28,2.68) | 0.51 (0.10, 2.70) | 1.00 | 1.32 (0.38,4.59) | 0.67 (0.17,2.62) | 1.20 (0.25, 5.91) | 1.00 | 2.84 (0.28, 28.9) | 2.18 (0.21,22.7) | 2.59 (0.21,32.7) |
| **RVOTO[b]** | 1.00 | 1.13 (0.68,1.86) | 1.27 (0.77,2.11) | 1.40 (0.75,2.62) | 1.00 | 1.32 (0.78,2.24) | 1.24 (0.72,2.14) | 1.85 (0.99,3.46) | 1.00 | 1.35 (0.74,2.45) | 1.10 (0.60,2.02) | 1.18 (0.58,2.38) |
| Pulmonary/tricuspid atresia[c] | 1.00 | 1.37 (0.59,3.21) | 0.85 (0.34,2.14) | 1.26 (0.43,3.68) | 1.00 | 1.49 (0.57,3.87) | 0.99 (0.36,2.73) | 1.70 (0.56,5.13) | 1.00 | 0.97 (0.36,2.63) | 1.05 (0.39,2.82) | 0.87 (0.27,2.81) |
| PVS[c] | 1.00 | 1.06 (0.60,1.85) | 1.41 (0.80,2.48) | 1.41 (0.69,2.89) | 1.00 | 1.20 (0.66,2.18) | 1.28 (0.70,2.34) | 1.89 (0.94,3.79) | 1.00 | 1.51 (0.76,3.00) | 1.08 (0.54,2.18) | 1.03 (0.46,2.31) |
| **Septal[b]** | 1.00 | 1.03 (0.69,1.52) | 1.20 (0.81,1.78) | 1.12 (0.69,1.81) | 1.00 | 1.06 (0.74,1.52) | 0.89 (0.61,1.30) | 0.73 (0.44,1.19) | 1.00 | 0.69 (0.47,1.03) | 0.64 (0.43,0.97) | 0.54 (0.32,0.90) |
| $VSD_{pm}$[c] | 1.00 | 0.80 (0.50,1.27) | 0.97 (0.60,1.56) | 0.95 (0.53,1.71) | 1.00 | 1.47 (0.89,2.43) | 1.09 (0.65,1.84) | 0.95 (0.50,1.83) | 1.00 | 0.65 (0.40,1.07) | 0.59 (0.36,0.98) | 0.67 (0.37,1.23) |
| ASD[c] | 1.00 | 1.36 (0.77,2.39) | 1.49 (0.84,2.64) | 1.32 (0.67,2.62) | 1.00 | 0.80(0.51,1.27) | 0.78 (0.49,1.25) | 0.62 (0.32,1.17) | 1.00 | 0.76 (0.45,1.29) | 0.70 (0.41,1.19) | 0.40 (0.19,0.83) |

Abbreviations: APVR-anomalous pulmonary venous return; ASD-atrial septal defect; AVSD-atrioventricular septal defect; CO-carbon monoxide; COA-coarctation of the aorta; d-TGA-d-transposition of the great arteries; HLHS-hypoplastic left heart syndrome; LVOTO-left ventricular outflow tract obstructions; $NO_2$-nitrogen dioxide; $O_3$-ozone; $PM_{10}$-particulate matter less than 10 microns in diameter; $PM_{2.5}$-particulate matter less than 2.5 microns in diameter; PVS-pulmonary valve stenosis; RVOTO-right ventricular outflow tract obstructions; $SO_2$-sulfur dioxide; TOF-tetralogy of Fallot; $VSD_{pm}$-perimembranous ventricular septal defects.

[a]Pollutant factors created using loadings resulting from the principal components analysis. Loadings are the relative weight of each of the original pollutant variables used to obtain the value of the computed factor. The loadings for each factor are: Factor 1: CO 85, $NO_2$ 71, $O_3$ -39, $PM_{10}$ 40, $PM_{2.5}$ 21, $SO_2$ 5; Factor 2: CO 11, $NO_2$ 6, $O_3$ 66, $PM_{10}$ 68, $PM_{2.5}$ 71, $SO_2$ -3; Factor 3: CO -11, $NO_2$ 25, $O_3$ -20, $PM_{10}$ -18, $PM_{2.5}$ 32, $SO_2$ 94. [b]Estimates results from a hierarchical regression model with first stage polytomous logistic model of defect groupings adjusted for maternal race, maternal age, maternal educational attainment, maternal household income, maternal smoking status and alcohol consumption during early pregnancy, nativity, and site-specific heart defect ratio. Defect-groupings include all individual defects listed underneath with the following additions: LVOTO also includes IAA-Type A, APVR includes total and partial APVR, and RVOTO includes Ebstein's Anomaly. Those defects could not be analyzed individually due to limited sample size. Septal grouping does not include muscular ventricular septal defects ($VSD_{muscular}$) as they were only collected in the first year of study when there was no available $PM_{2.5}$ data. [c]Estimates result from a hierarchical regression model with first stage polytomous logistic model of individual defects and adjusted for maternal race, maternal age, maternal educational attainment, maternal household income, maternal smoking status and alcohol consumption during early pregnancy, nativity, and site-specific heart defect ratio. [d]Other Conotruncal category includes common truncus, interrupted aortic-arch, type B and type not specified, double outlet right ventricle defects, and conoventricular septal defects. [e]Estimates result from model utilizing Firth's penalized maximum likelihood regression to deal with quasi-separation of points due to small sample size in certain cells. Model adjusted for maternal race, maternal age, maternal educational attainment, maternal household income, maternal smoking status and alcohol consumption during early pregnancy, nativity, and site-specific heart defect ratio. Excluded from the hierarchical analysis.

**Table S6:** Adjusted[a] odds ratios and 95% confidence intervals between congenital heart defects and 7-week average exposure to criteria air pollutants among participants who lived within 10 km of a stationary air monitor, National Birth Defects Prevention Study, 1997-2006.[b]

| Defect-Grouping | <10$^{th}$ centile | 10$^{th}$ -<50$^{th}$ centile | 50$^{th}$-<90$^{th}$ centile | ≥90$^{th}$ centile |
|---|---|---|---|---|
| **CO** | | | | |
| LVOTO | 1.00 | 1.38 (0.78,2.45) | 1.39 (0.78,2.47) | 1.25 (0.62,2.53) |
| Conotruncal | 1.00 | 1.19 (0.74,1.94) | 1.13 (0.69,1.85) | 1.43 (0.80,2.57) |
| APVR | 1.00 | 0.51 (0.18,1.42) | 0.56 (0.20,1.57) | 0.59 (0.16,2.18) |
| RVOTO | 1.00 | 0.99 (0.57,1.74) | 0.89 (0.50,1.57) | 0.70 (0.33,1.47) |
| Septal | 1.00 | 0.89 (0.59,1.36) | 1.03 (0.68,1.56) | 1.10 (0.66,1.82) |
| **$NO_2$** | | | | |
| LVOTO | 1.00 | 1.25 (0.54,2.88) | 1.37 (0.60,3.13) | 1.44 (0.58,3.61) |
| Conotruncal | 1.00 | 1.00 (0.49,2.04) | 1.20 (0.59,2.43) | 1.10 (0.49,2.48) |
| APVR | 1.00 | 0.22 (0.07,0.69) | 0.27 (0.09,0.81) | 0.56 (0.16,1.99) |
| RVOTO | 1.00 | 1.50 (0.52,4.34) | 1.39 (0.48,4.00) | 2.33 (0.75,7.22) |
| Septal | 1.00 | 1.01 (0.55,1.86) | 0.91 (0.50,1.67) | 1.12 (0.56,2.24) |
| **$O_3$[c]** | | | | |
| LVOTO | 1.00 | 1.47 (0.81,2.67) | 1.41 (0.78,2.56) | 1.62 (0.84,3.13) |
| Conotruncal | 1.00 | 1.18 (0.76,1.84) | 1.04 (0.67,1.63) | 0.88 (0.51,1.52) |
| APVR | 1.00 | 0.65 (0.21,2.02) | 1.20 (0.43,3.39) | 1.13 (0.33,3.84) |
| RVOTO | 1.00 | 1.61 (0.81,3.21) | 2.00 (1.02,3.91) | 1.52 (0.70,3.31) |
| Septal | 1.00 | 1.35 (0.87,2.09) | 1.25 (0.81,1.95) | 1.07 (0.63,1.85) |
| **$PM_{10}$** | | | | |
| LVOTO | 1.00 | 0.87 (0.48,1.57) | 1.13 (0.63,2.03) | 1.02 (0.51,2.03) |
| Conotruncal | 1.00 | 0.95 (0.54,1.66) | 1.09 (0.63,1.89) | 1.05 (0.56,1.97) |
| APVR | 1.00 | 1.37 (0.31,6.08) | 1.26 (0.28,5.57) | 1.20 (0.23,6.31) |
| RVOTO | 1.00 | 1.12 (0.72,1.76) | 1.01 (0.65,1.58) | 0.89 (0.52,1.53) |
| Septal | 1.00 | 1.26 (0.28,5.57) | 1.20 (0.23,6.31) | 1.20 (0.23,6.15) |
| **$PM_{2.5}$** | | | | |
| LVOTO | 1.00 | 1.18 (0.78,1.78) | 1.10 (0.71,1.68) | 1.57 (0.93,2.66) |
| Conotruncal | 1.00 | 0.95 (0.65,1.40) | 0.96 (0.65,1.43) | 1.43 (0.88,2.30) |
| APVR | 1.00 | 0.95 (0.41,2.19) | 0.87 (0.36,2.09) | 1.55 (0.54,4.46) |
| RVOTO | 1.00 | 1.17 (0.72,1.88) | 1.35 (0.84,2.18) | 1.21 (0.65,2.28) |
| Septal | 1.00 | 0.91 (0.68,1.22) | 0.67 (0.49,0.91) | 0.71 (0.45,1.10) |
| **$SO_2$** | | | | |
| LVOTO | 1.00 | 1.18 (0.58,2.40) | 1.29 (0.63,2.65) | 0.74 (0.30,1.83) |
| Conotruncal | 1.00 | 0.75 (0.40,1.42) | 0.75 (0.40,1.42) | 0.61 (0.29,1.31) |
| APVR | 1.00 | 1.95 (0.21,18.0) | 4.01 (0.45,35.5) | 1.00 (0.05,18.3) |
| RVOTO | 1.00 | 1.46 (0.70,3.04) | 0.93 (0.43,1.99) | 0.69 (0.27,1.74) |
| Septal | 1.00 | 1.75 (1.07,2.85) | 1.34 (0.80,2.25) | 1.52 (0.83,2.76) |

Abbreviations: APVR-anomalous pulmonary venous return; ASD-atrial septal defect; COA-coarctation of the aorta; d-TGA-d-transposition of the great arteries; HLHS-hypoplastic left heart syndrome; LVOTO-left ventricular outflow tract obstructions; $PM_{10}$-particulate matter less than 10 microns in diameter; $PM_{2.5}$-particulate matter less than 2.5 microns in diameter; PVS-pulmonary valve stenosis; RVOTO-right ventricular outflow tract obstructions; TOF-tetralogy of Fallot; $VSD_{muscular}$-muscular ventricular septal defects; $VSD_{pm}$-perimembranous ventricular septal defects.

[a]Estimates result from first stage maximum-likelihood, polytomous logistic model with defect groupings as outcomes and adjusted for maternal race, maternal age, maternal educational attainment, maternal household income, maternal smoking status and alcohol consumption during early pregnancy, nativity, and site-specific heart defect ratio. Same pollutant-cutoffs to create categories of exposure as primary 7-week analysis were used. Defect-groupings include: LVOTO- Aortic Stenosis, COA, HLHS, interrupted aortic arch-type A; Conotruncals-dTGA, TOF, common truncus, interrupted aortic arch-type B and type not specified, double outlet right ventricle defects, and conoventricular septal defects; APVR- total and partial APVR; RVOTO-pulmonary atresia, tricuspid atresia, PVS, and Ebstein's anomaly; Septal-$VSD_{pm}$, ASD, $VSD_{muscular}$, except for $PM_{2.5}$. $VSD_{muscular}$ were only collected in the first year of study when there was no available $PM_{2.5}$ data. [b]All results for the National Birth Defects Prevention Study population from 1997-2006, except for $PM_{2.5}$ which was 1999-2006 due to unavailability of monitoring data for $PM_{2.5}$ prior to 1999. [c]For ozone, the three categories of exposure were $25^{th}$ to less than the $50^{th}$ centile, $50^{th}$ centile to less than the $75^{th}$ centile, at or greater than the $75^{th}$ centile, with the referent grouping being below the $25^{th}$ centile.
